# The global burden of typhoid and paratyphoid fevers: a systematic analysis for the Global Burden of Disease Study 2017

**DOI:** 10.1016/S1473-3099(18)30685-6

**Published:** 2019-04

**Authors:** GBD 2017 Typhoid and Paratyphoid Collaborators, Jeffrey D Stanaway, Robert C Reiner, Brigette F. Blacker, Ellen M Goldberg, Ibrahim A. Khalil, Christopher E Troeger, Jason R Andrews, Zulfiqar A Bhutta, John A Crump, Justin Im, Florian Marks, Eric Mintz, Se Eun Park, Anita K M Zaidi, Zegeye Abebe, Ayenew Negesse Abejie, Isaac Akinkunmi Adedeji, Beriwan Abdulqadir Ali, Azmeraw T. Amare, Hagos Tasew Atalay, Euripide F G A Avokpaho, Umar Bacha, Aleksandra Barac, Neeraj Bedi, Adugnaw Berhane, Annie J Browne, Jesus L. Chirinos, Abdulaal Chitheer, Christiane Dolecek, Maysaa El Sayed Zaki, Babak Eshrati, Kyle J. Foreman, Abdella Gemechu, Rahul Gupta, Gessessew Bugssa Hailu, Andualem Henok, Desalegn Tsegaw Hibstu, Chi Linh Hoang, Olayinka Stephen Ilesanmi, Veena J Iyer, Amaha Kahsay, Amir Kasaeian, Tesfaye Dessale Kassa, Ejaz Ahmad Khan, Young-Ho Khang, Hassan Magdy Abd El Razek, Mulugeta Melku, Desalegn Tadese Mengistu, Karzan Abdulmuhsin Mohammad, Shafiu Mohammed, Ali H Mokdad, Jean B Nachega, Aliya Naheed, Cuong Tat Nguyen, Huong Lan Thi Nguyen, Long Hoang Nguyen, Nam Ba Nguyen, Trang Huyen Nguyen, Yirga Legesse Nirayo, Tikki Pangestu, George C Patton, Mostafa Qorbani, Rajesh Kumar Rai, Saleem M Rana, Chhabi Lal Ranabhat, Kedir Teji Roba, Nicholas L S Roberts, Salvatore Rubino, Saeid Safiri, Benn Sartorius, Monika Sawhney, Mekonnen Sisay Shiferaw, David L Smith, Bryan L. Sykes, Bach Xuan Tran, Tung Thanh Tran, Kingsley Nnanna Ukwaja, Giang Thu Vu, Linh Gia Vu, Fitsum Weldegebreal, Melaku Kindie Yenit, Christopher J L Murray, Simon I. Hay

## Abstract

**Background:**

Efforts to quantify the global burden of enteric fever are valuable for understanding the health lost and the large-scale spatial distribution of the disease. We present the estimates of typhoid and paratyphoid fever burden from the Global Burden of Diseases, Injuries, and Risk Factors Study (GBD) 2017, and the approach taken to produce them.

**Methods:**

For this systematic analysis we broke down the relative contributions of typhoid and paratyphoid fevers by country, year, and age, and analysed trends in incidence and mortality. We modelled the combined incidence of typhoid and paratyphoid fevers and split these total cases proportionally between typhoid and paratyphoid fevers using aetiological proportion models. We estimated deaths using vital registration data for countries with sufficiently high data completeness and using a natural history approach for other locations. We also estimated disability-adjusted life-years (DALYs) for typhoid and paratyphoid fevers.

**Findings:**

Globally, 14·3 million (95% uncertainty interval [UI] 12·5–16·3) cases of typhoid and paratyphoid fevers occurred in 2017, a 44·6% (42·2–47·0) decline from 25·9 million (22·0–29·9) in 1990. Age-standardised incidence rates declined by 54·9% (53·4–56·5), from 439·2 (376·7–507·7) per 100 000 person-years in 1990, to 197·8 (172·0–226·2) per 100 000 person-years in 2017. In 2017, *Salmonella enterica* serotype Typhi caused 76·3% (71·8–80·5) of cases of enteric fever. We estimated a global case fatality of 0·95% (0·54–1·53) in 2017, with higher case fatality estimates among children and older adults, and among those living in lower-income countries. We therefore estimated 135·9 thousand (76·9–218·9) deaths from typhoid and paratyphoid fever globally in 2017, a 41·0% (33·6–48·3) decline from 230·5 thousand (131·2–372·6) in 1990. Overall, typhoid and paratyphoid fevers were responsible for 9·8 million (5·6–15·8) DALYs in 2017, down 43·0% (35·5–50·6) from 17·2 million (9·9–27·8) DALYs in 1990.

**Interpretation:**

Despite notable progress, typhoid and paratyphoid fevers remain major causes of disability and death, with billions of people likely to be exposed to the pathogens. Although improvements in water and sanitation remain essential, increased vaccine use (including with typhoid conjugate vaccines that are effective in infants and young children and protective for longer periods) and improved data and surveillance to inform vaccine rollout are likely to drive the greatest improvements in the global burden of the disease.

**Funding:**

Bill & Melinda Gates Foundation.

## Introduction

Typhoid and paratyphoid fevers, collectively referred to as enteric fever, are caused by systemic infection with *Salmonella enterica* subspecies serovars Typhi and Paratyphi A, B, and C.^1^ Whereas most non-typhoidal *Salmonella* spp infections typically produce diarrhoeal illness and less commonly cause bloodstream infection, typhoid and paratyphoid infections produce primarily bacteraemic febrile illnesses, with prolonged high fever, headache, and malaise being characteristic symptoms. Without effective treatment, typhoid and paratyphoid fevers can lead to altered mental states (termed the typhoid state^2^), ileus, gastrointestinal bleeding, intestinal perforation, septic shock, and death.^1^, ^3^ Typhoid and paratyphoid infections are relatively common in countries with poor water supply and sanitation, especially south Asia, southeast Asia, and sub-Saharan Africa, where they are a major cause of death and disability, especially among children.^4^, ^5^, ^6^, ^7^

With growing antimicrobial resistance, and intensifying conversations around typhoid vaccine policy, accurate and detailed estimates of enteric fever burden are required.^8^ The Strategic Advisory Group of Experts (SAGE) of WHO convened in October, 2017, and recommended the use of typhoid conjugate vaccines in children between 6 months and 2 years of age, with a catch-up campaign for children up to 15 years of age, where possible, and WHO prequalified the first typhoid conjugate vaccine in December, 2017.^8^ These developments give typhoid-endemic, low-income countries priority access and funding for the vaccine and could make vaccines increasingly accessible. However, data to support their introduction remain sparse.^9^

**Research in context**

**Evidence before this study**

We searched PubMed for “(“typhoid” OR “paratyphoid” OR “typhi” OR “paratyphi”) AND (“burden” OR “estimates” OR “model”)”, with no restrictions placed on date of publication or language. We identified global (or near-global) estimates of enteric fever incidence, ranging from 12 million to 27 million cases annually. Estimates of mortality ranged from 129 000 to 223 000 deaths. Most previous studies assumed a case fatality of 1%, whereas one study estimated a case fatality range of 0·4% to 2·1%. Only previous iterations of the Global Burden of Diseases, Injuries, and Risk Factors Study (GBD) have reported estimates of temporal trends and health gap metrics for typhoid and paratyphoid fevers separately.

**Added value of this study**

We present estimates of the global burden of typhoid and paratyphoid fevers from GBD 2017, separately and collectively, by country, year, age, and sex. To our knowledge, this is the first study to provide estimates of typhoid and paratyphoid fever incidence, mortality, years of life lost (YLLs) to premature mortality, years lived with disability (YLDs), and disability-adjusted life-years (DALYs) at country, regional, and global levels, and the first study to provide global estimates of enteric fever trends. As such, we believe this analysis to be the most comprehensive picture of the burden of enteric fever to date. Trend estimates offer important information about the changing burden of the disease. Estimates of health gap metrics, which account for disease severity and give greater weight to deaths occurring at younger ages, allow for comparisons between diseases of differing severity and give policy makers the information required to set objective priorities.

**Implications of all the available evidence**

Growing antimicrobial resistance and national decision making about typhoid vaccine use heighten the need for accurate and detailed estimates of the global enteric fever burden. Our estimates, showing a decline in the number of typhoid and paratyphoid illnesses globally, from 25·9 million in 1990 to 14·3 million in 2017, are similar to previously published estimates that have ranged from about 12 million to 27 million. Still, the wide uncertainty around estimates and large differences in region-level estimates reflect the paucity of data and highlight the need for more widespread active surveillance of enteric fever. As countries move forward with vaccine roll-out, better data and better surveillance should be a priority.

Efforts to quantify the global burden of enteric fever have produced estimates ranging from 11·9 million to 27·1 million cases per year.^5^, ^6^, ^7^, ^10^, ^11^ These estimates are invaluable in understanding the health lost to enteric fever and in clarifying the large-scale spatial distribution of the disease. However, none of these studies produced country-level estimates separately for typhoid and paratyphoid fevers. Moreover, most studies have previously assumed a uniform case fatality of 1%^5^, ^6^, ^12^ and none has investigated the effects of age or income on case fatality. Finally, most studies have developed point estimates rather than trends.

With each iteration of the Global Burden of Diseases, Injuries, and Risk Factors Study (GBD),^13^, ^14^, ^15^, ^16^ we have improved our estimation methods and acquired additional data that have allowed us to address some of the shortcomings of previous enteric fever estimates. Here, we present estimates of the typhoid and paratyphoid fever burden from GBD 2017, separately and collectively, by country, year, age, and sex. In addition to estimating incidence and mortality, we present estimates of three health gap metrics: years of life lost (YLLs) to premature mortality, years lived with disability (YLDs), and disability-adjusted life-years (DALYs).

## Methods

### Overview

For this systematic analysis we modelled the combined incidence of typhoid and paratyphoid infections. We then modelled the proportion of this combined incidence that was attributable to typhoid fever and the proportion attributable to paratyphoid fever, and split total cases proportionally between the two. We estimated deaths using an ensemble model of vital registration data for locations with rich vital registration data and using a natural history approach for other locations. For the natural history model, we modelled case fatality by age and national income category and estimated deaths as the product of cases and case fatality. To propagate uncertainty through the modelling chain, we used posterior simulation, estimating 1000 draws from the posterior distribution of each estimate, and doing all calculations at the draw level. The code used in this analysis is available online.

### Incidence model

We estimated the total incidence of typhoid and para-​typhoid fevers from hybridised estimates of two models: a model of moderate and high-burden super-regions (incidence ≥10 per 100 000) based on only incidence data; and a model of low-burden super-regions (incidence <10 per 100 000) including both incidence data and estimates of incidence derived from vital registration sources (appendix pp 5–6). Incidence data were from population-based cohort studies and national surveillance systems. The systemic review, data availability maps, and all included sources are listed in the appendix (pp 3–4, 14, and 54–93). We excluded studies that reported data with the Widal test, given the unreliable performance characteristics of this diagnostic method. We assumed two major sources of under-reporting: first, imperfect blood culture sensitivity resulting in missed cases among those who were tested; and, second, incompleteness of data from passive national surveillance sources. We estimated blood culture sensitivity as 55% (95% CI 39–71)^17^, ^18^, ^19^ and adjusted reported case estimates by dividing by sensitivity, using posterior simulation to incorporate uncertainty from the sensitivity adjustment.

We modelled incidence using the Bayesian meta-regression tool DisMod-MR, which has been described elsewhere.^20^ DisMod-MR uses non-linear mixed-effects models to produce estimates by age, sex, year, and location. It uses age integration to accommodate data with disparate age categorisation schemes. Country-level covariates help inform estimates where data are sparse. The tool uses a Bayesian cascading geographical hierarchy to ensure that estimates have a strong spatial structure and to allow extrapolations to countries with no data. In addition to country-level covariates, DisMod accepts study-level covariates that allow data to be adjusted to account for known sources of bias and heterogeneity.

The incidence model used two country-level covariates. The first was the summary exposure value for unsafe water, which is a metric of the risk-weighted prevalence of exposure to unsafe water, weighted by the type of water supply,^21^ included because poor access to safe drinking water is a known risk factor for typhoid fever. The second was the proportion of the population living in the classic Indian Ocean monsoon belt,^22^ which was included both because typhoid fever incidence increases following monsoons^23^ and to prevent unreasonably high estimates in countries that have poor access to improved water supply but lie outside high-incidence regions, and for which few data are available (primarily countries in sub-Saharan Africa). These covariates were selected on the basis of disease epidemiology, the significance of their association with incidence (p<0·05), and non-collinearity (eg, sanitation was not included as a covariate because of its high collinearity with unsafe water). We used a study-level covariate identifying datapoints from passive national surveillance systems. We indicated active surveillance as the reference category and allowed DisMod-MR to adjust reported incidence rates from passive national surveillance sources to their expected values under the counterfactual scenario that they had been recorded via active surveillance (see appendix p 3 for details).

### Aetiology models

We estimated the aetiological breakdown of the typhoid–paratyphoid fever envelope using data from national surveillance, population-based cohort studies, and vital registration sources (appendix pp 59–60). Although studies typically classify cases as being typhoid or paratyphoid fever, sources rarely differentiate between Paratyphi A, B, and C. We assume, but cannot confirm, that Paratyphi A is the primary paratyphoid serovar. We used two DisMod-MR models: one for the proportion of the total cases caused by typhoid fever, and a second for the proportion of cases caused by paratyphoid fever. We rescaled each proportion estimate to ensure that the proportions due to typhoid and paratyphoid fever always summed to one. For each age-sex-year-location, and for each draw, the incidence of typhoid fever was calculated as the product of total incidence and the proportion of that total from typhoid fever; and the incidence of paratyphoid fever was calculated as the product of the total incidence and proportion of that total due to paratyphoid fever.

### Case fatality model

We modelled case fatality using notifiable disease and hospital data sources that reported both cases and deaths (see the appendix pp 90–93 for the full source list). We excluded sources that reported case fatality estimates from outbreaks as these estimates tend to be notably higher than those reported under the endemic conditions in which most cases occur. The scarcity of case fatality data and the small number of sources reporting age-specific case fatality precluded the use of a single model to estimate case fatality by country, age, and year. Instead, we first used the age-integration abilities of DisMod-MR using only age-specific data to estimate a global age pattern. Second, we did a meta-analysis of mean all-age case fatality data for three income groups: high-income countries (World Bank “high income, OECD” and “high income, non-OECD” countries), upper-middle-income countries (equivalent to the World Bank classification), and low-income countries (World Bank “low-income” and “lower-middle income” countries).^24^ We then applied the global age pattern to the mean case fatality estimates for each income category to derive age-specific case fatality estimates for each of the three income categories. We estimated the relative risk of fatality among typhoid fever cases compared with paratyphoid fever cases using a single large dataset that reported case fatality separately for typhoid and paratyphoid fevers.^25^ Finally, we applied this relative risk to our overall case fatality estimate to derive separate estimates of case fatality for typhoid and paratyphoid fevers. As with our estimates of incidence, we used posterior simulation to incorporate uncertainty from the average case fatality, age-pattern, and the relative fatality of typhoid and paratyphoid fevers (see appendix pp 9–10 for details).

### Mortality estimation

GBD uses a star-rating system from zero to five to represent the quality and completeness of cause of death (CoD) data, primarily vital registration data, for each location, during the GBD estimation period.^26^ For locations with four or five star CoD data (see appendix pp 10–11 for details), we estimated deaths directly from vital registration data using the CoD ensemble modelling (CODEm) tool, which has been described elsewhere.^26^ Briefly, CODEm estimates a set of plausible submodels, tests each for out-of-sample predictive validity, and produces final estimates from an ensemble derived from weighted combinations of submodels. For locations with lower than four-star CoD data, we considered the data insufficiently complete and used a natural history approach in which we estimated typhoid fever mortality as the product of typhoid fever incidence and case fatality. We estimated paratyphoid fever mortality as the product of paratyphoid fever incidence and case fatality. One fundamental principle of GBD is that the sum of all cause-specific mortality estimates should equal all-cause mortality within each age, sex, year, and location. We impose this consistency through CoDCorrect, a process in which we rescale cause-specific mortality estimates to fit the all-cause mortality envelope.^26^

### Estimation of health gap metrics

We estimated three health gap metrics: YLLs, which quantify fatal health loss according to a reference life expectancy at the age of death (ie, deaths at younger ages result in more YLLs); YLDs, which quantify non-fatal health loss accounting for both the duration and severity of a condition; and DALYs, which are the sum of YLLs and YLDs, and quantify fatal and non-fatal health loss combined. YLLs were calculated with methods described previously.^26^

To calculate YLDs, we assigned cases proportionally across different sequelae, each with an associated duration and disability weight. We split typhoid fever cases between four sequelae: moderate typhoid fever, severe uncomplicated typhoid fever, severe typhoid fever with gastrointestinal bleeding, and severe typhoid fever with other complications (including, but not limited to, intestinal perforation).^27^ Paratyphoid fever cases were split between mild paratyphoid fever, moderate paratyphoid fever, severe paratyphoid fever, and paratyphoid fever with abdominal complications. Each sequela was assigned the disability of the most closely matching health state from the 235 health states in GBD 2017.^4^ We assumed the duration of illness was 14 days (95% UI 7–21) for mild or moderate sequelae, and 28 days (14–49) with severe or complicated sequelae.^28^, ^29^, ^30^, ^31^ YLDs from a given sequela were calculated as the product of the number of incident cases of that sequela, its duration in years, and the corresponding disability weight. Total YLDs for typhoid fever were calculated as the sum of the YLDs from each of its four sequelae, and total YLDs for paratyphoid fever were calculated as the sum of the YLDs from each of its four sequelae. Finally, we adjusted YLDs through a comorbidity correction that ensures that comorbidities cannot result in an aggregate disability weight greater than one (equivalent to death).^4^

### Role of the funding source

AKMZ, an employee of the funder of this study, helped acquire data and provided feedback and edits on the manuscript. The sponsor of this study had no other role in study design, data collection, data analysis, data interpretation, or writing of the report. The corresponding author had full access to all the data in the study and had final responsibility for the decision to submit for publication.

## Results

**Figure 1:**
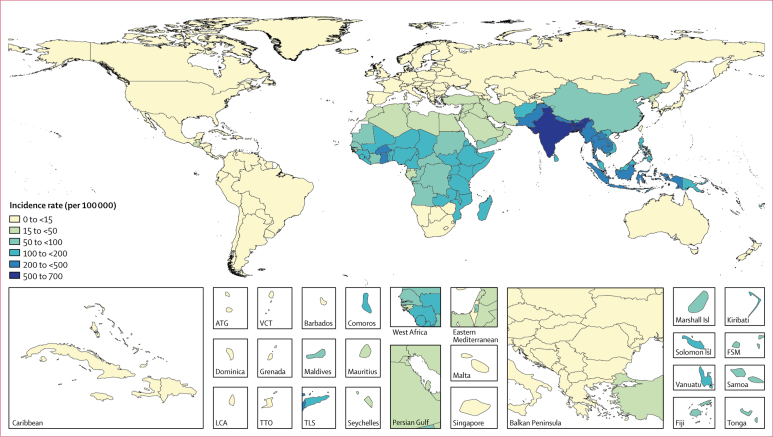
Incidence rates (per 100 000) of typhoid and paratyphoid fevers, by country, in 2017 Unfilled locations are those for which GBD does not produce estimates. The inset maps detail smaller locations. ATG=Antigua and Barbuda. FSM=Federated States of Micronesia. GBD=Global Burden of Diseases, Injuries, and Risk Factors Study. Isl=Islands. LCA=Saint Lucia. TLS=Timor-Leste. TTO=Trinidad and Tobago. VCT=Saint Vincent and the Grenadines.

We estimate that 14·3 million (95% uncertainty interval [UI] 12·5–16·3) cases of typhoid and paratyphoid fevers occurred globally in 2017, a 44·6% (42·2–47·0) decline from 25·9 million (22·1–29·9) cases in 1990 (table 1). Age-standardised incidence rates declined by 54·9% (53·4–56·5), from 439·2 (376·7–507·7) per 100 000 person-years in 1990 to 197·8 (172·0–226·2) per 100 000 person-years in 2017. Comparing GBD super-regions, south Asia had the highest age-standardised incidence rate (549 [481–625] cases per 100 000 person-years) and the largest number of cases (10·3 million [9·0–11·7]), accounting for 71·8% of global cases in 2017. The southeast Asia, east Asia, and Oceania super-region accounted for 14·1% of global cases (2·02 million [1·82–2·23]), and the sub-Saharan Africa super-region accounted for 12·1% (1·73 million [1·45–2·06]; figure 1, table 2). Of all cases globally, 76·3% (71·8–80·5) were attributable to *S* Typhi (table 3). Thus, in 2017, we estimate that there were 10·9 million (9·3–12·6) cases of typhoid fever and 3·4 million (2.7–4·2) cases of paratyphoid fever globally (table 3).

**Table 1:** Global numbers of cases, deaths, years of life lost, years lived with disability, and disability-adjusted life-years (in thousands) by year for typhoid and paratyphoid fevers

	**Cases**	**Deaths**	**YLLs**	**YLDs**	**DALYs**
**Paratyphoid fever only**					
1990	5508 (4233–7086)	28·5 (12·7–56·7)	2071 (913–4172)	15·0 (9·4–22·6)	2086 (923–4199)
1995	5139 (3963–6543)	27·3 (12·4–53·8)	1977 (888–3912)	14·0 (8·8–21·0)	1991 (897–3931)
2000	4698 (3640–5953)	25·2 (11·5–49·9)	1817 (835–3600)	12·9 (8·1–19·5)	1830 (845–3619)
2005	4232 (3311–5327)	23·0 (10·5–45·3)	1649 (756–3259)	11·6 (7·3–17·3)	1661 (762–3274)
2010	3794 (2992–4739)	20·9 (9·5–40·6)	1477 (670–2862)	10·4 (6·7–15·5)	1487 (679–2881)
2017	3397 (2666–4184)	19·1 (8·7–37·3)	1354 (622–2620)	9·4 (5·9–13·9)	1364 (631–2641)
**Typhoid fever only**					
1990	20 366 (17 117–23 882)	202·0 (112·5–327·1)	14 954 (8330–24 474)	192·7 (128·8–277·2)	15 147 (8493–24 777)
1995	18 424 (15 562–21 584)	185·4 (104·2–302·6)	13 693 (7658–22 281)	174·9 (117·9–254·6)	13 868 (7801–22 501)
2000	16 797 (14 229–19 550)	171·6 (96·6–278·9)	12 598 (7138–20 572)	160·0 (108·1–227·6)	12 758 (7280–20 723)
2005	15 415 (13 131–17 894)	160·2 (89·8–262·9)	11 712 (6582–19 081)	147·3 (97·6–213·2)	11 860 (6694–19 317)
2010	13 769 (11 739–15 938)	145·4 (80·9–236·0)	10 556 (5902–17 048)	132·2 (88·3–188·4)	10 688 (6028–17 188)
2017	10 924 (9343–12 597)	116·8 (65·4–187·7)	8332 (4632–13 419)	105·5 (70·3–151·0)	8437 (4731–13 577)
**Typhoid and paratyphoid fevers combined**					
1990	25 875 (22 073–29 915)	230·5 (131·2–372·6)	17 026 (9768–27 546)	207·7 (140·0–299·2)	17 233 (9931–27 789)
1995	23 563 (20 198–27 338)	212·7 (120·8–341·5)	15 671 (8845–25 182)	189·0 (127·6–273·5)	15 860 (9016–25 474)
2000	21 495 (18 521–24 788)	196·8 (113·0–315·9)	14 415 (8329–23 159)	172·8 (117·1–245·0)	14 588 (8422–23 327)
2005	19 648 (16 992–22 576)	183·2 (104·6–293·4)	13 362 (7649–21 673)	158·9 (106·0–229·1)	13 521 (7780–21 847)
2010	17 564 (15 295–20 086)	166·3 (95·2–264·5)	12 033 (6907–19 424)	142·7 (95·4–202·7)	12 175 (7033–19 654)
2017	14 321 (12 540–16 337)	135·9 (76·9–218·9)	9686 (5485–15 746)	114·9 (77·7–164·2)	9801 (5569–15 880)

95% uncertainty intervals are included in parentheses. YLL=years of life lost. YLD=years lived with disability. DALY=disability-adjusted life-years.

**Table 2:** Typhoid and paratyphoid fevers and age-standardised incidence rates by GBD region, for 1990 and 2017, and the percentage change in age-standardised incidence rates between 1990 and 2017

		**1990**		**2017**		**Percentage change**
		Cases (thousands)	Incidence (per 100 000)	Cases (thousands)	Incidence (per 100 000)	
Global		25 875 (22 073 to 29 915)	439·2 (376·7 to 507·7)	14 321 (12 540 to 16 337)	197·8 (172·0 to 226·2)	−54·9% (−56·5 to −53·4)
Central Europe, eastern Europe, and central Asia						
	Central Asia	0·1 (0·1 to 0·1)	0·2 (0·1 to 0·2)	0·1 (0·1 to 0·1)	0·1 (0·1 to 0·1)	−27·8% (−35·2 to −21·2)
	Central Europe	1·7 (1·3 to 2·2)	1·2 (1·0 to 1·5)	1·2 (0·9 to 1·5)	0·7 (0·6 to 0·9)	−41·2% (−46·1 to −35·8)
	Eastern Europe	1·2 (0·9 to 1·5)	0·5 (0·4 to 0·6)	1·1 (0·9 to 1·5)	0·4 (0·3 to 0·5)	−9·3% (−17·4 to −0·9)
High income						
	Australasia	0·0 (0·0 to 0·0)	0·2 (0·1 to 0·2)	0·0 (0·0 to 0·1)	0·2 (0·1 to 0·2)	−4·7% (−16·2 to 7·6)
	High-income Asia Pacific	0·9 (0·7 to 1·1)	0·5 (0·4 to 0·6)	0·7 (0·6 to 0·9)	0·3 (0·2 to 0·3)	−45·4% (−52·1 to −37·9)
	High-income North America	4·8 (4·1 to 5·6)	1·6 (1·3 to 1·8)	4·1 (3·2 to 5·3)	0·9 (0·7 to 1·2)	−41·8% (−48·4 to −34·7)
	Southern Latin America	1·6 (1·3 to 1·9)	3·2 (2·7 to 3·8)	0·5 (0·4 to 0·6)	0·7 (0·5 to 0·9)	−79·2% (−82·8 to −75·1)
	Western Europe	1·8 (1·4 to 2·2)	0·4 (0·3 to 0·5)	1·3 (1·0 to 1·6)	0·3 (0·2 to 0·4)	−28·8% (−35·2 to −21·4)
Latin America and Caribbean						
	Andean Latin America	0·7 (0·6 to 1·0)	2·4 (1·8 to 3·1)	0·9 (0·7 to 1·1)	1·5 (1·2 to 2·0)	−35·4% (−40·9 to −29·6)
	Caribbean	3·0 (2·5 to 3·5)	8·0 (6·7 to 9·5)	2·5 (2·1 to 3·0)	5·7 (4·7 to 6·7)	−29·3% (−32·4 to −26·1)
	Central Latin America	29·7 (24·9 to 35·7)	23·1 (19·2 to 29·0)	20·0 (16·6 to 24·6)	8·2 (6·9 to 10·2)	−64·3% (−67·5 to −60·5)
	Tropical Latin America	3·8 (2·9 to 5·1)	2·8 (2·2 to 3·6)	4·3 (3·3 to 5·5)	1·9 (1·5 to 2·4)	−33·2% (−38·2 to −29·4)
North Africa and Middle East		531·0 (453·5 to 615·1)	132·1 (115·2 to 150·6)	246·6 (210·8 to 286·3)	39·3 (33·7 to 45·6)	−70·2% (−71·1 to −69·3)
South Asia		19 991 (16 982 to 23 298)	1506·8 (1308·4 to 1729·6)	10 286 (9002 to 11 738)	549·2 (480·7 to 625·4)	−63·5% (−64·9 to −62·3)
Southeast Asia, east Asia, and Oceania						
	East Asia	728·4 (652·9 to 819·4)	55·6 (49·9 to 62·9)	586·6 (553·8 to 624·0)	51·0 (47·3 to 55·1)	−8·3% (−16·9 to −3·6)
	Oceania	29·3 (24·2 to 35·3)	381·3 (322·8 to 448·3)	20·5 (17·0 to 24·7)	144·3 (121·5 to 170·4)	−62·2% (−63·6 to −60·7)
	Southeast Asia	3028·8 (2606·1 to 3492·8)	562·0 (492·1 to 643·8)	1414·4 (1247·9 to 1592·9)	219·8 (192·9 to 249·1)	−60·9% (−61·7 to −59·9)
Sub-Saharan Africa						
	Central sub-Saharan Africa	86·3 (69·9 to 105·8)	127·2 (108·0 to 150·8)	122·4 (100·2 to 149·1)	81·4 (68·8 to 95·8)	−36·0% (−38·0 to −33·7)
	Eastern sub-Saharan Africa	584·6 (489·0 to 703·5)	243·8 (210·6 to 283·6)	739·5 (627·6 to 869·7)	151·9 (132·0 to 174·6)	−37·7% (−38·9 to −36·5)
	Southern sub-Saharan Africa	2·2 (1·9 to 2·5)	3·5 (3·1 to 4·0)	1·9 (1·7 to 2·1)	2·3 (2·0 to 2·6)	−33·9% (−35·6 to −32·3)
	Western sub-Saharan Africa	843·4 (690·2 to 1027·5)	354·6 (301·7 to 418·3)	866·1 (719·7 to 1039·6)	161·1 (138·1 to 187·3)	−54·5% (−55·9 to −53·2)

95% uncertainty intervals are included in parentheses. GBD regions are detailed in the appendix. GBD=Global Burden of Diseases, Injuries, and Risk Factors Study.

**Table 3:** Breakdown of cases and deaths by cause (typhoid and paratyphoid fever) by GBD region, for 2017

		**Cases (thousands)**			**Deaths**		
		Typhoid fever	Paratyphoid fever	Percentage typhoid fever	Typhoid fever	Paratyphoid fever	Percentage typhoid fever
Global		10 924·3 (9343·0–12 597·1)	3396·9 (2666·5–4184·1)	76·3% (71·8–80·5)	116 815 (65 421–187 652)	19 108 (8706–37 332)	85·9% (77·7–91·9)
Central Europe, eastern Europe, and central Asia							
	Central Asia	0·1 (0·1–0·1)	0·0 (0·0–0·0)	81·0% (75·9–86·0)	1 (1–1)	1 (0–3)	48·9% (22·2–87·2)
	Central Europe	0·1 (0·1–0·1)	1·1 (0·8–1·4)	9·3% (7·8–11·2)	2 (1–4)	1 (1–2)	62·6% (47·0–80·1)
	Eastern Europe	0·6 (0·4–0·8)	0·5 (0·4–0·7)	52·7% (47·6–58·2)	1 (0–1)	1 (0–1)	41·8% (27·0–62·7)
High income							
	Australasia	0·0 (0·0–0·0)	0·0 (0·0–0·1)	13·3% (10·5–16·5)	0 (0–0)	0 (0–0)	39·2% (14·0–58·2)
	High-income Asia Pacific	0·1 (0·1–0·1)	0·6 (0·5–0·8)	14·5% (12·6–16·6)	0 (0–1)	1 (0–2)	34·1% (18·0–73·8)
	High-income North America	0·6 (0·4–0·8)	3·5 (2·8–4·6)	14·3% (12·6–16·3)	0 (0–1)	2 (1–7)	18·6% (6·2–30·5)
	Southern Latin America	0·2 (0·1–0·3)	0·3 (0·2–0·4)	41·1% (34·6–47·6)	3 (2–6)	0 (0–0)	97·7% (92·8–99·0)
	Western Europe	0·1 (0·1–0·2)	1·1 (0·9–1·4)	11·5% (9·0–14·4)	12 (7–16)	2 (0–4)	84·3% (77·1–96·4)
Latin America and Caribbean							
	Andean Latin America	0·9 (0·7–1·1)	0·0 (0·0–0·0)	97·8% (97·0–98·5)	7 (3–13)	0 (0–0)	98·8% (97·8–99·4)
	Caribbean	0·9 (0·7–1·2)	1·6 (1·3–1·9)	37·7% (31·7–44·3)	11 (5–20)	9 (4–17)	56·3% (40·7–70·4)
	Central Latin America	19·3 (16·0–23·7)	0·7 (0·6–1·0)	96·4% (95·6–97·0)	97 (59–249)	1 (1–5)	98·3% (94·3–99·6)
	Tropical Latin America	4·2 (3·3–5·4)	0·1 (0·1–0·1)	97·6% (96·8–98·3)	30 (14–54)	0 (0–1)	98·7% (97·6–99·3)
North Africa and Middle East		239·4 (205·0–277·5)	7·2 (5·0–10·3)	97·1% (96·0–97·9)	2 731 (1458–4752)	43 (17–94)	98·4% (97·2–99·2)
South Asia		7478·0 (6350·9–8665·0)	2808·3 (2194·4–3478·8)	72·7% (67·4–77·7)	78 892 (44 214–125 372)	15 773 (7319–30 611)	83·4% (73·9–90·3)
Southeast Asia, east Asia, and Oceania							
	East Asia	378·3 (342·9–416·2)	208·3 (177·7–241·2)	64·5% (59·4–69·4)	2845 (1416–4991)	858 (361–1702)	76·9% (65·3–86·0)
	Oceania	16·2 (13·3–19·5)	4·3 (3·1–5·8)	79·0% (74·0–83·6)	195 (100–350)	29 (12–60)	87·2% (79·4–92·9)
	Southeast Asia	1284·6 (1132·7–1448·2)	129·8 (98·4–167·5)	90·8% (88·5–92·7)	12 090 (6822–19 869)	705 (305–1405)	94·5% (90·4–97·0)
Sub-Saharan Africa							
	Central sub-Saharan Africa	119·2 (97·2–145·1)	3·2 (2·1–4·6)	97·4% (96·5–98·2)	1236 (634–2188)	18 (7–41)	98·5% (97·4–99·2)
	Eastern sub-Saharan Africa	726·4 (615·6–856·5)	13·1 (9·4–18·1)	98·2% (97·6–98·7)	9893 (5240–16 866)	93 (38–184)	99·1% (98·3–99·5)
	Southern sub-Saharan Africa	1·7 (1·5–2·0)	0·1 (0·1–0·2)	92·2% (90·1–94·0)	18 (10–32)	1 (0–2)	95·5% (92·3–97·6)
	Western sub-Saharan Africa	653·2 (534·8–794·0)	212·9 (158·2–278·3)	75·4% (70·2–80·0)	8751 (4481–15 514)	1568 (640–3306)	84·8% (76·2–91·4)

95% uncertainty intervals are included in parentheses. GBD=Global Burden of Diseases, Injuries, and Risk Factors Study.

**Figure 2:**
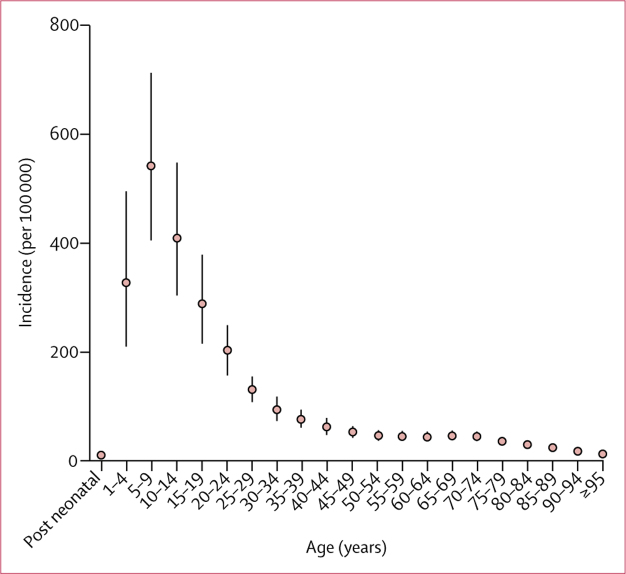
Global age-specific incidence rates (per 100 000) of typhoid and paratyphoid fevers in 2017

Incidence rates were highest among children, peaking in the 5–9-year age group and declining steadily into adulthood (figure 2). In 2017, 12·6% (8·7–17·7) of cases occurred among children younger than 5 years, and 55·9% (50·3–61·6) occurred among children younger than 15 years of age (appendix pp 15–16). We found differing age patterns in high-incidence and low-incidence regions, with incidence concentrated among children in high-incidence regions, and distributed more broadly across age groups in low-incidence regions (appendix p 17). 56·3% (51·8–61·2) of cases occurred among males.

Mean all-age global case fatality was 0·95% (0·54–1·53), with higher case fatality estimates among children and older adults, and among those living in lower-income countries (appendix pp 22–25). Case fatality for typhoid fever was 1·89 times (1·09–3·28) that for paratyphoid fever. Cause, age, and income-specific case fatality estimates for paratyphoid fever ranged from a low of 0·3% (0·1–0·8) among adults in high-income countries to a high of 0·9% (0·4–1·9) among post-neonates (28–364 days) in lower-income countries; and those for typhoid fever ranged from 0·6% (0·2–1·3) among adults in high-income countries to 1·6% (0·8–3·0) among post-neonates in lower-income countries (appendix p 25).

**Figure 3:**
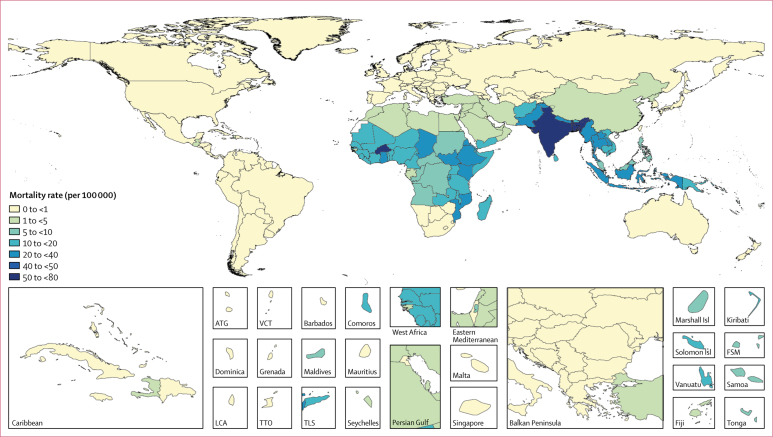
Typhoid and paratyphoid fever mortality rates (per million), by country, in 2017 Unfilled locations are those for which GBD does not produce estimates. The inset maps detail smaller locations. ATG=Antigua and Barbuda. FSM=Federated States of Micronesia. GBD=Global Burden of Diseases, Injuries, and Risk Factors Study. Isl=Islands. LCA=Saint Lucia. TLS=Timor-Leste. TTO=Trinidad and Tobago. VCT=Saint Vincent and the Grenadines.

**Figure 4:**
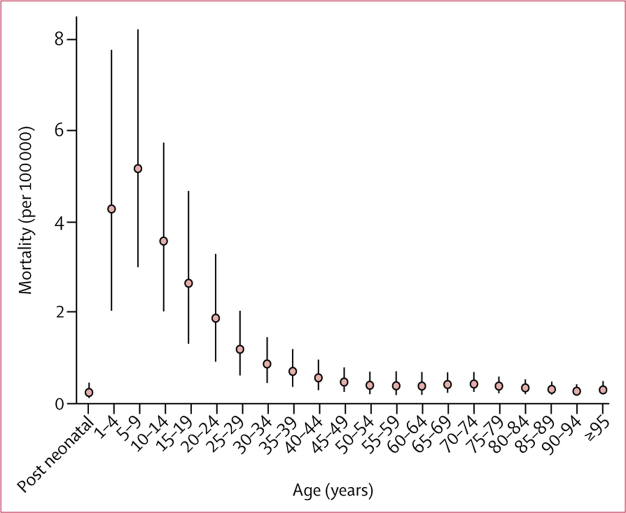
Global age-specific mortality rates (per 100 000) from typhoid and paratyphoid fevers in 2017

We estimated 135·9 thousand (76·9–218·9) deaths from typhoid and paratyphoid fever globally in 2017, a 41·0% (33·6–48·3) decline from 230·5 thousand (131·2–372·6) in 1990 (table 1). As with cases, south Asia had the highest mortality rates and highest absolute number of deaths, accounting for 69·6% (94·7 thousand [54·4–135·2]) of global deaths from typhoid and paratyphoid fever in 2017, followed by the sub-Saharan Africa super-region (21·6 thousand [11·3–38·1]; 15·9% of global deaths), and the southeast Asia, east Asia, and Oceania super-region (16·7 thousand [9·38–27·7]; 12·3% of global deaths; figure 3, appendix p 26). Mortality rates were highest among young children, peaking among those aged 5–9 years, and steadily declined with age (figure 4). In 2017, 17·2% (12·3–23·4) of deaths occurred among children younger than 5 years of age, and 59·3% (53·6–65·2%) occurred among children younger than 15 years of age. Of all deaths globally, 85·9% (77·7–91·9) were attributable to *S* Typhi, resulting in 116·8 thousand (65·4–187·7) deaths from typhoid fever and 19·1 thousand (8·7–37·3) from paratyphoid fever in 2017 (table 3). 74·0 thousand (42·1–120·1) deaths occurred among males compared with 61·9 thousand (34·7–101·4) deaths among females.

The 10·9 million cases of typhoid fever and 3·4 million cases of paratyphoid fever in 2017 resulted in 105·5 thousand (70·3–151·0) YLDs attributable to typhoid fever and 9·4 thousand (5·9–13·9) YLDs attributable to paratyphoid fever. Similarly, the 116·8 thousand deaths due to typhoid fever and 19·1 thousand deaths due to paratyphoid fever in 2017, resulted in 8·3 million (4·6–13·4) YLLs attributable to typhoid fever and 1·3 million (0·6–2·6) attributable to paratyphoid fever. Overall, typhoid and paratyphoid fever were responsible for 9·8 million (5·6–15·8) DALYs in 2017, down 43·0% (35·5–50·6) from 17·2 million (9·9–27·8) DALYs in 1990 (table 4). In 2017, 20·5% (14·8–27·5) of DALYs occurred among children younger than 5 years of age, and 67·0% (61·6–72·4) occurred among children younger than 15 years of age. In 2017, 5·35 million (3·01–8·59) DALYs occurred among males, and 4·45 million (2·52 −7·31) DALYs occurred among females.

**Table 4:** Years of life lost, years lived with disability, and disability-adjusted life-years due to typhoid and paratyphoid fevers by GBD region, for 2017

		**YLLs**		**YLDs**		**DALYs**	
		Number (thousands)	Rate (per million)	Number (thousands)	Rate (per million)	Number (thousands)	Rate (per million)
Global		9686·1 (5484·9–15 746·2)	1363·2 (770·0–2209·0)	114·88 (77·73–164·22)	15·87 (10·69–22·71)	9801 (5579–15 839)	1379·1 (784·3–2233·0)
Central Europe, eastern Europe, and central Asia							
	Central Asia	0·1 (0·0–0·1)	0·9 (0·4–1·5)	0·001 (0·000–0·001)	0·01 (0·01–0·01)	0·1 (0·0–0·1)	0·9 (0·4–1·5)
	Central Europe	0·1 (0·0–0·1)	0·5 (0·3–0·8)	0·004 (0·003–0·006)	0·03 (0·02–0·04)	0·1 (0·1–0·1)	0·5 (0·3–0·8)
	Eastern Europe	0·0 (0·0–0·0)	0·1 (0·1–0·2)	0·007 (0·004–0·011)	0·03 (0·02–0·04)	0·0 (0·0–0·1)	0·2 (0·1–0·2)
High income							
	Australasia	0·0 (0·0–0·0)	0·0 (0·0–0·1)	0·000 (0·000–0·000)	0·01 (0·00–0·01)	0·0 (0·0–0·0)	0·0 (0·0–0·1)
	High-income Asia Pacific	0·0 (0·0–0·1)	0·1 (0·1–0·3)	0·003 (0·002–0·004)	0·01 (0·01–0·01)	0·0 (0·0–0·1)	0·1 (0·1–0·3)
	High-income North America	0·1 (0·1–0·3)	0·3 (0·2–0·9)	0·016 (0·010–0·023)	0·03 (0·02–0·05)	0·1 (0·1–0·3)	0·3 (0·2–0·9)
	Southern Latin America	0·1 (0·1–0·1)	1·2 (0·8–2·0)	0·003 (0·002–0·004)	0·04 (0·02–0·06)	0·1 (0·1–0·1)	1·2 (0·8–2·1)
	Western Europe	0·2 (0·1–0·3)	0·3 (0·2–0·4)	0·005 (0·003–0·007)	0·01 (0·01–0·02)	0·2 (0·1–0·3)	0·3 (0·2–0·4)
Latin America and Caribbean							
	Andean Latin America	0·3 (0·2–0·6)	5·5 (2·7–10·5)	0·009 (0·005–0·013)	0·15 (0·09–0·22)	0·3 (0·2–0·7)	5·7 (2·9–10·6)
	Caribbean	1·4 (0·7–2·5)	32·4 (15·9–60·3)	0·014 (0·009–0·020)	0·31 (0·20–0·46)	1·4 (0·7–2·5)	32·7 (16·2–60·6)
	Central Latin America	3·7 (2·4–9·0)	15·0 (9·6–36·6)	0·19 (0·13–0·28)	0·79 (0·51–1·14)	3·9 (2·6–9·2)	15·8 (10·2–37·8)
	Tropical Latin America	1·4 (0·7–2·6)	6·4 (3·0–11·9)	0·04 (0·03–0·06)	0·18 (0·11–0·27)	1·5 (0·7–2·6)	6·6 (3·2–12·1)
North Africa and Middle East		196·7 (104·3–345·6)	309·4 (164·3–544·1)	2·38 (1·56–3·48)	3·80 (2·50–5·53)	199·1 (106·6–347·6)	313·2 (167·9–547·2)
South Asia		6737·5 (3894·5–10 976·9)	3571·8 (2054·4–5791·6)	79·47 (53·40–114·18)	42·48 (28·67–60·67)	6817 (3958–11 097)	3614 (2095–5856)
Southeast Asia, east Asia, and Oceania							
	East Asia	237·7 (122·0–415·6)	234·4 (122·2–410·1)	4·30 (2·93–6·05)	3·70 (2·46–5·31)	242·0 (126·5–419·1)	238·1 (125·9–414·2)
	Oceania	16·2 (8·3–28·7)	1082·0 (559·3–1928·3)	0·17 (0·11–0·26)	12·06 (7·69–17·84)	16·3 (8·5–28·9)	1094·1 (569·1–1942·8)
	Southeast Asia	864·3 (488·9–1412·0)	1358·7 (770·0–2228·3)	12·92 (8·66–18·50)	20·07 (13·48–28·68)	877·2 (502·4–1424·3)	1378·7 (792·4–2244·9)
Sub-Saharan Africa							
	Central sub-Saharan Africa	93·9 (47·8–165·7)	569·7 (292·9–1007·4)	1·18 (0·75–1·75)	7·88 (5·14–11·48)	95·1 (48·7–166·8)	577·6 (300·0–1013·5)
	Eastern sub-Saharan Africa	749·3 (397·3–1279·1)	1392·7 (738·9–2378·6)	7·16 (4·66–10·61)	14·71 (9·70–21·19)	756·5 (405·2–1286·6)	1407·4 (755·1–2396·0)
	Southern sub-Saharan Africa	1·4 (0·7–2·5)	16·9 (8·9–30·2)	0·02 (0·01–0·03)	0·21 (0·14–0·31)	1·4 (0·8–2·5)	17·1 (9·1–30·4)
	Western sub-Saharan Africa	781·7 (399·5–1408·5)	1303·3 (677·9–2307·3)	6·99 (4·42–10·39)	13·08 (8·50–19·14)	788·7 (404·6–1419·7)	1316·4 (686·4–2318·8)

95% uncertainty intervals are included in parentheses. YLL=years of life lost. YLD=years lived with disability. DALY=disability-adjusted life-years. GBD=Global Burden of Diseases, Injuries, and Risk Factors Study.

## Discussion

We present comprehensive global estimates of typhoid and paratyphoid fever by country, age, year, and sex. Our estimates show the number of typhoid and paratyphoid fever cases declining globally, from 25·9 million in 1990 to 14·3 million in 2017. Given the limited use of typhoid vaccine in typhoid-endemic countries, these declines likely reflect the broad improvements that come with economic development, including infrastructure improvements (eg, water supply and sanitation), and improved food handling practices, as well increased access to antibiotic treatment.

Our global estimates of cases and deaths are comparable to previously published estimates (appendix p 19). Crump and colleagues^6^ estimated that, in 2000, there were 21·7 million cases of typhoid fever (with results from a sensitivity analysis ranging from 10·8 million to 43·3 million), 5·4 million cases of paratyphoid fever, and 216 510 deaths from typhoid fever (sensitivity analysis range of 21 651 to 1·1 million). Buckle and colleagues,^5^ after accounting for low diagnostic sensitivity, estimated 26·9 million (IQR 18·3–35·7) cases in 2010. Mogasale and colleagues^7^ published two sets of estimates for low-income and middle-income countries for 2010: first, an estimate of 11·9 million (95% CI 9·9–14·7) cases and 129 thousand (95% CI 75–208) deaths, derived from a model that included an adjustment for water-related risk at each study site; and, second, unadjusted estimates of 20·6 million (95% CI 17·5–24·2) cases and 223 thousand (95% CI 131–344) deaths. Kim and colleagues^11^ updated the estimates produced by Mogasale and colleagues to include data from the Typhoid Fever Surveillance in Africa Program (TSAP), resulting in a slight upward revision to 12·1 million (95% CI 10·0–14·8) cases from the adjusted model and 20·8 million (17·8–24·2) from the unadjusted model. Finally, Antillón and colleagues^10^ estimated 17·8 million (95% CI 6·9–48·4) annual cases in low-income and middle-income countries. Our estimates differ only modestly from those that have been previously published, and our uncertainty intervals overlap with those of all previous estimates, except for the estimates adjusted for water-related risk from Mogasale and colleagues and from Kim and colleagues (appendix p 19). All previous death estimates fall within our uncertainty intervals.

Whereas global estimates show broad agreement, regional estimates are more variable (appendix p 19). Our model suggests that incidence is highest in south Asia, followed by southeast Asia, western sub-Saharan Africa, eastern sub-Saharan Africa, and Oceania, a distribution that is broadly similar to that estimated by Crump and colleagues.^6^ The estimates by Mogasale and colleagues^7^ and by Kim and colleagues^11^ suggest that incidence rates in central and eastern sub-Saharan Africa are similar to those of south Asia, but otherwise show a similar distribution to our estimates. Antillón and colleagues^10^ suggest a notably different distribution, estimating by far the highest incidence rates in Oceania, followed by central sub-Saharan Africa, with a relatively modest incidence in south Asia. These differences reflect the scarce data available about typhoid incidence, and the resulting sensitivity to analytical methods. Notably, Oceania and central sub-Saharan Africa are two potentially high-burden regions with almost no data, and they are the regions for which estimates vary most widely. Additionally, our estimates for Latin America and southern sub-Saharan Africa are lower than those published previously. Whereas other estimates were restricted to a small number of older studies in these regions, we believe that our ability to use more recent vital registration and reporting data has resulted in estimates that more accurately reflect the burden in these regions.

Several notable strengths underlie these estimates. We had access to previously unavailable data, and our estimates are therefore based on the largest collection of sources yet included in a typhoid fever burden assessment. Improved data access also allowed us to estimate case fatality by age and national income category, and to develop separate estimates for typhoid and paratyphoid fever. DisMod-MR is a powerful modelling tool that allowed us to use information from heterogeneous data and covariates, consequently allowing us to make more informed and detailed extrapolations over space, time, and age. Use of posterior simulation allowed us to propagate uncertainty through the modelling process. Finally, working within the larger GBD infrastructure allowed us to estimate YLLs, YLDs, and DALYs within the larger context of other causes of health loss, and thereby produce estimates that are adjusted for multiple causes of death and for multiple causes of non-fatal burden.

However, data about typhoid and paratyphoid fever are still relatively sparse and we have no data from much of sub-Saharan Africa, Oceania, and Latin America. The absence of data from Nigeria is a substantial source of uncertainty given the country's large population and potentially high burden. The absence of active surveillance data from high-burden countries is an important limitation. Moreover, the degree to which incidence from cohort studies represents incidence throughout a country is questionable, as many incidence studies were done in urban slum areas where typhoid fever burden can be greatest. This suggests a need for data covering both a larger geographical range and a greater diversity of sites within countries, which might help reduce the biases related to site selection. Similarly, data about case fatality are both sparse and of questionable quality. No gold standard data exist for typhoid fever case fatality: data from passive surveillance sources are likely to overestimate case fatality (ie, greater under-reporting of less-severe and non-fatal cases than of fatalities), data from active surveillance sources are likely to underestimate case fatality (because of early diagnosis and treatment), and hospital-based data contain conflicting sources of bias (over-representation of severe cases would tend to produce overestimates, whereas diagnosis and treatment would tend to produce underestimates). Our method for splitting the overall case fatality estimates into separate estimates for typhoid and paratyphoid fever is also limited by the sparse data. That said, our case fatality estimates, ranging from 0·3% to 1·6%, are in line with the widely held expert opinion that case fatality is approximately 1%,^5^, ^6^ and also in line with estimates produced by Mogasale and colleagues,^7^ which ranged from 0·4% to 2·1%. Beyond data limitations, our unsafe water covariate is an imperfect proxy for microbiologically safe water, which would probably be a better measure of faecal contamination and typhoid fever risk. Moreover, our model does not capture the burden associated with chronic carriage, most notably the increased risk of gallstones and gallbladder cancer.^32^ Finally, our modelling framework is restricted to predefined GBD age groups, which precludes estimation of burden for finer age categories that might be informative for vaccine policy.

Nevertheless, this study provides important information about the burden of typhoid and paratyphoid fevers and can be refined as additional data become available. Although SAGE has issued recommendations for typhoid conjugate vaccine use, decision making around vaccine introduction lies largely with individual countries. Most countries eligible to apply for support from Gavi, the Vaccine Alliance, are in sub-Saharan Africa, and these new estimates should complement in-country surveillance data and offer valuable evidence to help countries make informed decisions around vaccine introduction.

Our global estimates are largely consistent with previous typhoid fever burden estimates but add important information about the spatial distribution of the burden and the relative contributions of typhoid and paratyphoid fever; these findings offer empirical estimates of incidence and case fatality by age and income; and they provide important information about trends in incidence and mortality. The large differences in the published burden estimates for some regions stem from the paucity of data and highlight the need for more widespread active surveillance of enteric fever. As countries move forward with vaccine rollout, better data and better surveillance must be a priority. Despite notable progress, typhoid and paratyphoid fevers clearly remain a major cause of death and disability to which billions of people worldwide are continuously exposed.
