# Supplementary material for: The global burden of typhoid and paratyphoid fevers: a systematic analysis for the Global Burden of Disease Study 2017
Source: Lancet Infect Dis. 2019 Apr;19(4):369–81. doi: 10.1016/S1473-3099(18)30685-6 (PMC6437314; doi:10.1016/S1473-3099(18)30685-6)
Supplement: Supplementary appendix [file mmc1.pdf]

THE LANCET Infectious Diseases

Supplementary webappendix

This webappendix formed part of the original submission and has been peer reviewed. We post it as supplied by the authors.

Supplement to: GBD 2017 Typhoid and Paratyphoid Collaborators. The global burden of typhoid and paratyphoid fevers: a systematic analysis for the Global Burden of Disease Study 2017. *Lancet Infect Dis* 2019; published online Feb 18. http://dx.doi.org/10.1016/S1473-3099(18)30685-6.

Contents

A. Methods appendix 2
A1. GBD Locations 2
A2. Search strategy 3
A3. Overview of modelling process 4
A4. Incidence models 5
A5. Equations for splitting incidence between serovars 6
A6. Calculating YLDs 7
A7. Case fatality calculations 9
A8. Vital registration data 10
A9. Mortality modelling approaches 11
A10. Mortality calculations 11
A11. CoDCorrect 12
B. Supplemental results 14
C. Data sources 54
Incidence data 54
Vital registration data 59
Etiology proportion data 85
Case fatality data 90
D. References 94

A. Methods appendix

A1. GBD Locations

GBD uses a set of nested geographies for both analysis and reporting, with countries being nested inside of regions, regions being nested inside of super-regions, and super-regions being nested inside the world. Figure S1 below shows each region and super-region. Note that North Africa and Middle East super-region, and South Asia super-region, each contain only one region. All other super-regions contain between three and five regions. In total, there are seven super-regions and 21 regions.

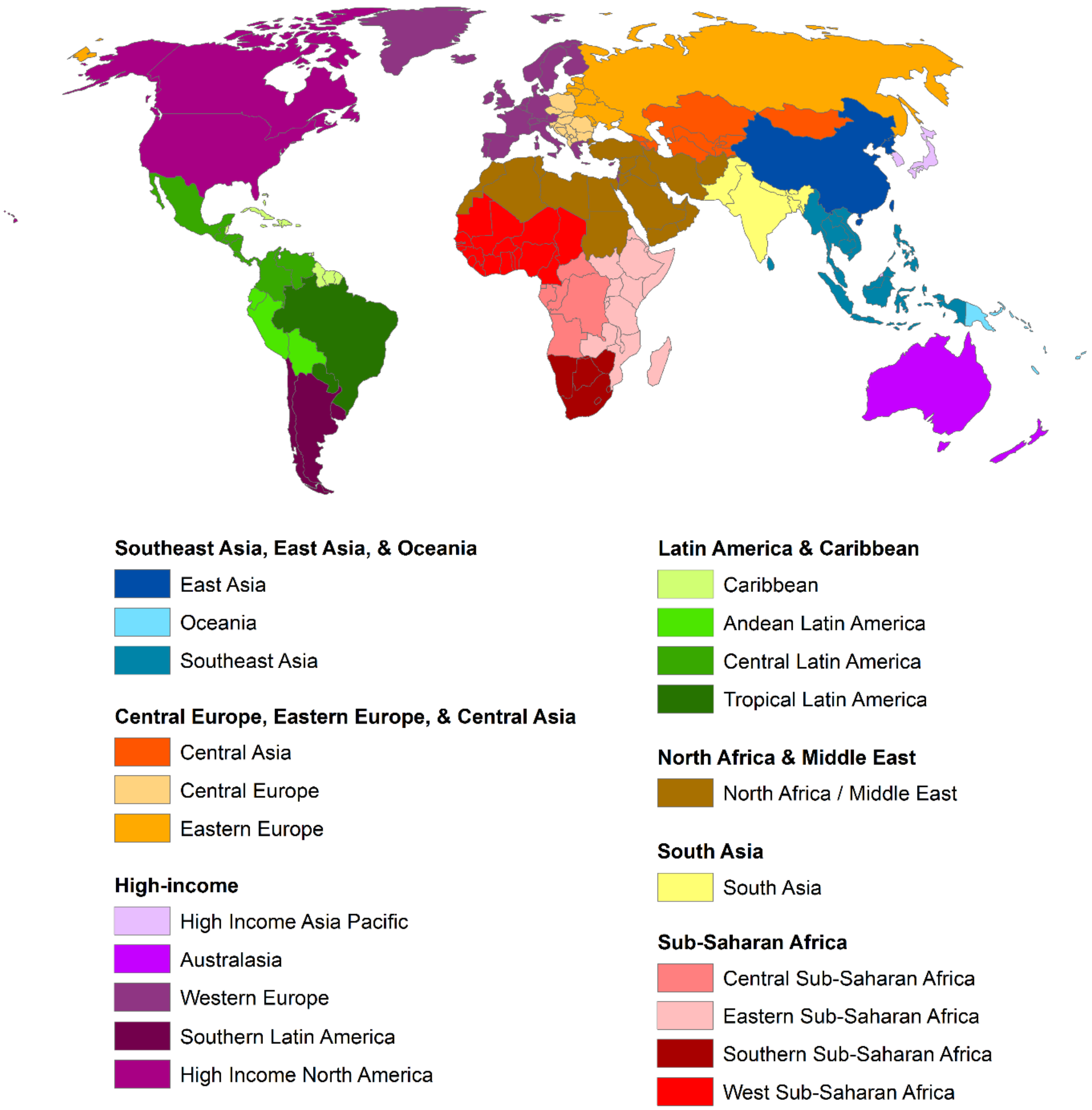

Figure S1: Global Burden of Disease regions and super-regions.

A2. Search strategy

Our data search strategy included three components: 1) data sources that were used in previous iterations of the GBD study, 2) a PubMed search of the published literature, and 3) reviewing citations from other published typhoid burden estimation studies.

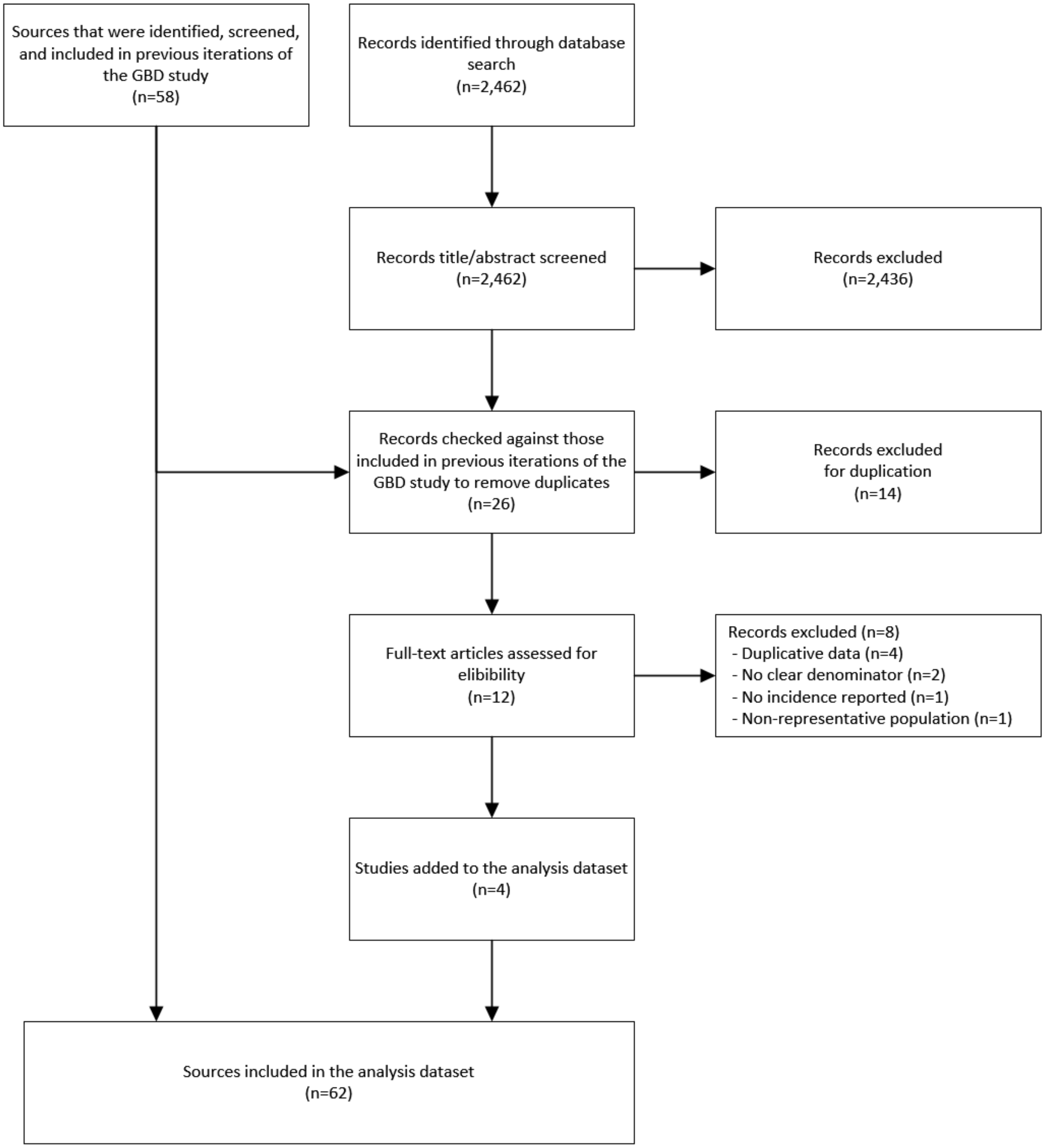

Figure S2: Flow diagram for systematic review of enteric fever incidence

A PubMed search conducted on 19 April 2017 with the term “("Paratyphoid Fever"[Mesh] OR "Typhoid Fever"[Mesh]) AND incidence” yielded 2,462 results. After title/abstract review, 26 sources were

identified as potentially having useful primary data. These 26 sources were crosschecked against those that were already in our database from previous iterations of the GBD study to avoid duplication: 14 articles were already in our database, had been used in previous iterations of the GBD study and were included in our analysis; we conducted a full-text screen on the remaining 12 articles. Of those 12 articles, after full-text screening, four were included and eight were excluded (four reported data that were duplicative of data from other included sources, two reported cases with no clear denominator, one reported no incidence, and one was not representative)(Figure S1). Finally, our review of the sources used in four previously published typhoid burden estimation studies[1–4] found no sources with data that were not otherwise captured (i.e. sources were either duplicates, or contained duplicative data).

A3. Overview of modelling process

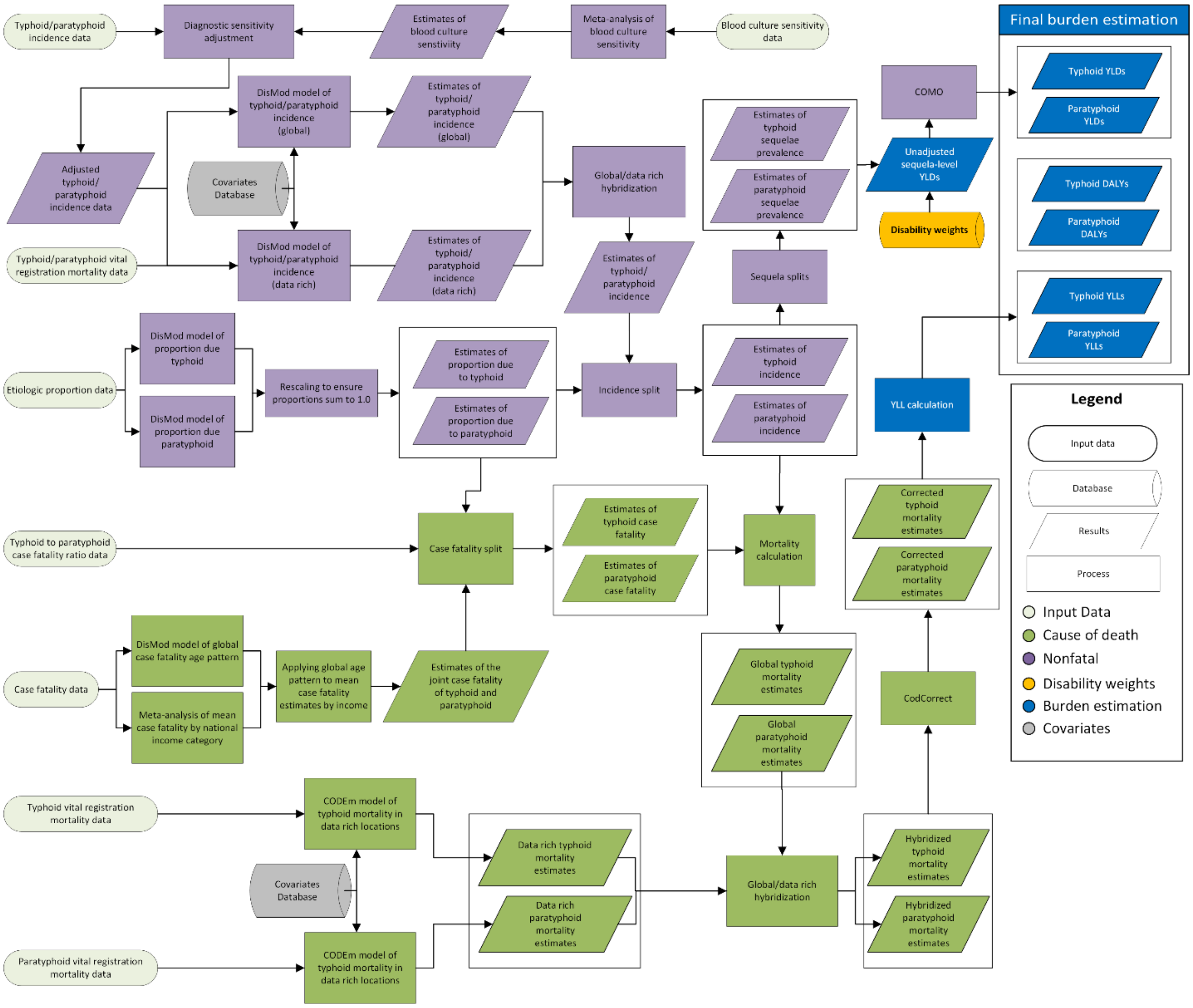

Figure S3: Flow diagram for typhoid and paratyphoid modelling process.

A4. Incidence models

The DisMod model

We modelled incidence using the Bayesian meta-regression tool DisMod-MR, which has been described elsewhere[5]. Briefly, DisMod-MR models incidence using a non-linear mixed effects model with an offset log-normal distribution, and produces estimates by age, sex, year, and location. It uses age-integration to accommodate data with disparate age categorization schemes. Country-level covariates may be used to help inform estimates where data are sparse. The tool uses a cascading geographic hierarchy in which all data are pooled to estimate a global fit, which then acts as a prior for each of the seven GBD super-regions. The model is refit within each super-region and the prior is modified based on any data within that super-region and the values of country-level covariates within that super-region. The fit in each super-region then becomes the prior for each region that falls within it. The model is again refit within each region, with each region's fit being derived from the prior, modified based on data and country-level covariates within the region. The cascade is then repeated for each country within each region. This cascade ensures that estimates have a strong spatial structure and allows extrapolations to countries with no data. In addition to country-level covariates, DisMod allows for study-level covariates that allow data to be adjusted to account for known sources of bias and heterogeneity.

Passive-to-active surveillance crosswalk

DisMod has the ability to adjust data points with known and identified sources of bias, a process we refer to as "crosswalking." To do this, we create a binary study-level covariate to identify each data point included in the model as either being in the reference category or the non-reference category. In this case, the reference category is active surveillance, and the non-reference category is passive surveillance. DisMod then empirically assesses the effect of the bias through regression, estimating a adjustment factor by which non-reference data must be multiplied to account for the given source of bias. DisMod then adjusts the non-reference data points, incorporating uncertainty in the adjustment factor with uncertainty from the original data. This adjustment factor for passive surveillance was 5·88 (3·44 – 7·14), meaning that incidence values from passive sources were inflated by a factor of 5·88 before being included in the final model.

Hybridization

Where a single DisMod model was used to produce incidence estimates for all locations, we found that the model fit was not universally acceptable, especially with regard to fitting the different age patterns observed in low and high burden settings. To solve this problem we used two separate DisMod models to estimate global incidence: one model was used to estimate incidence in super-regions with low burden (defined as those super-regions with age-standardized incidence rates <10 per 100,000, as estimated by the aforementioned global model); and a second model to estimate incidence in super-regions with moderate or high burden (defined as those super-regions with age-standardized incidence rates above 10 per 100,000). The results of these two models were then hybridized to create the final set of incidence estimates. Hybridization consisted of simply combining the incidence estimates from the two models such that the final set of estimates contained estimates from the high-burden model in high-burden super-regions, and estimates from the low-burden model in low-burden super-regions.

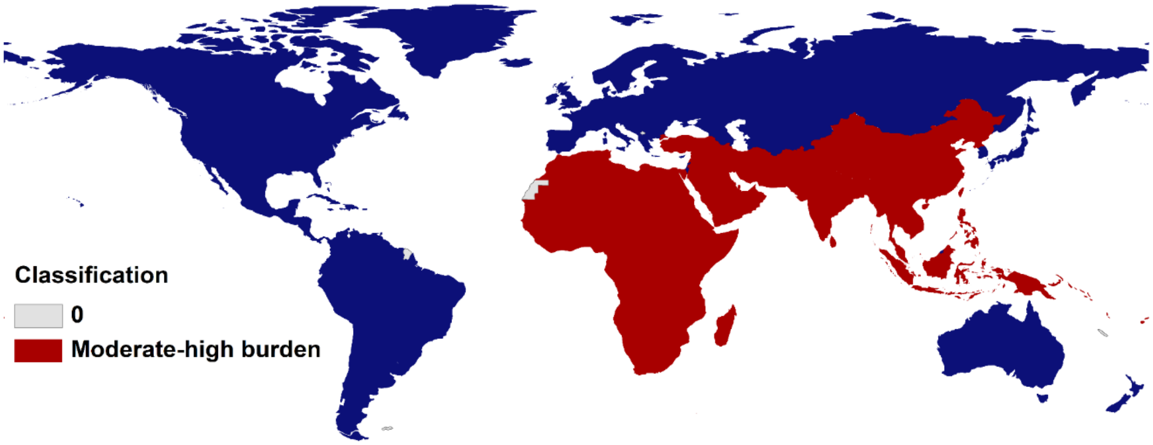

Figure S4: Map of low and moderate-high burden region classifications used in the hybridization scheme

A5. Equations for splitting incidence between serovars

Rescaling etiological proportions

We create two etiological proportion DisMod models: 1) the proportion of all cases due to typhoid, and 2) the proportion of all cases due to paratyphoid. While these models are based on the same data sources, they are independent models and, therefore, the proportions from the two models are not constrained to sum to 1·0. We impose this constraint, however, by rescaling the proportions from these two models to always sum to 1·0 for each draw and each age, sex, year and location. For each age-sex-year-location, and for each draw, the rescaled estimate of the proportion due to typhoid was calculated as unscaled estimate of the proportion due to typhoid divided by the sum of the unscaled estimates of the proportions for both typhoid and paratyphoid; the rescaled estimate for paratyphoid was calculated as unscaled estimate for paratyphoid divided by the sum of the unscaled estimates for both typhoid and paratyphoid.

$$P_{typh}' = \frac{P_{typh}}{P_{typh} + P_{para}}$$

$$P_{para}' = \frac{P_{para}}{P_{typh} + P_{para}}$$

Where P_{typh} is the model-based estimate of the proportion of cases due to typhoid, P_{para} is the model-based estimate of the proportion of cases due to paratyphoid, and P_{typh}' and P_{para}' are the rescaled proportions due to typhoid and paratyphoid, respectively.

Splitting incidence

We then calculate separate incidence estimates for typhoid and paratyphoid as the product of total incidence (*i.e.* the incidence of typhoid and paratyphoid combined), i_{total}, and the corresponding rescaled proportion derived above:

$$i_{typh} = i_{total} \times P_{typh}'$$

$$i_{para} = i_{total} \times P_{para}'$$

Where i_{typh} and i_{para} are the incidence rates of typhoid and paratyphoid, respectively.

A6. Calculating YLDs

Years lived with disability (YLDs) are calculated as the product of the prevalence of a sequela — calculated as the product of the incidence and duration of that sequela — and disability weight for the health state associated with that sequela:

$$YLDs = cases \times duration \times disability\ weight$$

All calculations were performed at the draw level to propagate uncertainty from incidence, duration and disability weights. Typhoid and paratyphoid cases were split proportionally between eight sequelae, four sequela each for typhoid and paratyphoid (Tables S1a and S1b). Each sequela was assigned a duration and the disability weight of the most closely matching health state (or states) from the 235 GBD health states[6] (Tables S2a and S2b).

Table S1a: Proportions used to split typhoid fever cases between sequelae.

Sequela	Proportion of cases
Moderate typhoid fever	0·35
Severe typhoid fever	0·43
Severe typhoid fever with gastrointestinal bleeding	0·05
Typhoid fever with abdominal complications (other than gastrointestinal bleeding)	0·17

Table S1b: Proportions used to split paratyphoid fever cases between sequelae.

Sequela	Proportion of cases
Mild paratyphoid fever	0·29
Moderate paratyphoid fever	0·52
Severe paratyphoid fever	0·14
Paratyphoid fever with abdominal complications	0·05

Table S2a: Sequelae of typhoid fever and their associated health states and durations

Sequela	Health State	Duration (years)	Disability weight
Moderate typhoid fever	"Infectious disease, acute episode, moderate"	0·038 (0·019-0·058)	0·051 (0·032-0·074)
Severe typhoid fever	"Infectious disease, acute episode, severe"	0·079 (0·038-0·134)	0·133 (0·088-0·19)
Severe typhoid fever with gastrointestinal bleeding	"Infectious disease, acute episode, severe"	0·071 (0·036-0·125)	0·133 (0·088-0·19)
	"Gastric bleeding"	0·005 (0·003-0·010)	0·325 (0·209-0·462)
Typhoid fever with abdominal complications other than	"Abdominopelvic problem, severe"	0·079 (0·038-0·134)	0·324 (0·22-0·442

Table S2b: Sequelae of paratyphoid fever and their associated health states and durations

Sequela	Health State	Duration (years)	Disability weight
Mild paratyphoid fever	"Infectious disease, acute episode, mild"	0·038 (0·019-0·058)	0·006 (0·002-0·012)
Moderate paratyphoid fever	"Infectious disease, acute episode, moderate"	0·038 (0·019-0·058)	0·051 (0·032-0·074)
Severe paratyphoid fever	"Infectious disease, acute episode, severe"	0·079 (0·038-0·134)	0·133 (0·088-0·19)
Paratyphoid fever with abdominal complications	"Abdominopelvic problem, moderate"	0·038 (0·019-0·058)	0·114 (0·078-0·159

When we apply these sequela splits, durations and disability weights we arrive at 1,578 YLD per 100,000 typhoid cases and 605 YLDs per 100,000 paratyphoid cases (Tables S3a and S3b).

Table S3a: Example YLD calculation for 100,000 hypothetical typhoid fever cases

Sequela	YLD Calculation	YLDs
Moderate typhoid fever	100000 * 0·35 * 0·038 * 0·051	67·8
Severe typhoid fever	100000 * 0·43 * 0·079 * 0·133	451·8
Severe typhoid fever with gastrointestinal bleeding	100000 * 0·05 * (0·071 * 0·133 + 0·005 * 0·325)	55·3
Typhoid fever with abdominal complications (other than gastrointestinal bleeding)	100000 * 0·17 * 0·079 * 0·324	435·1
Total		1010·1

Table S3b: Example YLD calculation for 100,000 hypothetical paratyphoid fever cases

Sequela	YLD Calculation	YLDs
Mild paratyphoid fever	100000 * 0·29 * 0·038 * 0·006	6·6
Moderate paratyphoid fever	100000 * 0·52 * 0·038 * 0·051	100·8
Severe paratyphoid fever	100000 * 0·14 * 0·079 * 0·133	147·1
Paratyphoid fever with abdominal complications	100000 * 0·05 * 0·038 * 0·114	21·7
Total		276·1

A7. Case fatality calculations

We used a DisMod model to estimate the age-pattern for case fatality but, since we had insufficient data to run a full DisMod model, we did not use the DisMod model to estimate the overall magnitude of case fatality. Instead, we converted DisMod's age-specific case fatality estimates to age-specific relative risks (RR), where each age-specific RR was the risk of a case resulting in death relative to the mean age-specific case fatality for all ages, $\overline{cf'}$. The mean age-specific case-fatality depends on the age-structure of cases (*e.g.* assuming higher case fatalities among those of younger ages, a case distribution centered among children would yield a higher mean age-specific case fatality than would a case distribution centered among adults). Since we were estimating case fatality by national income category, and since the age distribution of cases differs between these income categories, we estimated $\overline{cf'}$ and age-specific RRs separately for each income category. For each income category, *i*, we, calculated the mean age-specific case fatality, $\overline{cf_i'}$, as the case-weighted mean of age-specific case fatalities:

$$\overline{cf_i'} = \frac{\sum_{a=1}^{n} cf_{ai}' \times cases_{ai}}{\sum_{a=1}^{n} cases_{ai}}$$

Where cf_{ai}' is the DisMod case fatality estimate in age category *a* and income category *i*, and $cases_{ai}$ is the number of typhoid/paratyphoid cases in age category *a* and income category *i* (as estimated from the DisMod incidence model described in the main Methods section).

For each income category, we converted age-specific case fatality estimates to age-specific RRs:

$$RR_{ai} = \frac{cf_{ai}'}{\overline{cf_i'}}$$

We estimated overall case fatality in each national income category, cf_i, through a meta-analysis, using the "metaprop" function[7] in Stata 13 (StataCorp, College Station, Texas), and imposed the age-structures derived from the DisMod case fatality model. The final age- and income-specific case fatality estimates, cf_{ai}, were calculated as,

$$cf_{ai} = cf_i \times RR_{ai}$$

Here, cf_{ai}, is the age- and income-specific case fatality estimate for typhoid and paratyphoid combined and, for simplicity, may now be referred to as cf_{total}. We treated cf_{total} as a weighted average of the case fatalities for typhoid (cf_{typh}) and paratyphoid (cf_{para}):

$$cf_{total} = (pr_{typh} \times cf_{typh}) + (pr_{para} \times cf_{para})$$

Where pr_{typh} and pr_{para} are the proportion of all cases due to typhoid and paratyphoid, respectively. Using data from China CDC,[8] we estimated the RR of a case resulting in death comparing typhoid to paratyphoid, $RR_{cf_t:cf_p}$, as 1·89 (95% CI 1·09 – 3·28):

$$RR_{cf_t:cf_p} = \frac{cf_{typh}}{cf_{para}} = 1.89$$

$$cf_{typh} = 1.89 \times cf_{para}$$

$$cf_{total} = (1.89 \times pr_{para} \times cf_{typh}) + (pr_{para} \times cf_{para})$$

$$cf_{para} = \frac{cf_{total}}{1.89 \times pr_{typh} + pr_{para}}$$

A8. Vital registration data

For locations with four and five star rated CoD data, we derived incidence and etiological proportion data from vital registration (VR) death data. Before being used, several adjustments are applied to CoD data to correct for unambiguously erroneous coding (e.g. prostate cancer deaths among women), vague codes, and absolute numbers of deaths are adjusted to account for incomplete death reporting. These correction and redistribution processes are detailed elsewhere.[9] For each location, 5-year estimation period, GBD age group, and sex, we calculated the total number of typhoid or paratyphoid deaths as the sum of all such deaths in the final CoD database. We derive incidence, *i*, from deaths by dividing deaths by case fatality, *cf*, and population:

$$i_{typh} = \frac{deaths_{typh}}{population \cdot cf_{typh}}$$

$$i_{para} = \frac{deaths_{para}}{population \cdot cf_{para}}$$

We then derive etiologic proportion, *Pr*, data:

$$Pr_{typh} = \frac{i_{typh}}{i_{typh} + i_{para}}$$

$$Pr_{para} = \frac{i_{para}}{i_{typh} + i_{para}}$$

All calculations are done at the draw-level, using 1,000 draws to propagate uncertainty in inputs into the derived data points. We derive the point estimate from the mean of the draws, and the lower and upper bounds of the 95% uncertainty interval from the 2.5th and 97.5th percentiles of the draws.

A9. Mortality modelling approaches

For locations with CoD data rated 4 stars or higher, we estimated mortality directly from vital registration data using the CoD ensemble model (CODEm) tool.[9] For other locations we estimated mortality using a natural history approach. Figure S5 shows the modelling approach used for each location: locations for which mortality was estimated directly using CODEm are shown in blue, and those for which mortality was estimated using a natural history model are shown in red.

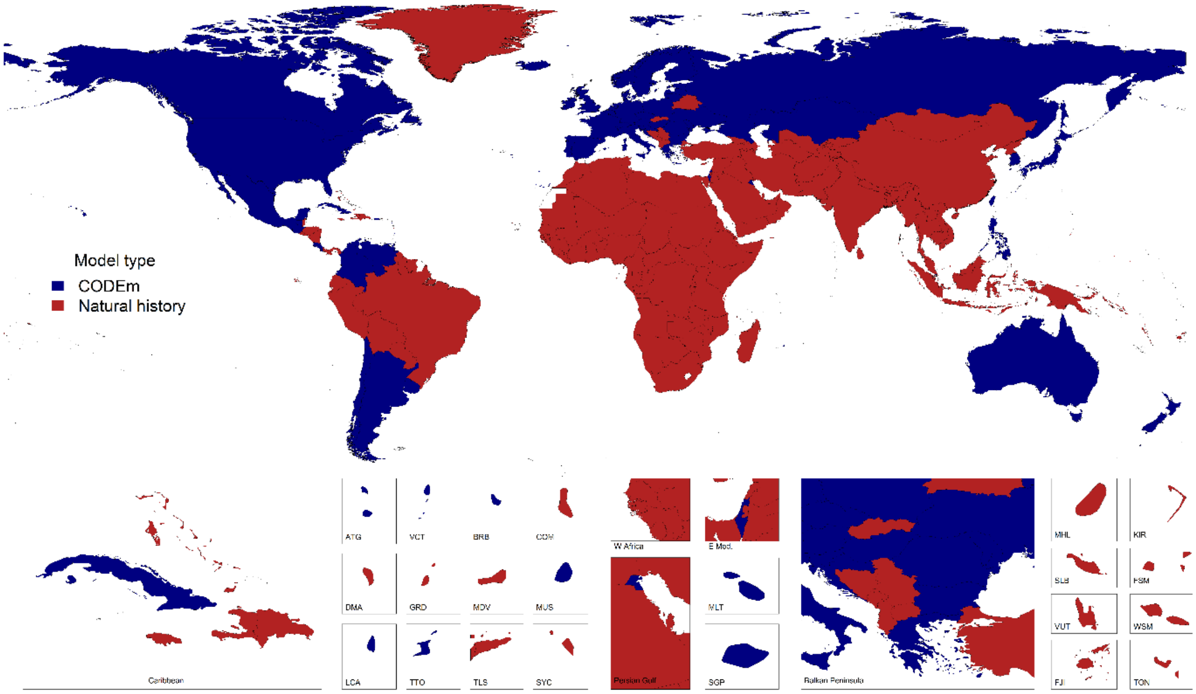

Figure S5: Map of mortality modelling approach by country

A10. Mortality calculations

For locations where we estimate mortality using a natural history approach, we calculate mortality rates, MR, as the product of incidence and case fatality:

$$MR_{typh} = i_{typh} \times cf_{typh}$$

$$MR_{para} = i_{para} \times cf_{para}$$

A11. CoDCorrect

To ensure consistency the GBD Study imposes two constraints in estimating cause-specific mortality: 1) that each death has a single underlying cause, and 2) that the sum all cause-specific mortality estimates must equal the estimated all-cause mortality within every location, year, sex, and age group. We impose these constraints through a hierarchical rescaling process called CoDCorrect, which has been described in detail previously.[9] In general, where data are sparser (i.e. where we have less data on cause-specific mortality), our mortality estimates are less certain, aggregated cause-specific estimates are likely to deviate more greatly from the all-cause mortality estimate, and the effect of CoDCorrect is likely to be more pronounced. Note that data sparsity here, refers not just to data on typhoid, but to data on all causes of death. Conversely, where we have nearly complete vital registration data (i.e. where the underlying cause is known for nearly every death), little uncertainty exists in our mortality estimates, aggregated cause-specific estimates are unlikely to notably deviate from the all-cause mortality estimate, and the effect of CoDCorrect will be negligible.

A) **B)**

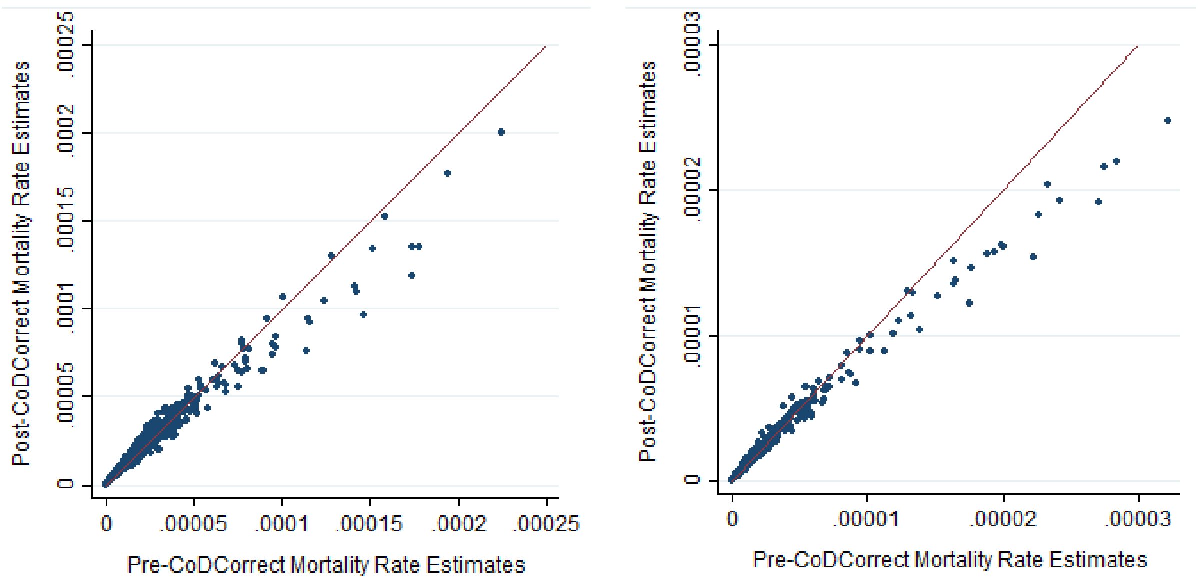

Figure S6: Comparison of typhoid (A) and paratyphoid (B) mortality rate estimates before and after CoDCorrect for each country, and year. The red line shows equality (i.e. points that fall along this line represent country-years for which mortality estimates were unchanged by the CodCorrect process).

Table S4: Comparison of global estimates of typhoid and paratyphoid deaths before and after CoDCorrect, by year

Year	Pre-CoDCorrect	Post-CoDCorrect	Absolute Change	Percent Change
		Typhoid		
1990	249,938	202,039	-47,899	-19.20%
1995	223,640	185,445	-38,195	-17.10%
2000	202,194	171,566	-30,629	-15.10%
2005	183,929	160,197	-23,733	-12.90%
2010	163,202	145,424	-17,778	-10.90%
2017	127,078	116,815	-10,263	-8.10%
		Paratyphoid		
1990	35,926	28,506	-7,420	-20.70%
1995	33,570	27,302	-6,267	-18.70%
2000	30,346	25,241	-5,105	-16.80%
2005	27,299	23,018	-4,281	-15.70%
2010	24,213	20,881	-3,332	-13.80%
2017	21,672	19,108	-2,564	-11.80%
		Typhoid + Paratyphoid		
1990	285,864	230,545	-55,319	-19.40%
1995	257,210	212,747	-44,462	-17.30%
2000	232,540	196,806	-35,734	-15.40%
2005	211,228	183,214	-28,014	-13.30%
2010	187,415	166,305	-21,110	-11.30%
2017	148,750	135,922	-12,828	-8.60%

B. Supplemental results

A)

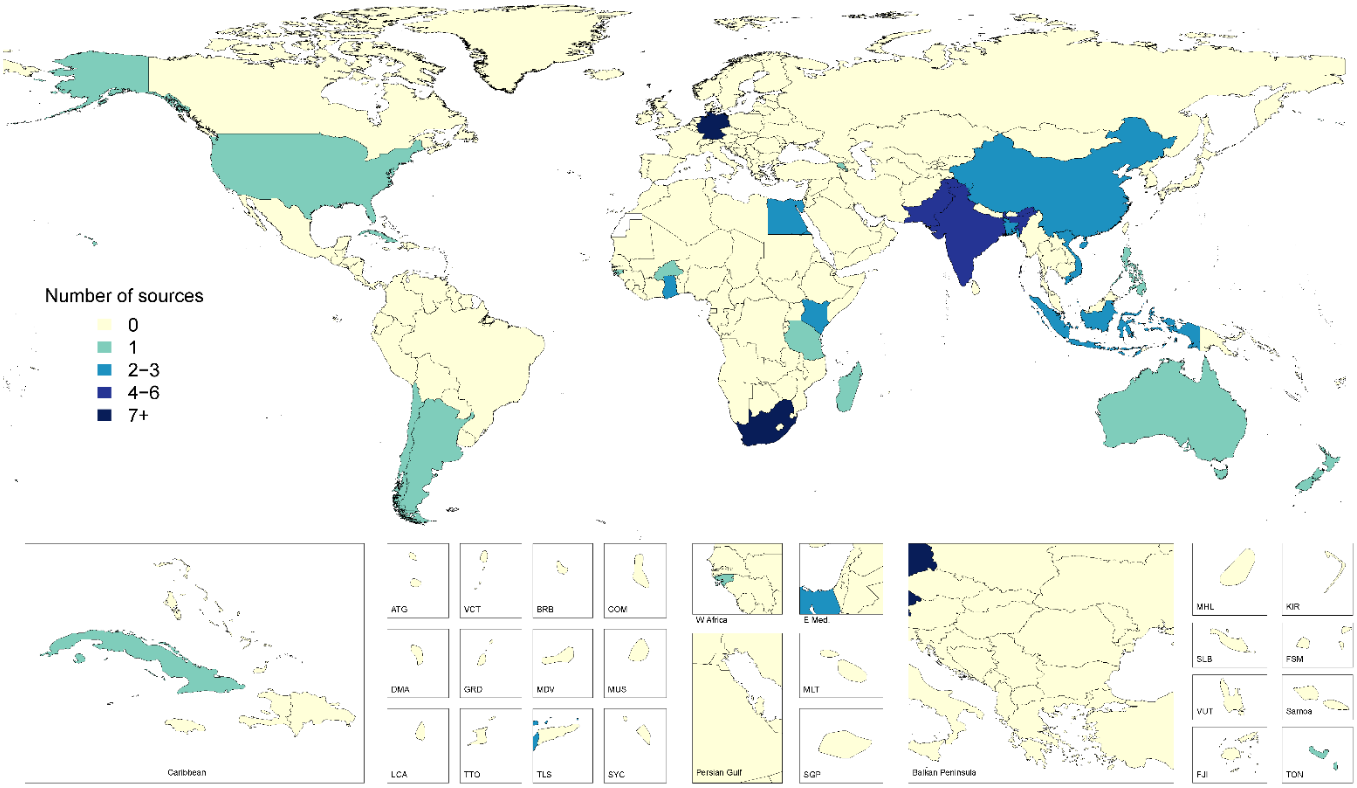

B)

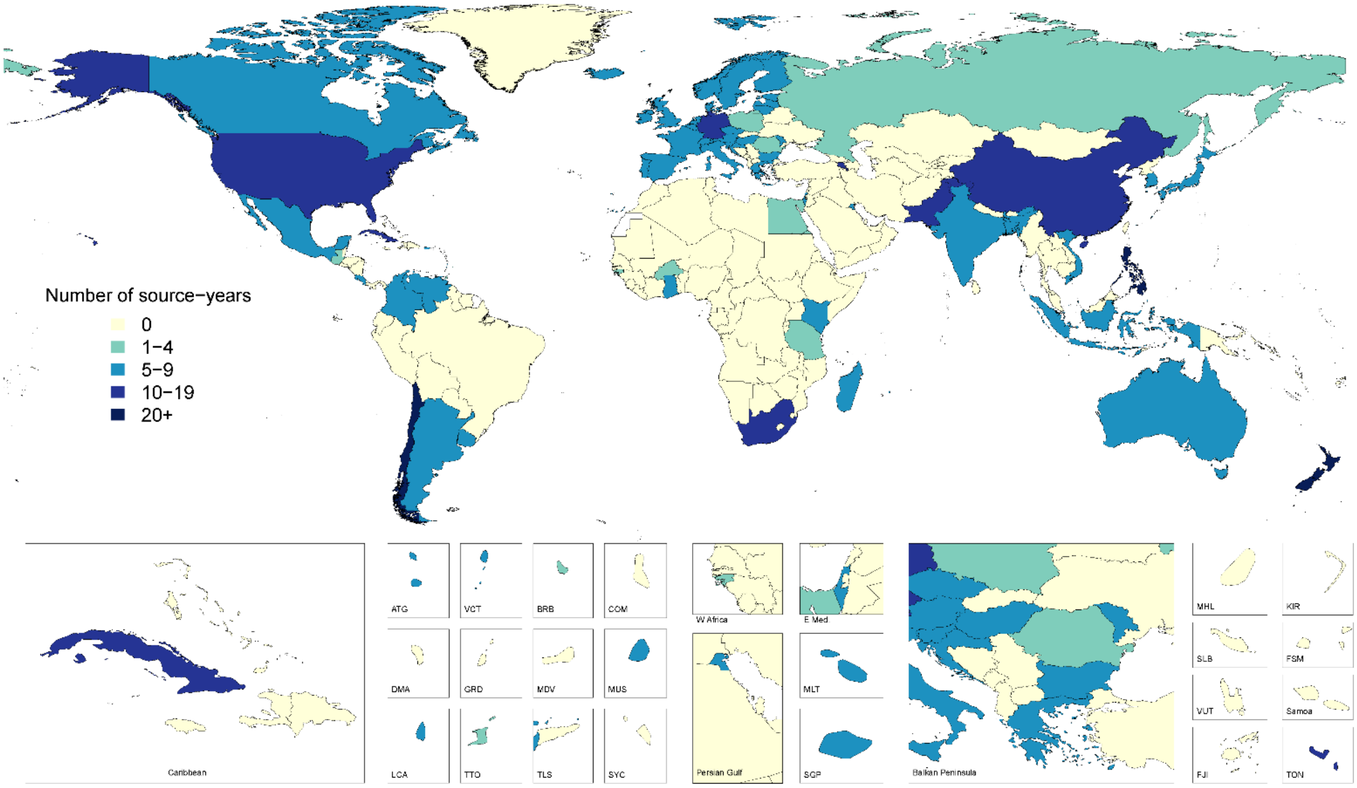

Figure S7: The number of incidence data sources, by country, excluding vital registration sources (A), and the number of source-years, by country, including vital registration sources (B).

Source-years are calculated for each location as the sum of the number of years of observation across all sources.

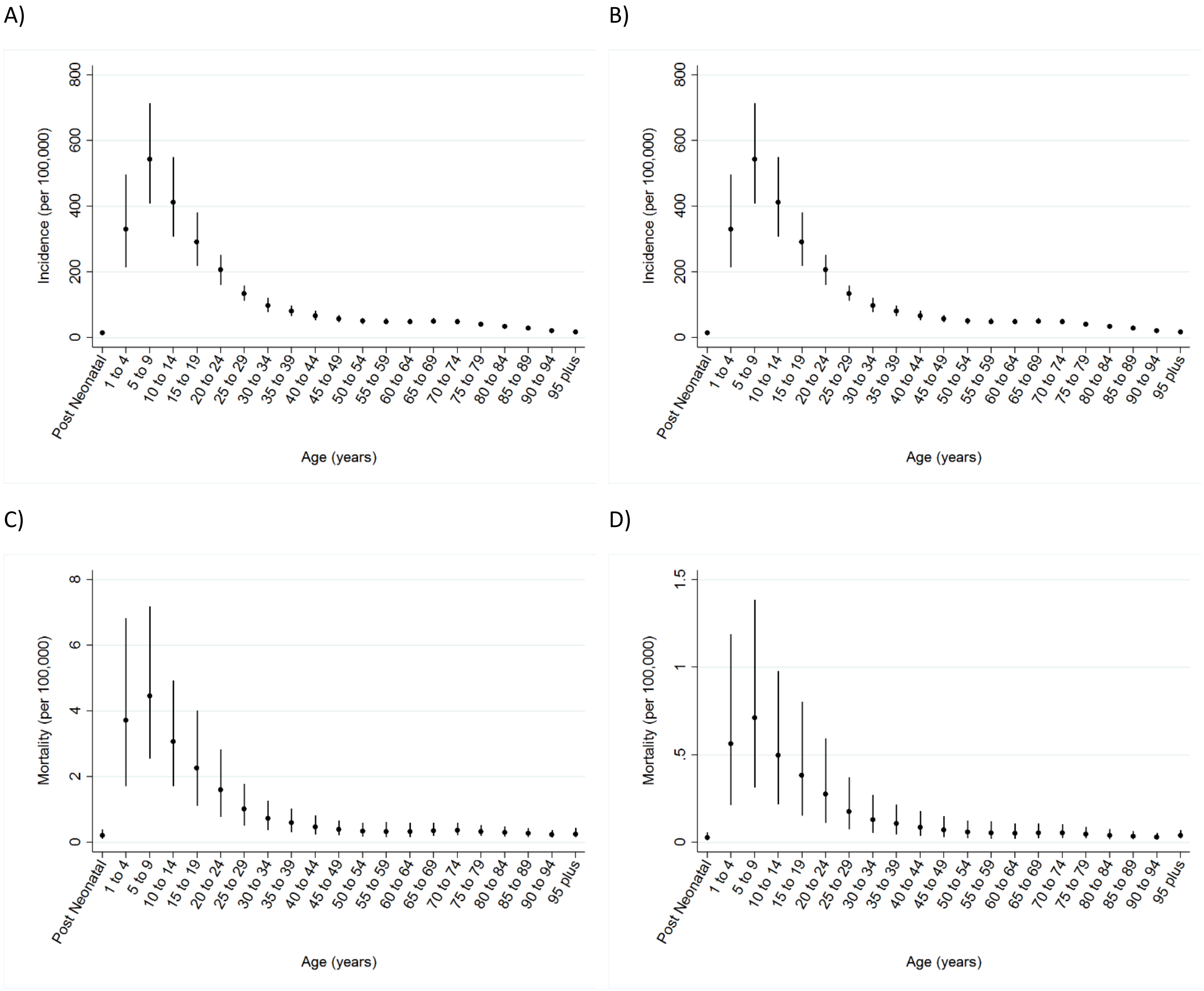

Figure S8: Incidence rates (per 100,000) of typhoid (A) and paratyphoid (B), by age; and mortality rates (per 100,000) for typhoid (C) and paratyphoid (D), by age, in 2017

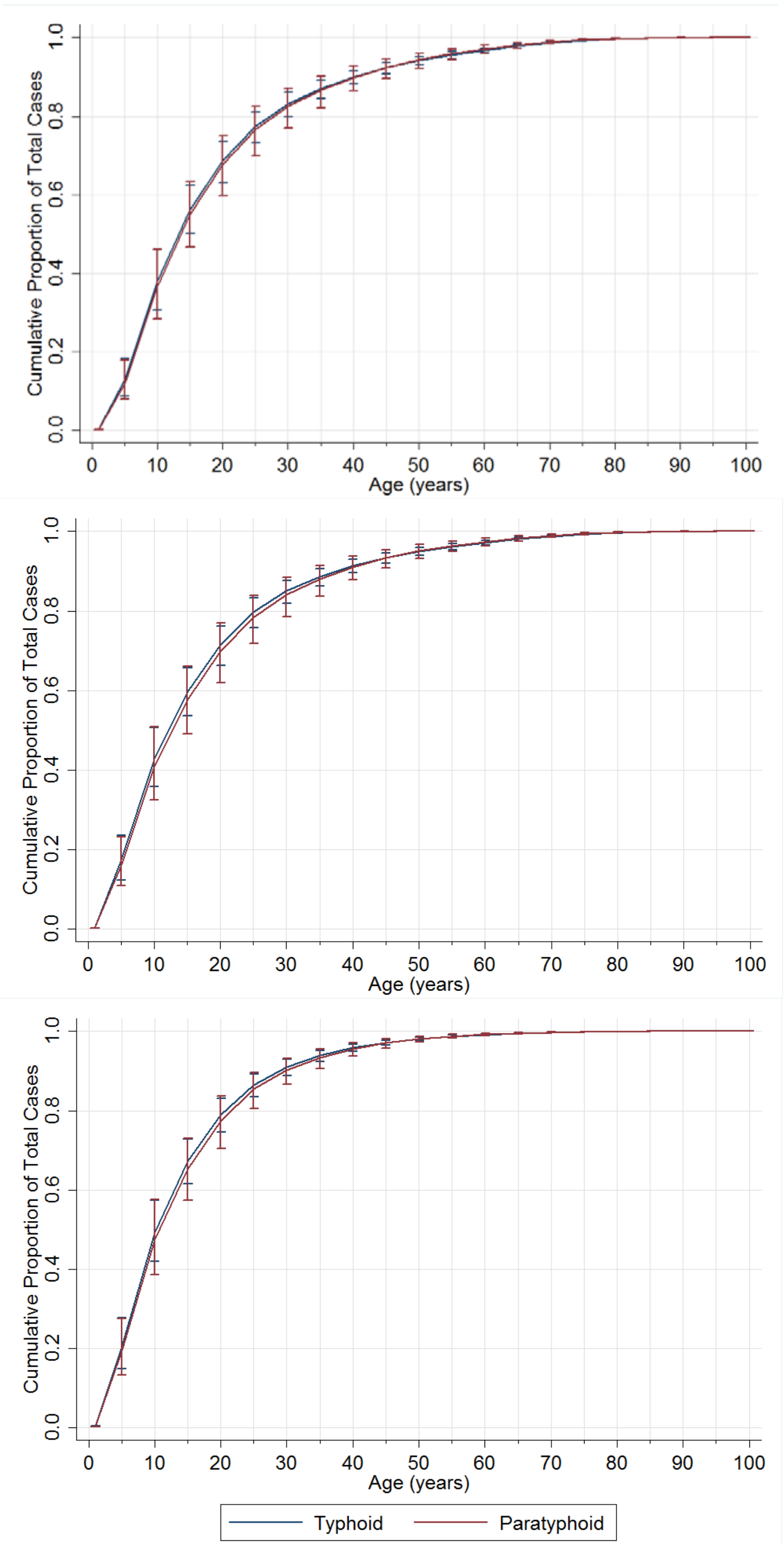

Figure S9: The cumulative proportion of total cases (top), deaths (middle), and DALYs (bottom), by age, for typhoid and paratyphoid, in 2017

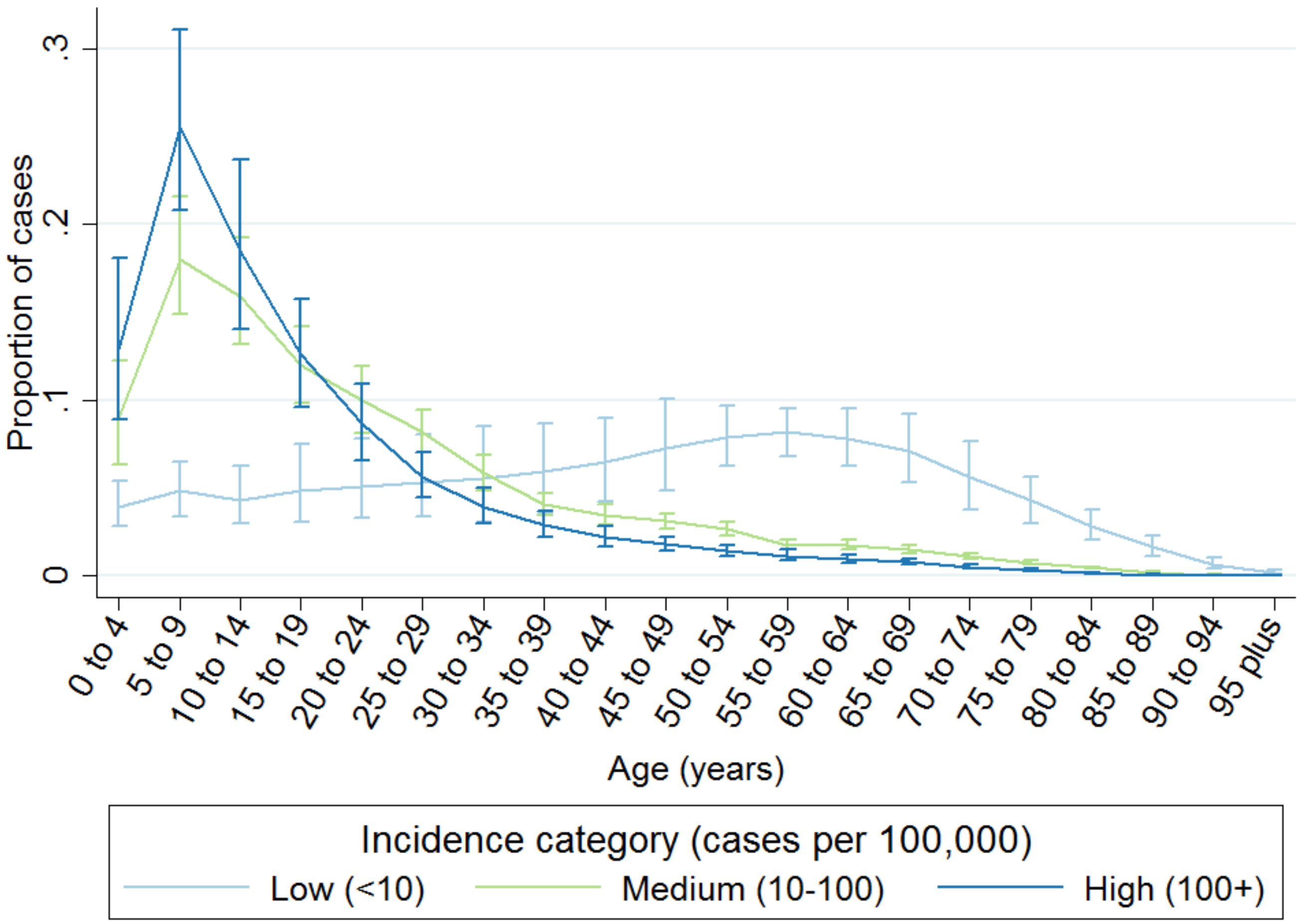

Figure S10: Age patterns of typhoid and paratyphoid by incidence category

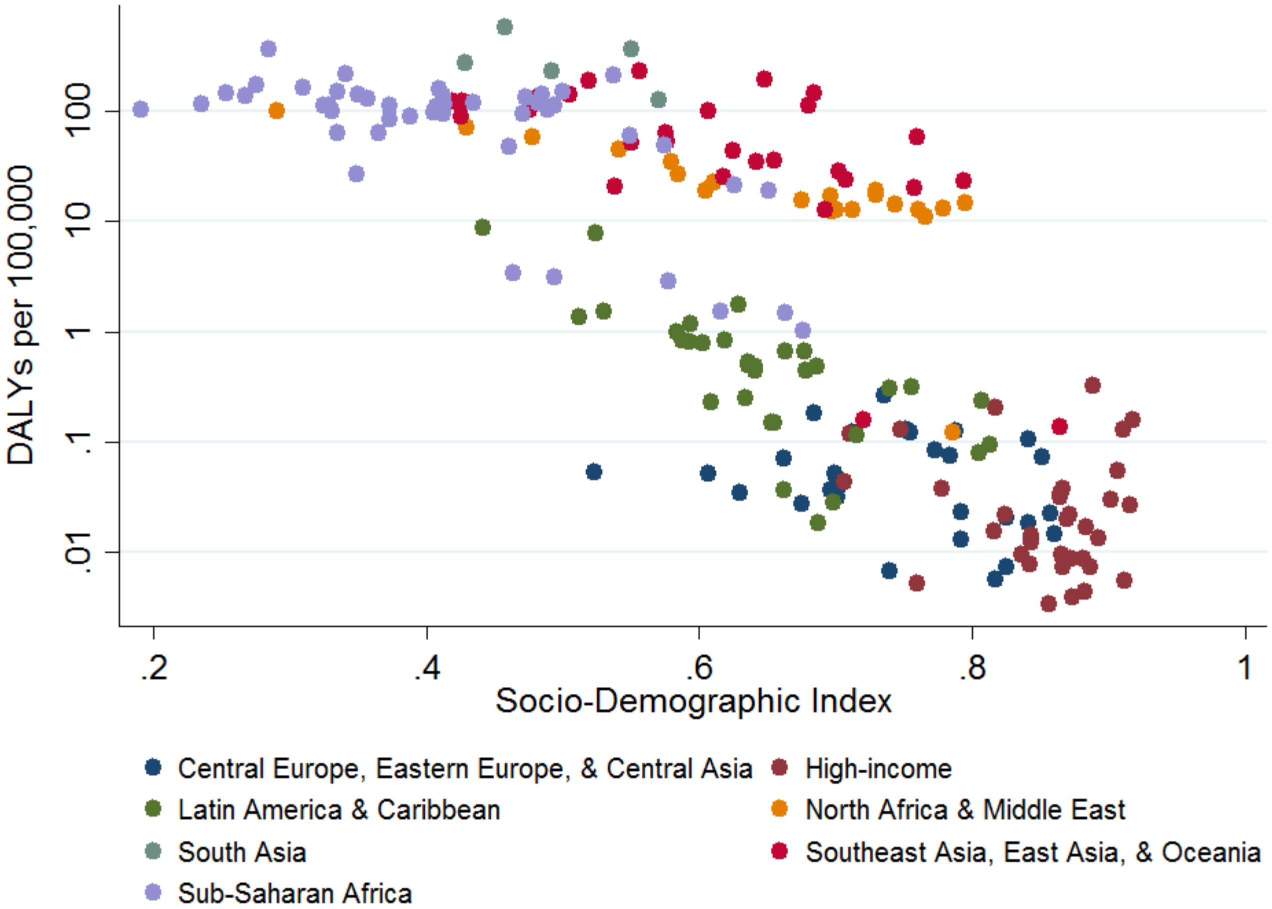

Figure S11: Age-standardized DALY rates (per 100,000) by socio-demographic index (SDI) and super-region, in 2017.

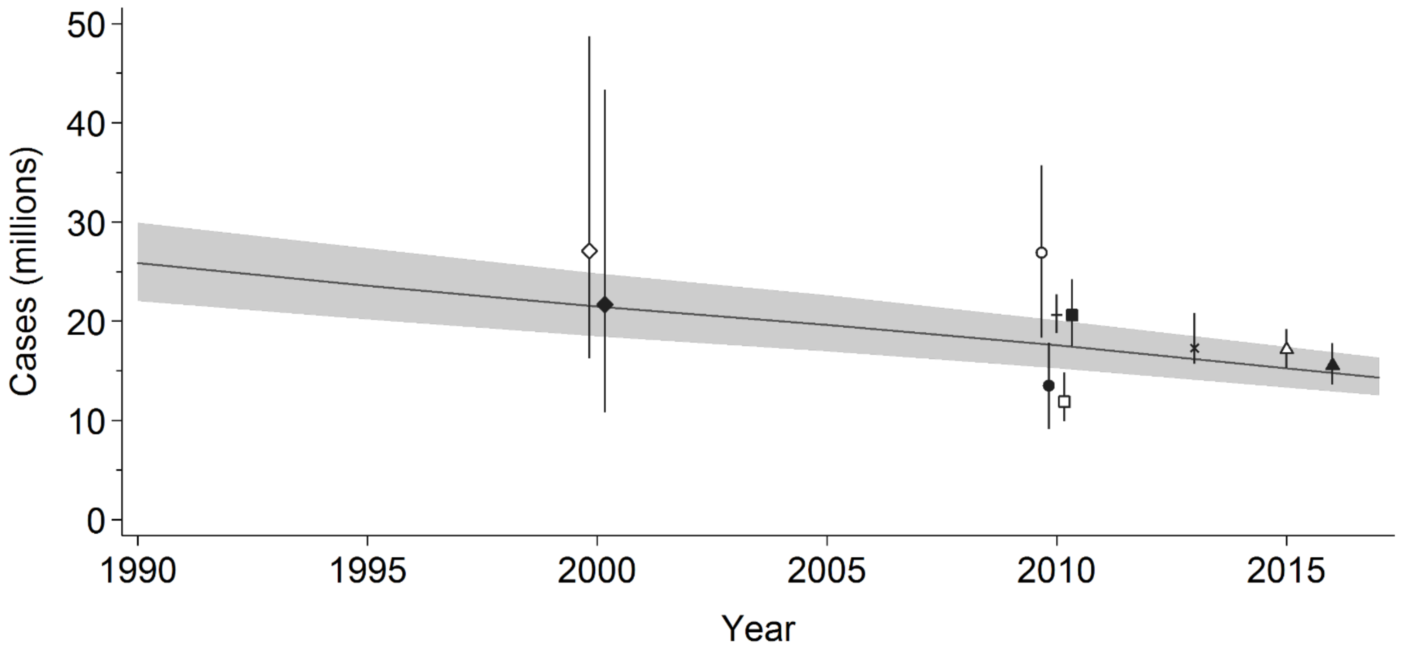

Figure S12: Comparison of previous case estimates for typhoid and paratyphoid against the GBD 2017 global trend estimates. The trend line and shaded area represent our estimates and 95% uncertainty interval for the global number of typhoid and paratyphoid cases, by year. The solid and hollow diamonds represent the Crump et al[2] estimates for typhoid only and typhoid plus paratyphoid, respectively. The solid and hollow circles represent the Buckle et al[3] estimates for cases before and after adjustment for diagnostic sensitivity, respectively. The plus, "x", hollow triangle, and solid triangle represent the estimate from GBD 2010[10], GBD 2013[11], and GBD 2015[12], and GBD 2016[13] respectively. The solid and hollow squares represent the estimates from Mogasale et al[14] before and after adjusting for water-related risk, respectively. Points for the years 2000 and 2010 have been offset on the x-axis to prevent overlap. Note that estimates from Antillón et al.[1] and Kim et al.[15] are not shown, since the absence of a specified estimation year precluded their placement on the x-axis.

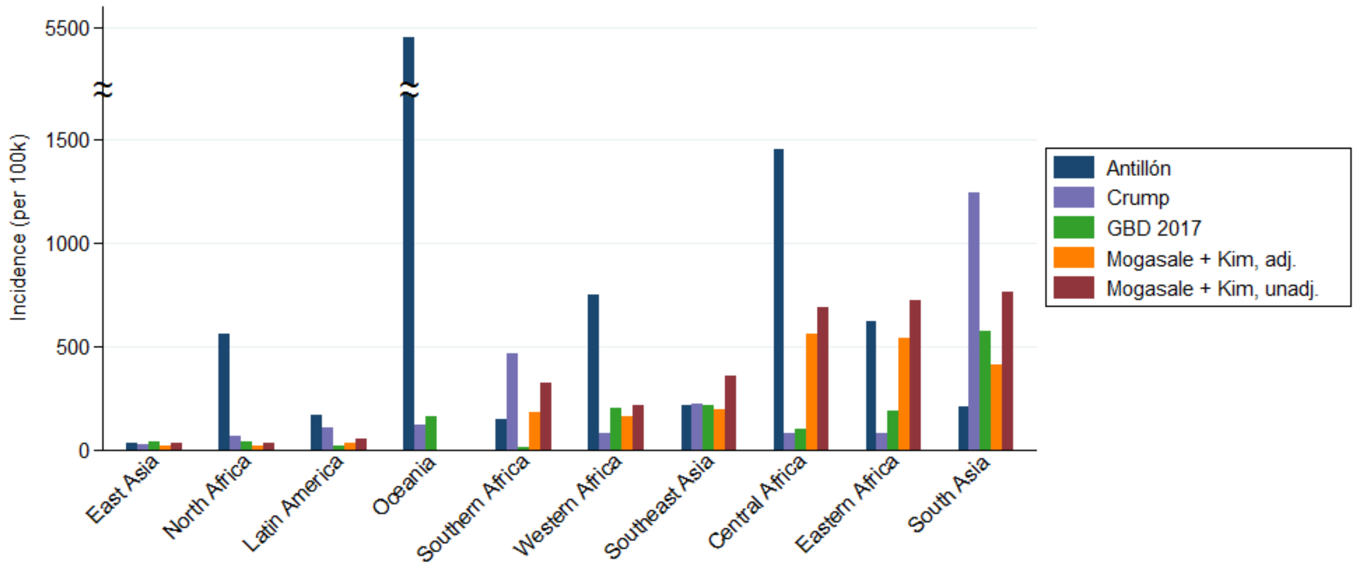

Figure S13: Comparison of regional estimates of typhoid incidence from this study (GBD 2017), Antillón et al[1], Crump et al.[2], and estimates from Mogasale et al.[14] combined with updated estimates for sub-Saharan Africa by Kim et al.[15], including estimates that were adjusted for water-related risk, and those that were unadjusted. Note the y-axis break.

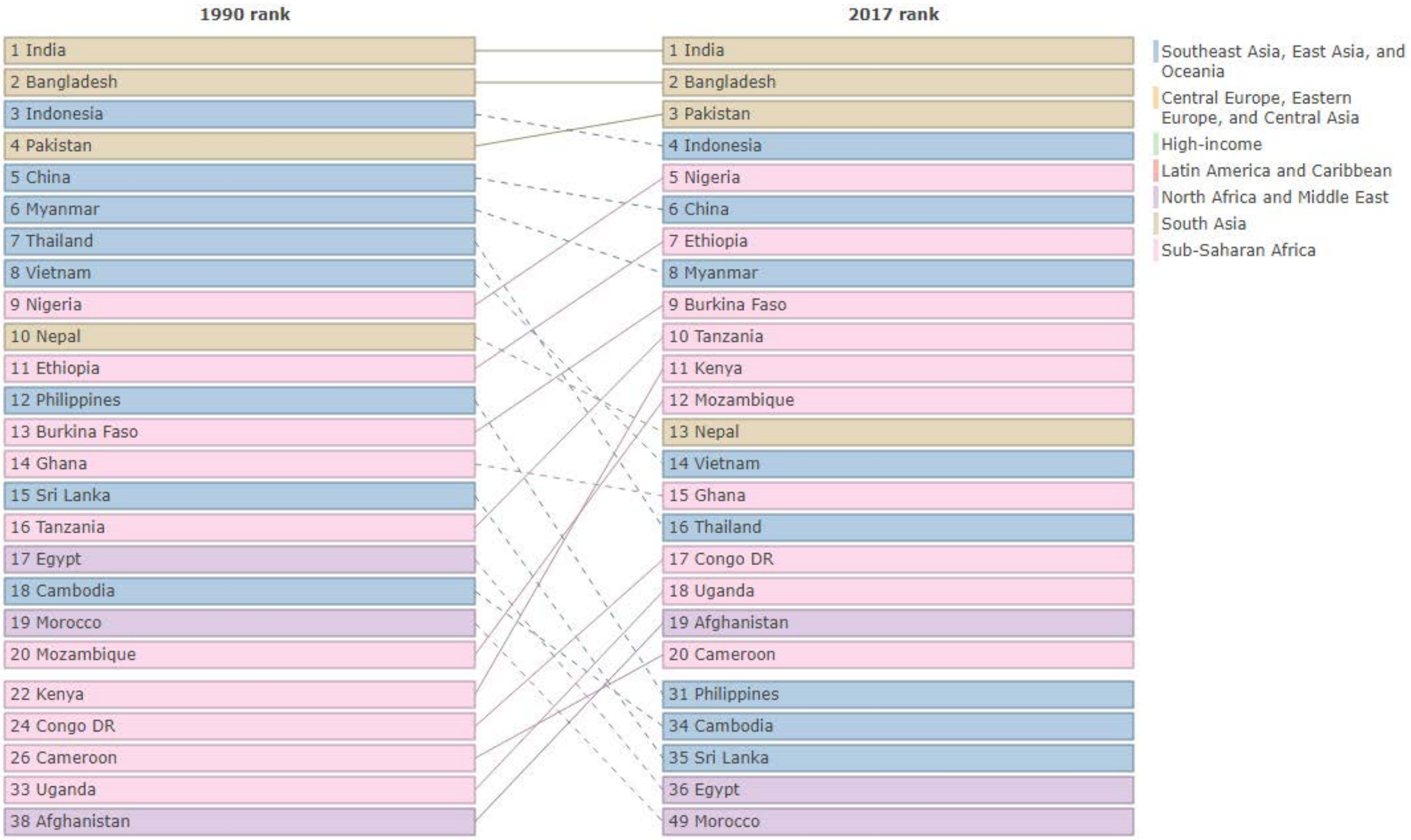

Figure S14: Countries ranked by absolute number of all-age DALYs in 1990 and 2017, showing the 20 countries with the largest number of DALYs in either 1990 or 2017. Colors indicate the region in which a given country falls.

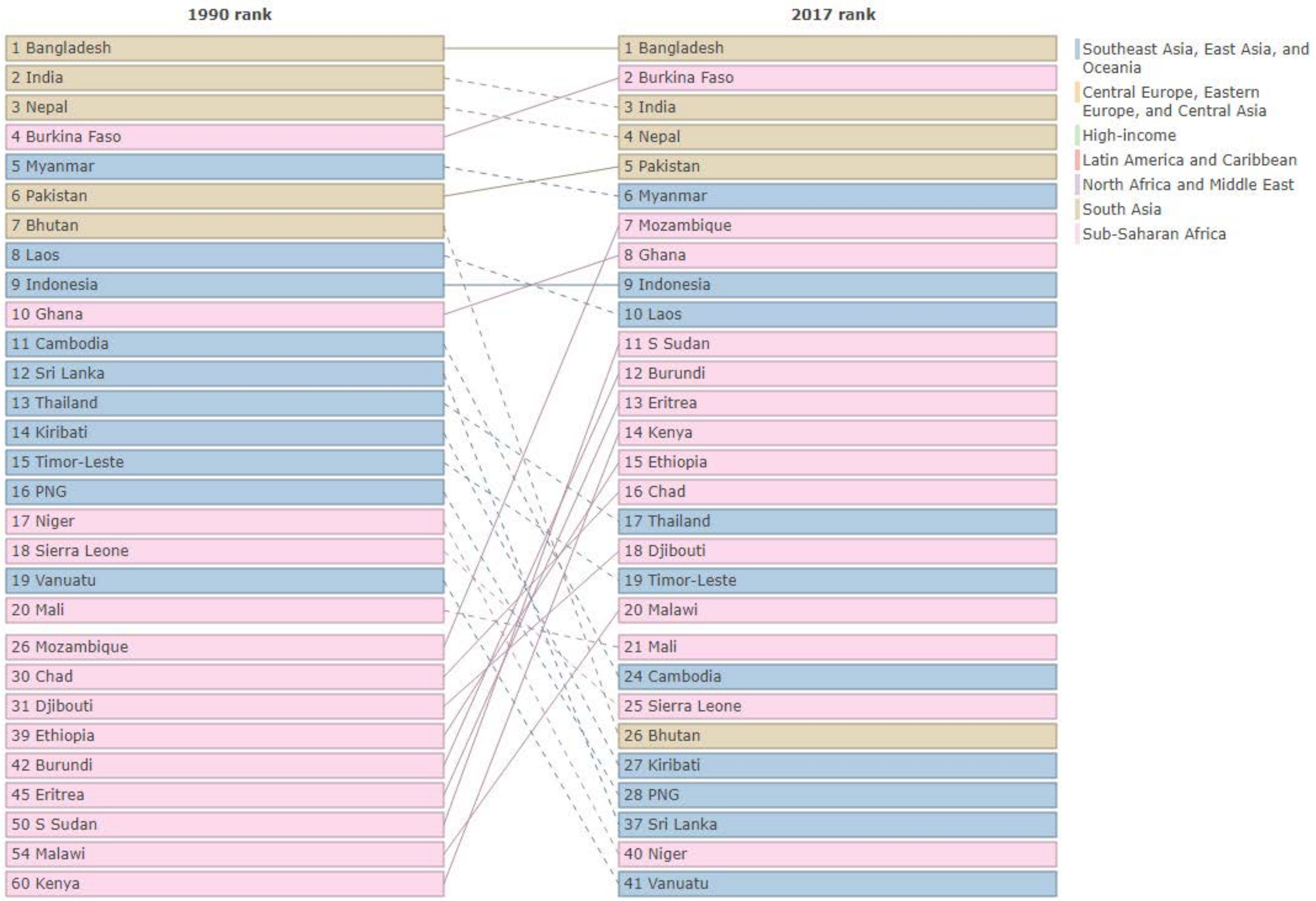

Figure S15: Countries ranked by age-standardized DALY rates in 1990 and 2017, showing the 20 countries with the highest DALY rates in either 1990 or 2017. Colors indicate the region in which a given country falls.

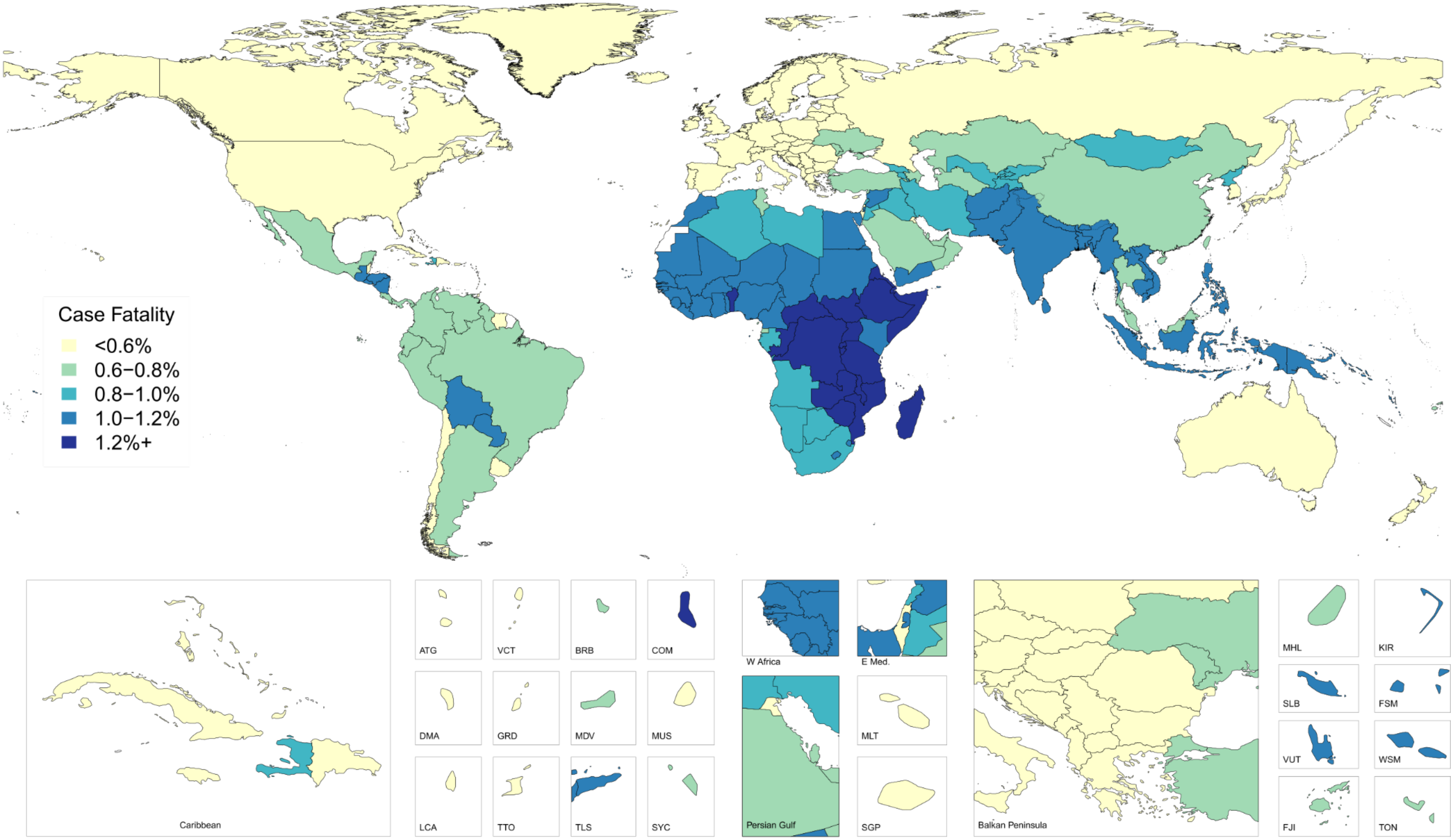

Figure S16: Estimated all-age case fatality, by country for typhoid and paratyphoid combined, in 2017

Table S5: Mean all-age case fatality by region

Region	Case fatality (95% UI), %
East Asia	0.67 (0.32, 1.20)
Southeast Asia	1.07 (0.58, 1.83)
Oceania	1.07 (0.57, 1.82)
Central Asia	0.81 (0.43, 1.37)
Central Europe	0.39 (0.19, 0.76)
Eastern Europe	0.57 (0.26, 1.14)
High-income Asia Pacific	0.41 (0.13, 0.95)
Australasia	0.39 (0.12, 0.93)
Western Europe	0.40 (0.13, 0.96)
Southern Latin America	0.47 (0.17, 1.03)
High-income North America	0.39 (0.13, 0.93)
Caribbean	0.77 (0.39, 1.33)
Andean Latin America	0.77 (0.40, 1.34)
Central Latin America	0.76 (0.40, 1.31)
Tropical Latin America	0.70 (0.35, 1.26)
North Africa and Middle East	1.02 (0.54, 1.75)
South Asia	1.06 (0.57, 1.80)
Central Sub-Saharan Africa	1.15 (0.62, 1.96)
Eastern Sub-Saharan Africa	1.23 (0.66, 2.10)
Southern Sub-Saharan Africa	0.98 (0.52, 1.70)
Western Sub-Saharan Africa	1.11 (0.59, 1.91)
East Asia	0.67 (0.32, 1.20)

Table S6: Global estimates of case fatality by age group

Age group	Case fatality (95% UI), %
Post Neonatal	1.45 (0.71, 2.67)
1 to 4	1.36 (0.73, 2.34)
5 to 9	1.22 (0.66, 2.07)
10 to 14	1.06 (0.57, 1.81)
15 to 19	0.93 (0.48, 1.63)
20 to 24	0.87 (0.44, 1.54)
25 to 29	0.86 (0.45, 1.48)
30 to 34	0.85 (0.45, 1.44)
35 to 39	0.84 (0.46, 1.39)
40 to 44	0.81 (0.45, 1.34)
45 to 49	0.78 (0.43, 1.32)
50 to 54	0.77 (0.41, 1.32)
55 to 59	0.76 (0.38, 1.38)
60 to 64	0.77 (0.39, 1.38)
65 to 69	0.81 (0.46, 1.31)
70 to 74	0.84 (0.50, 1.33)
75 to 79	0.89 (0.53, 1.40)
80 to 84	0.93 (0.55, 1.46)
85 to 89	0.96 (0.58, 1.52)
90 to 94	1.00 (0.59, 1.58)
95 plus	1.17 (0.69, 1.86)

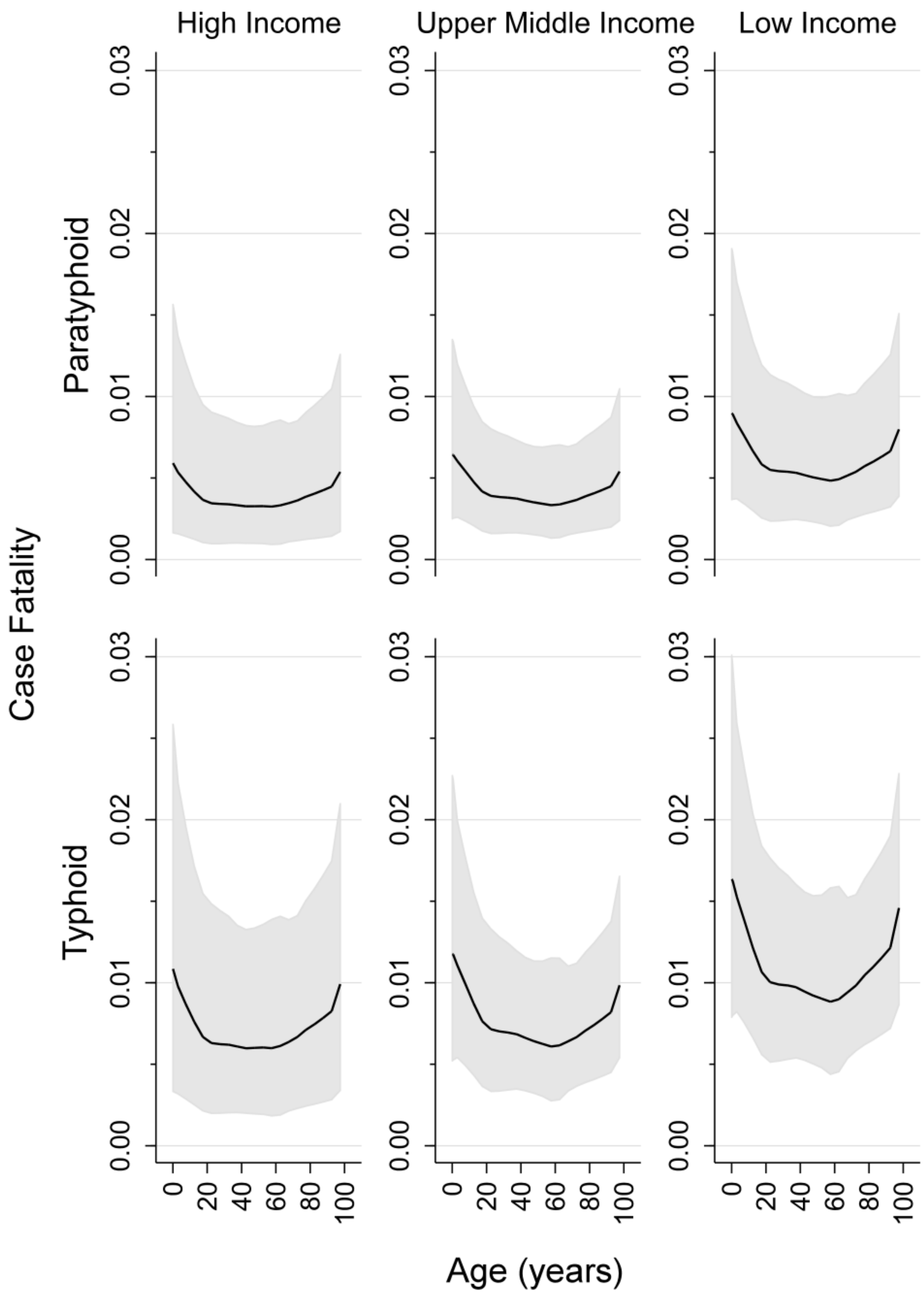

Figure S17: Case fatality estimates for paratyphoid (top row) and typhoid (bottom row) in high income (left column), upper middle income (middle) and low income (right) countries, by age. 95% uncertainty intervals are represented by shaded areas

Table S7: Numbers of typhoid/paratyphoid deaths and mortality rates (MR) by region, for 1990 and 2017 and the percent change in mortality rates between 1990 and 2017, with 95% uncertainty intervals.

Region	1990		2017		Percent change
	Deaths (hundreds)	MR (per million)	Deaths (hundreds)	MR (per million)	
Asia Pacific, High Income	0.21 (0.04, 0.34)	0.11 (0.02, 0.18)	0.01 (0.01, 0.03)	0.00 (0.00, 0.01)	-95.5 (-98.4, -82.0)
Asia, Central	0.14 (0.03, 0.36)	0.26 (0.05, 0.70)	0.02 (0.01, 0.04)	0.03 (0.01, 0.05)	-85.4 (-95.4, -69.8)
Asia, East	57.6 (28.4, 103.1)	4.4 (2.2, 7.9)	37.0 (18.8, 64.5)	3.4 (1.7, 5.9)	-23.9 (-33.4, -15.6)
Asia, South	1,738.2 (999.1, 2,788.1)	129.3 (74.1, 207.7)	946.7 (545.6, 1,530.8)	50.7 (29.2, 82.0)	-60.6 (-65.5, -55.6)
Asia, Southeast	283.9 (164.7, 454.3)	53.1 (30.8, 84.9)	128.0 (72.3, 209.8)	20.0 (11.3, 32.6)	-62.5 (-67.0, -59.1)
Australasia	0.00 (0.00, 0.00)	0.01 (0.00, 0.01)	0.00 (0.00, 0.00)	0.00 (0.00, 0.00)	-79.9 (-86.5, -54.8)
Caribbean	0.34 (0.20, 0.58)	0.94 (0.57, 1.55)	0.20 (0.10, 0.36)	0.46 (0.23, 0.83)	-52.0 (-64.3, -38.5)
Europe, Central	0.31 (0.06, 0.55)	0.23 (0.04, 0.41)	0.03 (0.02, 0.05)	0.02 (0.01, 0.03)	-87.4 (-95.9, -66.2)
Europe, Eastern	0.05 (0.03, 0.06)	0.02 (0.01, 0.02)	0.01 (0.01, 0.02)	0.00 (0.00, 0.01)	-78.1 (-85.0, -67.0)
Europe, Western	0.31 (0.19, 0.37)	0.06 (0.04, 0.07)	0.14 (0.08, 0.19)	0.01 (0.01, 0.02)	-75.7 (-81.5, -68.5)
Latin America, Andean	0.06 (0.03, 0.11)	0.18 (0.09, 0.33)	0.07 (0.03, 0.13)	0.12 (0.06, 0.22)	-35.8 (-44.1, -26.7)
Latin America, Central	9.6 (6.1, 12.6)	7.3 (4.7, 9.9)	0.98 (0.61, 2.50)	0.41 (0.26, 1.07)	-94.3 (-96.1, -86.3)
Latin America, Southern	1.3 (0.8, 1.6)	2.8 (1.8, 3.4)	0.03 (0.02, 0.06)	0.04 (0.03, 0.08)	-98.4 (-99.0, -97.2)
Latin America, Tropical	0.27 (0.13, 0.52)	0.20 (0.09, 0.37)	0.30 (0.14, 0.55)	0.13 (0.06, 0.25)	-32.7 (-37.7, -27.8)
North Africa / Middle East	51.3 (26.8, 88.4)	12.4 (6.5, 21.4)	27.7 (14.9, 48.2)	4.4 (2.4, 7.6)	-64.3 (-68.9, -56.4)
North America, High Income	0.16 (0.07, 0.19)	0.05 (0.02, 0.06)	0.03 (0.02, 0.08)	0.01 (0.00, 0.02)	-85.8 (-93.0, -61.0)
Oceania	3.1 (1.6, 5.2)	39.2 (21.0, 66.0)	2.2 (1.2, 4.0)	15.5 (8.1, 27.4)	-60.5 (-66.1, -54.4)
Sub-Saharan Africa, Central	7.5 (3.4, 13.7)	10.7 (5.0, 19.3)	12.5 (6.4, 22.1)	8.1 (4.2, 14.3)	-23.2 (-36.4, 8.0)
Sub-Saharan Africa, East	61.7 (28.5, 112.3)	24.9 (12.0, 43.9)	99.9 (53.0, 170.7)	19.7 (10.4, 33.5)	-19.2 (-35.8, 6.4)
Sub-Saharan Africa, Southern	0.19 (0.10, 0.34)	0.30 (0.16, 0.54)	0.19 (0.10, 0.34)	0.23 (0.12, 0.41)	-22.4 (-30.7, -12.1)
Sub-Saharan Africa, West	89.2 (45.4, 158.3)	36.3 (19.0, 63.5)	103.2 (53.4, 183.6)	18.4 (9.7, 32.4)	-49.4 (-54.5, -42.9)
Global	2,305.4 (1,312.3, 3,726.3)	39.0 (22.3, 62.9)	1,359.2 (769.0, 2,188.8)	18.9 (10.7, 30.4)	-51.5 (-57.4, -45.8)

Table S8: Number of typhoid/paratyphoid cases and deaths, by country or non-sovereign location, in 1990 and 2017

Location	Cases		Deaths	
	1990	2017	1990	2017
Afghanistan	28,748 (23,818, 34,376)	42,756 (34,976, 51,563)	296 (44, 576)	618 (321, 1,083)
Albania	38 (29, 49)	32 (24, 42)	0 (0, 0)	0 (0, 0)
Algeria	29,350 (24,760, 34,429)	9,473 (8,204, 10,893)	276 (132, 493)	94 (44, 175)
American Samoa	96 (80, 115)	33 (28, 39)	1 (0, 1)	0 (0, 0)
Andorra	0 (0, 0)	0 (0, 0)	0 (0, 0)	0 (0, 0)
Angola	17,550 (14,229, 21,696)	27,140 (22,090, 32,958)	135 (52, 265)	247 (114, 473)
Antigua and Barbuda	1 (1, 2)	1 (1, 2)	0 (0, 0)	0 (0, 0)
Argentina	195 (147, 257)	32 (23, 42)	100 (60, 125)	2 (1, 4)
Armenia	5 (4, 7)	4 (3, 5)	0 (0, 0)	0 (0, 0)
Australia	1 (1, 1)	1 (1, 2)	0 (0, 0)	0 (0, 0)
Austria	19 (14, 25)	11 (8, 15)	0 (0, 0)	0 (0, 0)
Azerbaijan	9 (7, 12)	11 (9, 15)	0 (0, 0)	0 (0, 0)
Bahrain	422 (365, 484)	291 (256, 332)	3 (1, 7)	2 (1, 5)
Bangladesh	2,022,009 (1,598,647, 2,537,711)	1,046,157 (857,671, 1,267,974)	22,425 (11,422, 38,183)	12,811 (7,366, 20,775)
Barbados	10 (8, 13)	9 (7, 12)	1 (0, 1)	0 (0, 0)
Belarus	61 (46, 78)	49 (37, 64)	0 (0, 1)	0 (0, 0)
Belgium	17 (13, 22)	7 (5, 10)	1 (0, 1)	0 (0, 0)
Belize	6 (5, 8)	12 (9, 16)	0 (0, 0)	0 (0, 0)
Benin	18,444 (15,002, 22,571)	19,748 (16,371, 23,474)	239 (123, 425)	237 (116, 426)
Bermuda	2 (2, 3)	2 (2, 3)	0 (0, 0)	0 (0, 0)
Bhutan	4,460 (3,703, 5,255)	1,419 (1,215, 1,639)	48 (26, 84)	17 (9, 30)

Location	Cases		Deaths	
	1990	2017	1990	2017
Bolivia	161<br>(122, 214)	173<br>(135, 219)	2<br>(1, 3)	2<br>(1, 3)
Bosnia and Herzegovina	44<br>(34, 57)	33<br>(24, 45)	0<br>(0, 0)	0<br>(0, 0)
Botswana	71<br>(60, 84)	54<br>(47, 63)	1<br>(0, 1)	0<br>(0, 1)
Brazil	3,643<br>(2,765, 4,878)	4,202<br>(3,248, 5,365)	26<br>(12, 49)	29<br>(14, 53)
Brunei	0<br>(0, 0)	0<br>(0, 0)	0<br>(0, 0)	0<br>(0, 0)
Bulgaria	207<br>(162, 271)	199<br>(149, 267)	0<br>(0, 1)	0<br>(0, 0)
Burkina Faso	129,607<br>(100,360, 168,593)	130,095<br>(103,998, 161,924)	1,387<br>(690, 2,475)	1,426<br>(720, 2,610)
Burundi	18,523<br>(15,355, 22,191)	26,326<br>(21,838, 31,375)	193<br>(96, 353)	321<br>(167, 563)
Cambodia	88,491<br>(73,644, 105,924)	36,172<br>(30,909, 41,750)	746<br>(406, 1,302)	328<br>(172, 551)
Cameroon	38,675<br>(31,737, 46,783)	44,787<br>(37,501, 53,104)	466<br>(238, 807)	579<br>(291, 1,066)
Canada	3<br>(2, 4)	1<br>(1, 2)	1<br>(0, 1)	0<br>(0, 0)
Cape Verde	1,305<br>(1,071, 1,568)	318<br>(272, 368)	15<br>(8, 25)	4<br>(2, 8)
Central African Republic	4,586<br>(3,721, 5,587)	4,914<br>(4,064, 5,940)	43<br>(17, 84)	50<br>(24, 94)
Chad	26,027<br>(21,271, 31,603)	32,836<br>(26,947, 39,317)	273<br>(135, 490)	439<br>(224, 799)
Chile	1,340<br>(1,141, 1,564)	437<br>(330, 570)	30<br>(21, 36)	1<br>(1, 3)
China	699,483<br>(627,107, 787,440)	569,618<br>(537,965, 604,842)	5,546<br>(2,721, 9,952)	3,581<br>(1,813, 6,216)
Colombia	2,816<br>(2,358, 3,345)	1,934<br>(1,567, 2,456)	52<br>(36, 64)	3<br>(2, 7)
Comoros	1,136<br>(945, 1,360)	851<br>(726, 996)	17<br>(9, 29)	14<br>(7, 24)
Congo	3,310<br>(2,704, 4,005)	3,114<br>(2,606, 3,722)	36<br>(19, 66)	39<br>(20, 69)
Costa Rica	11<br>(9, 14)	10<br>(8, 13)	1<br>(0, 1)	0<br>(0, 0)
Cote d'Ivoire	41,872<br>(34,411, 50,329)	29,788<br>(24,995, 35,724)	472<br>(236, 828)	415<br>(213, 733)
Croatia	53<br>(40, 67)	52<br>(38, 69)	0<br>(0, 0)	0<br>(0, 0)

Location	Cases		Deaths	
	1990	2017	1990	2017
Cuba	174 (139, 218)	153 (117, 202)	3 (2, 4)	0 (0, 0)
Cyprus	2 (1, 3)	2 (1, 3)	0 (0, 0)	0 (0, 0)
Czech Republic	128 (97, 164)	187 (138, 248)	0 (0, 0)	1 (0, 3)
Democratic Republic of the	59,019 (47,778, 72,277)	85,891 (70,421, 104,558)	521 (228, 956)	907 (451, 1,584)
Denmark	46 (34, 61)	38 (28, 50)	0 (0, 1)	1 (0, 2)
Djibouti	1,521 (1,272, 1,807)	1,548 (1,330, 1,807)	21 (8, 39)	25 (12, 46)
Dominica	2 (2, 3)	1 (1, 2)	0 (0, 0)	0 (0, 0)
Dominican Republic	215 (162, 285)	335 (256, 440)	1 (1, 3)	2 (1, 3)
Ecuador	210 (159, 280)	247 (194, 317)	2 (1, 3)	2 (1, 3)
Egypt	71,388 (61,612, 82,329)	24,276 (20,803, 28,126)	793 (413, 1,354)	290 (147, 504)
El Salvador	218 (164, 292)	144 (113, 185)	2 (1, 5)	2 (1, 3)
Equatorial Guinea	646 (527, 791)	807 (663, 971)	4 (1, 10)	5 (2, 12)
Eritrea	9,640 (7,959, 11,592)	10,446 (8,797, 12,324)	95 (31, 190)	163 (85, 289)
Estonia	25 (19, 34)	24 (18, 31)	0 (0, 0)	0 (0, 0)
Ethiopia	175,232 (149,694, 203,931)	208,683 (181,513, 237,188)	1,909 (853, 3,496)	2,782 (1,472, 4,826)
Federated States of Micronesia	402 (331, 483)	88 (73, 104)	5 (3, 8)	1 (0, 2)
Fiji	1,700 (1,418, 2,017)	523 (443, 616)	14 (7, 25)	5 (2, 8)
Finland	0 (0, 1)	0 (0, 0)	0 (0, 1)	0 (0, 0)
France	293 (221, 380)	190 (142, 250)	5 (3, 6)	3 (2, 4)
Gabon	1,201 (988, 1,453)	579 (482, 694)	9 (4, 17)	5 (2, 10)
Georgia	10 (7, 13)	6 (5, 8)	0 (0, 0)	0 (0, 0)
Germany	102 (74, 138)	51 (38, 65)	5 (2, 6)	2 (1, 3)

Location	Cases		Deaths	
	1990	2017	1990	2017
Ghana	100,304 (79,644, 126,577)	79,100 (65,646, 94,925)	1,100 (572, 1,934)	1,048 (561, 1,821)
Greece	52 (40, 67)	37 (29, 47)	0 (0, 1)	1 (1, 2)
Greenland	0 (0, 0)	0 (0, 0)	0 (0, 0)	0 (0, 0)
Grenada	2 (2, 3)	2 (2, 3)	0 (0, 0)	0 (0, 0)
Guam	168 (141, 199)	80 (68, 94)	1 (0, 2)	1 (0, 1)
Guatemala	3,481 (2,672, 4,403)	4,045 (3,386, 4,932)	240 (167, 360)	26 (17, 37)
Guinea	22,338 (18,259, 27,064)	19,058 (15,802, 22,709)	291 (147, 524)	244 (123, 436)
Guinea-Bissau	832 (644, 1,071)	677 (528, 865)	10 (5, 18)	9 (4, 17)
Guyana	20 (15, 27)	19 (15, 25)	0 (0, 0)	0 (0, 0)
Haiti	2,220 (1,783, 2,803)	1,720 (1,387, 2,148)	24 (12, 45)	16 (8, 30)
Honduras	176 (133, 238)	227 (176, 290)	2 (1, 4)	2 (1, 5)
Hungary	104 (77, 137)	96 (70, 129)	0 (0, 0)	0 (0, 0)
Iceland	2 (1, 2)	1 (1, 2)	0 (0, 0)	0 (0, 0)
India	16,483,775 (14,109,529,	8,365,375 (7,359,075, 9,432,685)	138,910 (79,885, 220,862)	72,400 (41,825, 117,033)
Indonesia	1,247,882 (1,097,279, 1,406,168)	698,419 (625,608, 774,690)	13,116 (7,227, 21,728)	7,027 (3,884, 11,378)
Iran	62,116 (54,605, 69,622)	16,688 (15,078, 18,305)	554 (273, 984)	137 (67, 242)
Iraq	28,213 (23,815, 33,278)	23,183 (19,686, 27,186)	235 (110, 440)	194 (93, 347)
Ireland	7 (5, 10)	4 (3, 6)	0 (0, 0)	0 (0, 0)
Israel	17 (13, 23)	20 (14, 26)	2 (1, 2)	0 (0, 0)
Italy	183 (137, 240)	82 (61, 108)	2 (1, 3)	1 (0, 1)
Jamaica	53 (41, 70)	52 (39, 67)	0 (0, 1)	0 (0, 1)
Japan	860 (672, 1,073)	716 (550, 918)	3 (1, 3)	1 (0, 2)

Location	Cases		Deaths	
	1990	2017	1990	2017
Jordan	4,862<br>(4,075, 5,698)	2,573<br>(2,200, 2,977)	43<br>(21, 79)	21<br>(10, 39)
Kazakhstan	22<br>(17, 28)	19<br>(15, 26)	13<br>(2, 35)	2<br>(0, 3)
Kenya	54,556<br>(46,432, 63,745)	109,275<br>(95,218, 124,304)	545<br>(287, 935)	1,256<br>(679, 2,099)
Kiribati	354<br>(294, 425)	170<br>(142, 204)	5<br>(2, 8)	2<br>(1, 4)
Kuwait	1,212<br>(1,045, 1,400)	764<br>(668, 869)	0<br>(0, 0)	0<br>(0, 0)
Kyrgyzstan	7<br>(5, 9)	7<br>(5, 9)	0<br>(0, 0)	0<br>(0, 0)
Laos	31,430<br>(26,380, 37,315)	16,767<br>(14,262, 19,461)	351<br>(186, 605)	202<br>(106, 345)
Latvia	39<br>(30, 51)	30<br>(23, 40)	0<br>(0, 0)	0<br>(0, 0)
Lebanon	4,376<br>(3,701, 5,105)	2,731<br>(2,357, 3,155)	41<br>(20, 74)	25<br>(12, 44)
Lesotho	119<br>(101, 139)	79<br>(68, 92)	1<br>(1, 2)	1<br>(0, 2)
Liberia	6,270<br>(5,097, 7,567)	8,658<br>(7,255, 10,288)	73<br>(37, 129)	92<br>(46, 165)
Libya	3,819<br>(3,219, 4,511)	1,442<br>(1,249, 1,655)	34<br>(17, 62)	13<br>(6, 23)
Lithuania	47<br>(36, 60)	36<br>(27, 47)	0<br>(0, 0)	0<br>(0, 0)
Luxembourg	3<br>(2, 4)	2<br>(2, 3)	0<br>(0, 0)	0<br>(0, 0)
Macedonia	14<br>(10, 18)	18<br>(13, 24)	0<br>(0, 0)	0<br>(0, 0)
Madagascar	28,336<br>(20,849, 38,743)	40,056<br>(30,350, 53,508)	337<br>(164, 636)	461<br>(218, 834)
Malawi	31,242<br>(25,897, 37,181)	32,966<br>(27,614, 39,213)	269<br>(66, 562)	436<br>(226, 770)
Malaysia	60,400<br>(51,166, 70,019)	35,144<br>(30,461, 40,166)	423<br>(214, 737)	245<br>(124, 430)
Maldives	651<br>(505, 837)	335<br>(270, 414)	5<br>(2, 9)	2<br>(1, 4)
Mali	35,555<br>(29,159, 42,783)	36,841<br>(30,547, 44,021)	448<br>(222, 798)	530<br>(267, 963)
Malta	0<br>(0, 0)	0<br>(0, 0)	0<br>(0, 0)	0<br>(0, 0)
Marshall Islands	172<br>(140, 209)	53<br>(45, 63)	1<br>(1, 3)	0<br>(0, 1)

Location	Cases		Deaths	
	1990	2017	1990	2017
Mauritania	6,248 (5,150, 7,493)	4,494 (3,762, 5,314)	72 (37, 126)	66 (34, 114)
Mauritius	600 (434, 866)	254 (192, 345)	0 (0, 1)	0 (0, 0)
Mexico	22,772 (19,304, 27,261)	13,367 (10,946, 16,597)	635 (383, 851)	61 (33, 158)
Moldova	5 (4, 7)	4 (3, 6)	0 (0, 1)	0 (0, 0)
Mongolia	2 (2, 3)	5 (4, 7)	0 (0, 0)	0 (0, 0)
Montenegro	5 (4, 7)	6 (4, 8)	0 (0, 0)	0 (0, 0)
Morocco	57,626 (48,894, 67,267)	12,454 (10,760, 14,426)	737 (386, 1,247)	175 (91, 307)
Mozambique	49,091 (40,879, 58,510)	72,343 (60,014, 86,475)	657 (327, 1,175)	1,201 (613, 2,130)
Myanmar	363,062 (306,161, 428,702)	141,588 (122,065, 163,600)	4,207 (2,252, 7,153)	1,742 (945, 2,950)
Namibia	84 (71, 98)	64 (55, 75)	1 (0, 1)	1 (0, 1)
Nepal	343,904 (287,696, 411,990)	145,455 (123,411, 168,236)	2,665 (1,329, 4,434)	1,261 (654, 2,133)
Netherlands	79 (60, 101)	44 (33, 57)	0 (0, 0)	0 (0, 0)
New Zealand	35 (26, 46)	47 (35, 61)	0 (0, 0)	0 (0, 0)
Nicaragua	140 (106, 188)	181 (139, 231)	2 (1, 3)	2 (1, 4)
Niger	35,310 (28,684, 42,952)	37,967 (31,132, 45,888)	434 (219, 801)	433 (215, 791)
Nigeria	322,620 (267,311, 389,023)	376,103 (314,701, 449,606)	2,903 (1,471, 5,173)	4,215 (2,143, 7,694)
North Korea	9,163 (7,937, 10,555)	4,984 (4,373, 5,623)	116 (60, 206)	63 (33, 111)
Northern Mariana Islands	59 (51, 70)	20 (17, 23)	0 (0, 1)	0 (0, 0)
Norway	49 (39, 64)	71 (55, 90)	0 (0, 1)	0 (0, 1)
Oman	6,334 (5,346, 7,512)	1,180 (1,025, 1,338)	43 (15, 90)	9 (3, 19)
Pakistan	1,137,286 (960,636, 1,331,869)	727,834 (612,823, 862,508)	9,773 (5,121, 16,454)	8,175 (4,295, 14,104)
Palestine	3,203 (2,692, 3,784)	3,149 (2,657, 3,698)	43 (22, 74)	37 (20, 65)

Location	Cases		Deaths	
	1990	2017	1990	2017
Panama	58<br>(44, 75)	75<br>(59, 97)	0<br>(0, 1)	1<br>(0, 1)
Papua New Guinea	21,362<br>(17,608, 25,778)	16,850<br>(13,929, 20,328)	227<br>(119, 390)	185<br>(95, 330)
Paraguay	135<br>(102, 181)	114<br>(89, 145)	1<br>(1, 3)	1<br>(1, 2)
Peru	371<br>(284, 475)	482<br>(378, 616)	3<br>(1, 5)	3<br>(2, 6)
Philippines	309,361<br>(263,461, 360,232)	154,353<br>(131,089, 180,986)	1,984<br>(1,228, 4,514)	487<br>(266, 963)
Poland	45<br>(34, 60)	54<br>(39, 73)	5<br>(3, 6)	0<br>(0, 1)
Portugal	197<br>(155, 245)	100<br>(77, 129)	2<br>(2, 3)	0<br>(0, 1)
Puerto Rico	119<br>(99, 145)	86<br>(70, 107)	3<br>(2, 3)	0<br>(0, 0)
Qatar	307<br>(266, 349)	514<br>(449, 588)	3<br>(1, 6)	3<br>(1, 8)
Romania	879<br>(661, 1,140)	318<br>(237, 421)	24<br>(1, 48)	1<br>(0, 1)
Russia	692<br>(531, 911)	667<br>(506, 887)	4<br>(2, 5)	1<br>(0, 2)
Rwanda	19,621<br>(16,172, 23,474)	16,659<br>(14,060, 19,721)	228<br>(118, 409)	240<br>(125, 429)
Saint Lucia	8<br>(7, 10)	6<br>(4, 7)	0<br>(0, 0)	0<br>(0, 0)
Saint Vincent and the Grenadines	5<br>(4, 6)	3<br>(3, 4)	0<br>(0, 0)	0<br>(0, 0)
Samoa	498<br>(412, 593)	168<br>(139, 202)	6<br>(3, 10)	2<br>(1, 3)
Sao Tome and Principe	374<br>(308, 450)	220<br>(186, 258)	6<br>(3, 10)	3<br>(2, 6)
Saudi Arabia	30,394<br>(25,753, 35,591)	8,854<br>(7,739, 10,120)	204<br>(71, 458)	61<br>(20, 133)
Senegal	23,996<br>(19,574, 28,830)	13,824<br>(11,564, 16,323)	319<br>(166, 576)	218<br>(112, 379)
Serbia	110<br>(83, 142)	93<br>(69, 123)	0<br>(0, 1)	0<br>(0, 1)
Seychelles	44<br>(31, 63)	22<br>(17, 31)	0<br>(0, 1)	0<br>(0, 0)
Sierra Leone	14,982<br>(12,346, 18,025)	15,030<br>(12,533, 17,876)	190<br>(97, 333)	177<br>(88, 318)
Singapore	2<br>(1, 2)	1<br>(1, 2)	0<br>(0, 0)	0<br>(0, 0)

Location	Cases		Deaths	
	1990	2017	1990	2017
Slovakia	40<br>(30, 52)	49<br>(36, 64)	0<br>(0, 0)	0<br>(0, 0)
Slovenia	30<br>(23, 39)	37<br>(28, 49)	0<br>(0, 0)	0<br>(0, 0)
Solomon Islands	1,499<br>(1,238, 1,814)	848<br>(702, 1,022)	16<br>(8, 28)	10<br>(5, 17)
Somalia	22,637<br>(18,650, 27,272)	30,464<br>(25,258, 36,184)	197<br>(36, 428)	355<br>(173, 657)
South Africa	1,272<br>(1,131, 1,428)	998<br>(900, 1,101)	10<br>(5, 18)	8<br>(4, 14)
South Korea	10<br>(6, 16)	1<br>(1, 2)	18<br>(2, 31)	1<br>(0, 1)
South Sudan	20,030<br>(16,665, 24,012)	25,030<br>(20,752, 29,991)	171<br>(40, 378)	314<br>(153, 564)
Spain	290<br>(227, 366)	188<br>(141, 248)	4<br>(2, 6)	1<br>(1, 2)
Sri Lanka	115,655<br>(99,052, 133,676)	33,298<br>(28,965, 38,118)	1,092<br>(611, 1,780)	317<br>(174, 522)
Sudan	43,447<br>(36,521, 51,416)	32,526<br>(27,337, 38,422)	413<br>(180, 751)	415<br>(212, 742)
Suriname	10<br>(8, 13)	12<br>(9, 16)	0<br>(0, 0)	0<br>(0, 0)
Swaziland	54<br>(46, 65)	39<br>(33, 46)	1<br>(0, 1)	1<br>(0, 1)
Sweden	77<br>(63, 94)	73<br>(60, 90)	0<br>(0, 0)	0<br>(0, 0)
Switzerland	9<br>(6, 12)	2<br>(2, 3)	7<br>(3, 10)	4<br>(2, 7)
Syria	19,614<br>(16,512, 23,192)	5,487<br>(4,652, 6,424)	237<br>(125, 419)	71<br>(37, 126)
Taiwan	7,621<br>(6,622, 8,760)	2,581<br>(2,278, 2,882)	2<br>(1, 3)	0<br>(0, 0)
Tajikistan	7<br>(5, 9)	9<br>(7, 12)	0<br>(0, 0)	0<br>(0, 0)
Tanzania	89,725<br>(66,989, 119,777)	80,205<br>(62,102, 101,860)	940<br>(281, 1,906)	1,330<br>(635, 2,388)
Thailand	460,058<br>(395,497, 535,556)	156,196<br>(137,111, 175,818)	3,337<br>(1,698, 5,675)	1,093<br>(554, 1,898)
The Bahamas	4<br>(3, 5)	5<br>(4, 7)	0<br>(0, 0)	0<br>(0, 0)
The Gambia	3,520<br>(2,900, 4,262)	2,598<br>(2,168, 3,093)	42<br>(21, 76)	37<br>(18, 69)
Timor-Leste	4,043<br>(3,418, 4,736)	2,435<br>(2,069, 2,827)	44<br>(23, 73)	31<br>(15, 55)

Location	Cases		Deaths	
	1990	2017	1990	2017
Togo	15,063 (12,340, 18,201)	13,950 (11,742, 16,541)	185 (93, 332)	147 (74, 259)
Tonga	347 (290, 412)	95 (79, 113)	2 (1, 4)	1 (0, 1)
Trinidad and Tobago	1 (1, 1)	1 (1, 2)	1 (0, 1)	0 (0, 0)
Tunisia	11,301 (9,571, 13,178)	3,267 (2,850, 3,737)	84 (41, 150)	23 (11, 42)
Turkey	82,321 (70,375, 96,188)	23,025 (20,046, 26,520)	684 (340, 1,238)	183 (88, 328)
Turkmenistan	6 (5, 8)	6 (4, 7)	0 (0, 0)	0 (0, 0)
Uganda	44,733 (36,746, 54,049)	56,512 (46,605, 67,603)	353 (166, 665)	660 (333, 1,158)
Ukraine	321 (246, 417)	307 (230, 401)	0 (0, 1)	0 (0, 0)
United Arab Emirates	1,950 (1,688, 2,243)	1,702 (1,489, 1,937)	18 (6, 38)	15 (5, 33)
United Kingdom	333 (270, 416)	359 (280, 456)	1 (0, 1)	0 (0, 0)
United States	4,758 (4,062, 5,596)	4,137 (3,246, 5,301)	16 (6, 19)	3 (2, 8)
Uruguay	42 (32, 54)	3 (2, 3)	3 (2, 3)	0 (0, 0)
Uzbekistan	18 (14, 23)	25 (19, 33)	0 (0, 0)	0 (0, 0)
Vanuatu	735 (607, 886)	441 (364, 534)	7 (4, 13)	5 (2, 9)
Venezuela	26 (20, 34)	25 (19, 34)	25 (14, 35)	1 (1, 2)
Vietnam	343,120 (280,465, 417,400)	137,599 (115,894, 163,521)	3,048 (1,663, 5,123)	1,304 (701, 2,210)
Virgin Islands, U.S.	1 (1, 2)	2 (1, 2)	0 (0, 0)	0 (0, 0)
Yemen	39,631 (32,581, 47,553)	30,051 (25,006, 35,852)	382 (84, 721)	384 (195, 672)
Zambia	18,273 (15,195, 21,991)	27,670 (23,047, 32,631)	238 (92, 442)	422 (215, 754)
Zimbabwe	551 (463, 659)	656 (552, 781)	6 (3, 10)	9 (5, 16)

Table S9: Typhoid/paratyphoid incidence and mortality rates, by country or non-sovereign location, in 1990 and 2017

Location	Incidence (per 100,000)		Mortality (per million)	
	1990	2017	1990	2017
Afghanistan	244.3 (209.4, 284.9)	104.4 (88.8, 121.9)	25.5 (4.5, 48.6)	14.9 (7.6, 26.0)
Albania	1.4 (1.1, 1.8)	0.90 (0.69, 1.17)	0.06 (0.03, 0.12)	0.04 (0.02, 0.08)
Algeria	95.5 (82.4, 109.9)	24.1 (20.9, 27.8)	8.7 (4.2, 15.6)	2.4 (1.1, 4.5)
American Samoa	172.4 (146.2, 202.8)	55.7 (47.3, 65.0)	12.7 (6.5, 22.7)	4.0 (2.0, 7.4)
Andorra	0.26 (0.19, 0.35)	0.15 (0.11, 0.20)	0.01 (<0.01, 0.02)	0.01 (<0.01, 0.01)
Angola	140.1 (118.4, 166.7)	75.5 (63.9, 88.9)	10.4 (4.3, 20.2)	6.6 (3.1, 12.3)
Antigua and Barbuda	2.3 (1.8, 2.9)	1.5 (1.2, 2.0)	0.75 (0.48, 0.91)	0.03 (0.01, 0.06)
Argentina	0.60 (0.45, 0.79)	0.07 (0.05, 0.09)	3.2 (1.9, 3.9)	0.04 (0.02, 0.07)
Armenia	0.18 (0.14, 0.23)	0.11 (0.08, 0.14)	0.02 (0.01, 0.03)	0.01 (0.01, 0.02)
Australia	0.01 (<0.01, 0.01)	0.01 (<0.01, 0.01)	0.01 (<0.01, 0.01)	<0.01 (<0.01, <0.01)
Austria	0.23 (0.17, 0.31)	0.12 (0.09, 0.16)	<0.01 (<0.01, 0.01)	<0.01 (<0.01, 0.01)
Azerbaijan	0.16 (0.12, 0.20)	0.11 (0.09, 0.15)	0.01 (0.01, 0.02)	0.01 (<0.01, 0.01)
Bahrain	77.8 (67.2, 89.1)	23.2 (20.1, 26.7)	5.7 (1.9, 12.6)	1.8 (0.6, 4.0)
Bangladesh	1,459.2 (1,206.8, 1,772.1)	641.2 (527.2, 779.8)	157.7 (81.8, 265.2)	78.7 (45.2, 126.8)
Barbados	3.7 (2.9, 4.7)	2.4 (1.9, 3.0)	2.0 (1.4, 2.4)	0.07 (0.05, 0.15)
Belarus	0.51 (0.40, 0.66)	0.40 (0.30, 0.50)	0.02 (0.01, 0.05)	0.02 (0.01, 0.03)
Belgium	0.16 (0.12, 0.21)	0.06 (0.05, 0.08)	0.05 (0.03, 0.07)	<0.01 (<0.01, 0.01)
Belize	4.2 (3.2, 5.6)	3.4 (2.6, 4.5)	0.24 (0.11, 0.44)	0.18 (0.08, 0.32)
Benin	292.5 (249.8, 341.5)	136.2 (116.5, 157.7)	35.7 (18.5, 61.8)	16.0 (8.0, 28.1)
Bermuda	3.6 (2.8, 4.7)	2.8 (2.2, 3.6)	0.46 (0.24, 0.62)	0.02 (0.01, 0.03)
Bhutan	646.4 (551.1, 748.5)	144.9 (124.2, 168.0)	67.4 (36.7, 117.5)	17.4 (8.8, 31.2)
Bolivia	3.2 (2.4, 4.2)	1.7 (1.3, 2.1)	0.33 (0.17, 0.59)	0.18 (0.09, 0.31)
Bosnia and Herzegovina	0.98 (0.76, 1.25)	0.69 (0.53, 0.90)	0.04 (0.02, 0.08)	0.03 (0.01, 0.06)

Location	Incidence (per 100,000)		Mortality (per million)	
	1990	2017	1990	2017
Botswana	4.4 (3.8, 5.1)	2.2 (2.0, 2.6)	0.34 (0.16, 0.64)	0.21 (0.10, 0.37)
Brazil	2.8 (2.1, 3.6)	1.9 (1.5, 2.4)	0.19 (0.09, 0.36)	0.13 (0.06, 0.24)
Brunei	0.01 (0.01, 0.02)	0.01 (0.01, 0.02)	<0.01 (<0.01, <0.01)	<0.01 (<0.01, <0.01)
Bulgaria	1.9 (1.5, 2.3)	1.8 (1.4, 2.3)	0.05 (0.03, 0.07)	<0.01 (<0.01, 0.01)
Burkina Faso	1,050.5 (863.1, 1,287.0)	491.8 (408.9, 591.0)	110.4 (57.5, 195.4)	52.2 (26.5, 93.3)
Burundi	268.5 (231.1, 311.0)	193.5 (166.1, 225.0)	27.1 (13.9, 49.3)	22.6 (11.8, 39.3)
Cambodia	684.7 (586.8, 794.1)	211.5 (181.1, 242.8)	58.7 (31.7, 101.9)	19.2 (10.1, 32.4)
Cameroon	297.5 (253.4, 348.8)	131.1 (112.4, 151.9)	35.1 (18.5, 60.0)	16.6 (8.5, 29.8)
Canada	0.01 (0.01, 0.02)	<0.01 (<0.01, 0.01)	0.02 (0.01, 0.02)	<0.01 (<0.01, 0.01)
Cape Verde	296.2 (253.6, 346.7)	56.7 (48.4, 65.9)	31.8 (16.8, 53.9)	8.1 (4.2, 14.2)
Central African Republic	137.4 (116.3, 161.8)	89.0 (75.3, 106.1)	12.5 (5.0, 23.3)	9.0 (4.4, 16.7)
Chad	339.8 (291.0, 396.0)	164.2 (141.1, 190.1)	34.7 (17.7, 59.9)	20.5 (10.7, 36.7)
Chile	10.6 (9.0, 12.4)	2.2 (1.7, 2.8)	2.5 (1.8, 3.0)	0.05 (0.03, 0.11)
China	56.2 (50.4, 63.5)	52.1 (48.4, 56.3)	4.5 (2.2, 8.0)	3.4 (1.8, 6.0)
Colombia	10.3 (8.5, 12.6)	3.7 (3.0, 4.6)	1.9 (1.3, 2.3)	0.06 (0.04, 0.13)
Comoros	197.6 (169.9, 229.4)	106.2 (91.6, 123.0)	28.8 (15.2, 49.4)	16.9 (8.8, 29.5)
Congo	110.6 (93.6, 130.1)	55.5 (47.2, 65.2)	11.6 (6.0, 20.8)	6.9 (3.5, 12.1)
Costa Rica	0.44 (0.34, 0.55)	0.21 (0.16, 0.28)	0.26 (0.16, 0.33)	0.03 (0.02, 0.04)
Cote d'Ivoire	269.3 (230.6, 312.9)	99.3 (85.1, 116.2)	29.8 (15.3, 52.3)	13.3 (6.9, 23.6)
Croatia	0.91 (0.71, 1.13)	0.81 (0.62, 1.04)	0.02 (<0.01, 0.02)	<0.01 (<0.01, <0.01)
Cuba	1.6 (1.3, 2.0)	1.1 (0.8, 1.4)	0.28 (0.16, 0.37)	<0.01 (<0.01, 0.01)
Cyprus	0.26 (0.19, 0.35)	0.15 (0.11, 0.19)	0.01 (<0.01, 0.03)	0.01 (<0.01, 0.01)
Czech Republic	1.1 (0.8, 1.4)	1.2 (0.9, 1.6)	0.01 (<0.01, 0.01)	0.06 (0.01, 0.14)
Democratic Republic of the Congo	124.7 (105.8, 147.8)	86.2 (72.8, 101.6)	10.8 (4.9, 19.5)	8.9 (4.5, 15.4)

Location	Incidence (per 100,000)		Mortality (per million)	
	1990	2017	1990	2017
Denmark	0.78 (0.58, 1.04)	0.57 (0.44, 0.76)	0.06 (0.03, 0.08)	0.09 (0.02, 0.14)
Djibouti	248.0 (213.9, 286.5)	127.7 (110.5, 147.6)	32.9 (13.9, 60.7)	19.9 (9.9, 36.9)
Dominica	2.8 (2.2, 3.7)	1.9 (1.4, 2.4)	0.17 (0.08, 0.32)	0.10 (0.05, 0.19)
Dominican Republic	3.8 (2.9, 5.0)	3.3 (2.5, 4.4)	0.22 (0.10, 0.41)	0.18 (0.08, 0.33)
Ecuador	2.6 (2.0, 3.3)	1.5 (1.2, 2.0)	0.18 (0.09, 0.34)	0.11 (0.05, 0.20)
Egypt	112.7 (98.7, 128.0)	23.1 (20.0, 26.7)	12.2 (6.3, 20.7)	2.7 (1.4, 4.7)
El Salvador	5.2 (4.0, 6.9)	2.4 (1.9, 3.1)	0.56 (0.28, 0.99)	0.25 (0.13, 0.44)
Equatorial Guinea	124.4 (105.5, 146.4)	47.8 (40.4, 56.3)	8.0 (2.6, 18.7)	3.0 (0.9, 7.3)
Eritrea	261.7 (225.0, 303.1)	147.7 (127.0, 171.3)	25.6 (9.2, 49.3)	22.4 (11.6, 39.2)
Estonia	1.4 (1.1, 1.8)	1.2 (0.9, 1.6)	0.01 (0.01, 0.02)	0.01 (<0.01, 0.02)
Ethiopia	271.4 (240.3, 304.9)	163.0 (144.4, 182.2)	28.2 (13.3, 50.1)	20.9 (11.1, 36.0)
Federated States of Micronesia	310.4 (264.0, 362.9)	76.8 (65.2, 90.0)	36.4 (19.5, 61.3)	9.1 (4.2, 16.0)
Fiji	191.9 (162.6, 223.8)	56.8 (48.2, 66.8)	15.7 (7.9, 27.8)	4.9 (2.4, 8.9)
Finland	0.01 (0.01, 0.01)	0.01 (<0.01, 0.01)	0.09 (0.05, 0.13)	0.01 (<0.01, 0.01)
France	0.46 (0.35, 0.59)	0.27 (0.20, 0.36)	0.06 (0.04, 0.07)	0.02 (0.01, 0.02)
Gabon	103.3 (88.0, 122.1)	30.6 (25.8, 36.5)	7.4 (3.5, 13.6)	2.7 (1.3, 5.0)
Georgia	0.16 (0.12, 0.21)	0.13 (0.10, 0.17)	0.02 (0.01, 0.03)	0.01 (0.01, 0.02)
Germany	0.12 (0.08, 0.15)	0.06 (0.04, 0.08)	0.05 (0.03, 0.06)	0.01 (<0.01, 0.01)
Ghana	544.3 (449.7, 658.3)	228.3 (193.5, 270.7)	59.5 (31.4, 103.5)	29.8 (16.0, 51.5)
Greece	0.47 (0.36, 0.60)	0.33 (0.26, 0.43)	0.04 (0.03, 0.08)	0.06 (0.04, 0.07)
Greenland	0.03 (0.02, 0.04)	0.03 (0.02, 0.04)	<0.01 (<0.01, <0.01)	<0.01 (<0.01, <0.01)
Grenada	3.1 (2.4, 4.0)	1.7 (1.3, 2.2)	0.19 (0.09, 0.35)	0.09 (0.04, 0.18)
Guam	116.1 (97.7, 137.5)	49.5 (41.8, 58.4)	7.5 (2.6, 16.2)	3.3 (1.1, 7.5)
Guatemala	59.1 (46.4, 75.8)	28.6 (23.5, 35.5)	33.5 (24.1, 58.9)	1.9 (1.2, 2.7)

Location	Incidence (per 100,000)		Mortality (per million)	
	1990	2017	1990	2017
Guinea	296.1 (253.7, 345.8)	129.1 (110.8, 149.9)	36.2 (18.8, 62.8)	16.0 (8.2, 28.2)
Guinea-Bissau	64.8 (52.8, 79.4)	29.5 (23.8, 36.7)	7.3 (3.7, 13.2)	3.8 (1.9, 6.9)
Guyana	3.3 (2.5, 4.3)	2.7 (2.1, 3.5)	0.28 (0.14, 0.50)	0.21 (0.10, 0.39)
Haiti	29.4 (24.3, 36.0)	13.1 (10.7, 16.1)	3.0 (1.5, 5.6)	1.2 (0.6, 2.2)
Honduras	5.2 (4.0, 6.9)	2.9 (2.2, 3.7)	0.62 (0.31, 1.11)	0.31 (0.16, 0.56)
Hungary	0.77 (0.58, 0.99)	0.62 (0.47, 0.81)	0.01 (<0.01, 0.01)	<0.01 (<0.01, <0.01)
Iceland	0.68 (0.54, 0.86)	0.42 (0.32, 0.56)	0.09 (0.05, 0.12)	0.02 (0.01, 0.03)
India	1,604.4 (1,401.0, 1,833.0)	586.3 (515.7, 661.8)	133.8 (76.9, 213.7)	51.0 (29.6, 82.4)
Indonesia	579.4 (515.2, 645.3)	273.1 (244.1, 303.6)	59.9 (33.1, 98.9)	27.6 (15.4, 44.8)
Iran	87.8 (78.8, 97.1)	21.6 (19.3, 23.9)	7.5 (3.7, 13.1)	1.8 (0.9, 3.2)
Iraq	134.1 (116.3, 154.6)	47.1 (40.5, 54.7)	10.9 (5.2, 19.9)	3.9 (1.9, 7.0)
Ireland	0.20 (0.15, 0.26)	0.08 (0.06, 0.11)	<0.01 (<0.01, 0.01)	<0.01 (<0.01, <0.01)
Israel	0.36 (0.27, 0.48)	0.22 (0.16, 0.29)	0.32 (0.20, 0.40)	0.01 (<0.01, 0.03)
Italy	0.29 (0.21, 0.38)	0.13 (0.09, 0.17)	0.03 (0.02, 0.04)	0.01 (<0.01, 0.01)
Jamaica	2.6 (2.0, 3.3)	1.8 (1.4, 2.3)	0.14 (0.07, 0.25)	0.10 (0.04, 0.18)
Japan	0.62 (0.50, 0.75)	0.37 (0.30, 0.46)	0.03 (0.01, 0.03)	<0.01 (<0.01, <0.01)
Jordan	104.9 (90.4, 121.1)	21.7 (18.8, 25.0)	9.0 (4.4, 16.5)	1.8 (0.9, 3.2)
Kazakhstan	0.14 (0.11, 0.19)	0.11 (0.08, 0.14)	0.95 (0.16, 2.58)	0.09 (0.02, 0.18)
Kenya	183.3 (161.2, 207.3)	188.0 (166.6, 211.5)	18.2 (9.8, 30.9)	21.4 (11.5, 35.7)
Kiribati	411.3 (347.7, 482.6)	128.6 (108.8, 152.1)	52.7 (28.9, 88.5)	17.7 (9.3, 30.5)
Kuwait	63.2 (54.6, 72.7)	20.1 (17.3, 23.1)	0.04 (0.03, 0.06)	<0.01 (<0.01, 0.01)
Kyrgyzstan	0.20 (0.15, 0.26)	0.13 (0.10, 0.17)	0.02 (0.01, 0.03)	0.01 (0.01, 0.02)
Laos	622.9 (536.6, 721.3)	220.6 (188.8, 255.3)	67.0 (35.6, 114.7)	26.3 (13.8, 45.1)
Latvia	1.2 (1.0, 1.6)	1.1 (0.9, 1.5)	0.01 (<0.01, 0.03)	<0.01 (<0.01, 0.01)

Location	Incidence (per 100,000)		Mortality (per million)	
	1990	2017	1990	2017
Lebanon	90.4 (78.3, 103.9)	30.3 (26.2, 35.0)	8.3 (4.0, 14.8)	2.7 (1.3, 4.9)
Lesotho	5.6 (4.8, 6.4)	3.7 (3.2, 4.2)	0.57 (0.30, 0.98)	0.44 (0.23, 0.79)
Liberia	253.8 (216.4, 296.6)	152.1 (130.4, 177.2)	28.1 (14.3, 48.7)	16.0 (8.2, 28.2)
Libya	74.7 (64.7, 86.5)	20.5 (17.8, 23.5)	6.6 (3.3, 11.9)	1.9 (0.9, 3.3)
Lithuania	1.1 (0.9, 1.4)	0.91 (0.71, 1.15)	0.02 (0.01, 0.04)	0.01 (<0.01, 0.01)
Luxembourg	0.65 (0.49, 0.84)	0.36 (0.27, 0.49)	0.09 (0.05, 0.16)	0.01 (<0.01, 0.02)
Macedonia	0.68 (0.51, 0.88)	0.64 (0.48, 0.84)	0.03 (0.01, 0.06)	0.03 (0.01, 0.05)
Madagascar	192.9 (149.6, 249.5)	125.8 (98.7, 162.2)	22.3 (11.1, 40.4)	14.2 (7.1, 25.2)
Malawi	261.6 (224.7, 301.7)	155.5 (133.8, 180.3)	22.0 (5.9, 44.6)	19.9 (10.4, 34.9)
Malaysia	297.8 (256.0, 341.7)	116.4 (100.1, 134.4)	20.6 (10.5, 35.6)	8.2 (4.1, 14.2)
Maldives	235.4 (189.9, 293.0)	77.4 (61.1, 97.4)	16.9 (8.2, 30.8)	5.1 (2.4, 9.2)
Mali	327.8 (280.7, 379.9)	142.5 (122.2, 166.0)	38.7 (19.8, 67.5)	18.9 (9.7, 33.6)
Malta	0.08 (0.06, 0.11)	0.03 (0.02, 0.04)	0.02 (0.01, 0.03)	<0.01 (<0.01, 0.01)
Marshall Islands	289.4 (245.8, 340.1)	85.4 (72.5, 100.2)	24.4 (12.2, 43.1)	7.5 (3.7, 13.6)
Mauritania	243.6 (208.4, 283.7)	94.7 (81.3, 109.1)	27.5 (14.6, 47.0)	13.3 (6.8, 22.6)
Mauritius	51.0 (37.2, 72.9)	23.6 (17.4, 33.6)	0.40 (0.17, 0.53)	0.01 (0.01, 0.05)
Mexico	34.6 (28.7, 43.4)	11.3 (9.2, 14.2)	9.9 (6.1, 13.6)	0.54 (0.28, 1.39)
Moldova	0.12 (0.09, 0.16)	0.10 (0.07, 0.13)	0.08 (0.03, 0.13)	0.01 (0.01, 0.03)
Mongolia	0.16 (0.13, 0.21)	0.18 (0.14, 0.24)	0.02 (0.01, 0.03)	0.02 (0.01, 0.03)
Montenegro	0.83 (0.64, 1.07)	0.70 (0.54, 0.90)	0.04 (0.02, 0.07)	0.03 (0.01, 0.06)
Morocco	197.7 (170.6, 228.3)	35.0 (30.2, 40.6)	24.4 (12.7, 41.1)	5.0 (2.6, 8.7)
Mozambique	276.6 (239.2, 318.9)	189.4 (162.9, 220.9)	35.8 (18.3, 63.5)	30.2 (15.7, 52.4)
Myanmar	776.1 (665.7, 907.5)	267.2 (229.2, 309.9)	89.5 (48.1, 152.6)	33.1 (18.0, 55.6)
Namibia	4.9 (4.3, 5.6)	2.4 (2.1, 2.8)	0.38 (0.18, 0.71)	0.21 (0.10, 0.40)

Location	Incidence (per 100,000)		Mortality (per million)	
	1990	2017	1990	2017
Nepal	1,442.2 (1,239.7, 1,685.9)	448.6 (383.4, 521.4)	111.9 (56.9, 184.4)	39.0 (20.2, 66.1)
Netherlands	0.48 (0.38, 0.62)	0.24 (0.18, 0.32)	0.01 (<0.01, 0.02)	<0.01 (<0.01, <0.01)
New Zealand	1.0 (0.8, 1.3)	1.1 (0.8, 1.4)	0.01 (<0.01, 0.01)	<0.01 (<0.01, <0.01)
Nicaragua	5.0 (3.9, 6.7)	3.2 (2.5, 4.2)	0.54 (0.28, 0.97)	0.33 (0.17, 0.59)
Niger	338.9 (290.7, 396.7)	133.9 (115.0, 155.2)	39.6 (20.7, 71.8)	14.7 (7.4, 26.6)
Nigeria	298.7 (255.5, 349.5)	146.6 (126.2, 170.3)	26.1 (13.2, 45.3)	15.4 (7.9, 27.8)
North Korea	44.8 (38.8, 51.7)	22.4 (19.3, 25.8)	5.7 (3.0, 10.1)	2.9 (1.5, 5.2)
Northern Mariana Islands	128.8 (108.6, 151.7)	51.2 (43.1, 60.4)	7.4 (2.5, 16.2)	2.9 (1.0, 6.7)
Norway	1.0 (0.8, 1.3)	1.2 (0.9, 1.6)	0.06 (0.03, 0.21)	0.04 (0.01, 0.06)
Oman	283.8 (243.9, 329.9)	27.6 (23.9, 31.7)	18.9 (6.4, 40.2)	2.1 (0.7, 4.7)
Pakistan	847.7 (732.7, 972.0)	294.7 (251.2, 345.1)	70.0 (37.2, 118.1)	32.0 (16.8, 55.5)
Palestine	130.1 (112.3, 149.9)	55.9 (48.2, 64.7)	16.9 (9.0, 29.0)	6.5 (3.5, 11.3)
Panama	2.9 (2.2, 3.8)	1.9 (1.5, 2.5)	0.21 (0.10, 0.38)	0.14 (0.07, 0.26)
Papua New Guinea	442.3 (373.6, 519.9)	161.1 (135.7, 190.2)	46.1 (24.5, 78.7)	17.3 (9.0, 30.8)
Paraguay	4.1 (3.1, 5.5)	1.8 (1.4, 2.3)	0.42 (0.21, 0.75)	0.18 (0.09, 0.32)
Peru	2.1 (1.6, 2.6)	1.5 (1.2, 1.9)	0.14 (0.07, 0.26)	0.10 (0.05, 0.19)
Philippines	411.4 (356.6, 470.3)	139.5 (119.3, 162.8)	35.6 (21.9, 84.7)	5.3 (2.9, 10.4)
Poland	0.10 (0.08, 0.14)	0.09 (0.07, 0.12)	0.12 (0.08, 0.14)	0.01 (<0.01, 0.01)
Portugal	1.8 (1.4, 2.2)	0.87 (0.66, 1.12)	0.20 (0.17, 0.26)	0.02 (0.01, 0.03)
Puerto Rico	3.3 (2.7, 4.0)	1.8 (1.5, 2.1)	0.81 (0.52, 0.99)	0.02 (0.01, 0.04)
Qatar	69.1 (59.7, 79.5)	21.6 (18.8, 24.8)	6.0 (2.0, 13.4)	1.6 (0.5, 3.4)
Romania	3.4 (2.6, 4.4)	1.2 (0.9, 1.5)	1.0 (0.0, 2.1)	0.02 (0.01, 0.03)
Russian Federation	0.42 (0.32, 0.55)	0.38 (0.29, 0.49)	0.02 (0.02, 0.03)	<0.01 (<0.01, 0.01)
Rwanda	214.7 (184.3, 248.7)	110.8 (95.6, 128.5)	23.8 (12.5, 42.6)	15.3 (7.9, 27.0)

Location	Incidence (per 100,000)		Mortality (per million)	
	1990	2017	1990	2017
Saint Lucia	7.3 (5.8, 9.2)	2.9 (2.3, 3.6)	1.5 (0.9, 1.9)	0.03 (0.02, 0.06)
Saint Vincent and the Grenadines	5.4 (4.3, 6.9)	2.6 (2.1, 3.2)	1.2 (0.8, 1.5)	0.05 (0.03, 0.08)
Samoa	244.8 (208.0, 285.7)	74.9 (63.5, 88.1)	28.8 (15.7, 49.2)	7.9 (4.0, 13.7)
Sao Tome and Principe	242.4 (207.4, 282.6)	94.2 (81.1, 109.3)	34.3 (17.8, 60.0)	14.4 (7.4, 25.5)
Saudi Arabia	160.2 (138.7, 185.3)	26.6 (23.0, 30.9)	10.5 (3.6, 23.2)	1.9 (0.6, 4.1)
Senegal	247.0 (211.7, 286.8)	77.8 (66.7, 89.8)	31.1 (16.4, 54.6)	11.8 (6.1, 20.6)
Serbia	1.0 (0.8, 1.3)	0.75 (0.57, 0.97)	0.04 (0.02, 0.09)	0.03 (0.01, 0.06)
Seychelles	54.5 (39.5, 77.4)	24.7 (18.1, 35.0)	3.9 (1.8, 7.5)	1.8 (0.8, 3.4)
Sierra Leone	325.3 (279.4, 380.3)	158.4 (135.5, 184.3)	38.5 (19.8, 66.6)	18.1 (9.1, 31.9)
Singapore	0.06 (0.05, 0.08)	0.02 (0.02, 0.03)	0.04 (0.01, 0.06)	<0.01 (<0.01, 0.01)
Slovakia	0.69 (0.52, 0.90)	0.65 (0.50, 0.85)	0.03 (0.01, 0.07)	0.03 (0.01, 0.07)
Slovenia	1.3 (1.0, 1.7)	1.2 (0.9, 1.5)	0.01 (<0.01, 0.01)	0.01 (<0.01, 0.01)
Solomon Islands	356.2 (303.7, 417.1)	114.4 (96.7, 134.3)	39.4 (20.5, 67.4)	13.0 (6.8, 22.4)
Somalia	251.7 (217.2, 291.8)	143.8 (123.8, 166.1)	21.5 (4.8, 45.3)	16.3 (8.1, 30.0)
South Africa	3.0 (2.7, 3.4)	1.8 (1.6, 2.0)	0.23 (0.11, 0.42)	0.14 (0.07, 0.26)
South Korea	0.02 (0.01, 0.03)	<0.01 (<0.01, <0.01)	0.66 (0.07, 1.22)	0.01 (<0.01, 0.02)
South Sudan	277.6 (238.1, 323.8)	199.9 (171.7, 232.8)	23.6 (6.1, 51.1)	24.0 (12.1, 42.3)
Spain	0.68 (0.54, 0.87)	0.37 (0.28, 0.50)	0.09 (0.05, 0.12)	0.01 (0.01, 0.02)
Sri Lanka	615.1 (530.8, 707.5)	166.4 (143.5, 192.4)	58.1 (32.6, 94.8)	16.0 (8.7, 26.4)
Sudan	182.7 (157.6, 211.5)	67.8 (58.3, 78.8)	17.2 (7.7, 31.1)	8.4 (4.3, 14.9)
Suriname	3.1 (2.4, 4.0)	2.1 (1.6, 2.7)	0.18 (0.08, 0.33)	0.11 (0.05, 0.19)
Swaziland	5.3 (4.6, 6.1)	3.0 (2.6, 3.4)	0.58 (0.30, 1.00)	0.40 (0.20, 0.73)
Sweden	0.85 (0.69, 1.02)	0.69 (0.56, 0.83)	0.02 (0.01, 0.04)	<0.01 (<0.01, 0.01)
Switzerland	0.11 (0.08, 0.15)	0.03 (0.02, 0.04)	0.57 (0.30, 0.91)	0.21 (0.10, 0.34)

Location	Incidence (per 100,000)		Mortality (per million)	
	1990	2017	1990	2017
Syria	124.0 (107.3, 143.6)	25.8 (22.3, 29.6)	14.3 (7.5, 24.8)	3.2 (1.7, 5.7)
Taiwan	36.2 (31.4, 41.7)	14.7 (12.6, 16.8)	0.10 (0.05, 0.16)	0.01 (<0.01, 0.02)
Tajikistan	0.18 (0.13, 0.23)	0.13 (0.10, 0.16)	0.02 (0.01, 0.03)	0.01 (0.01, 0.02)
Tanzania	279.9 (220.1, 356.9)	122.1 (97.7, 150.5)	28.3 (9.5, 55.8)	18.7 (9.1, 32.9)
Thailand	751.2 (645.9, 874.6)	279.5 (239.5, 322.7)	54.6 (27.9, 92.3)	20.3 (10.3, 35.2)
The Bahamas	1.7 (1.3, 2.2)	1.4 (1.1, 1.8)	0.09 (0.03, 0.22)	0.07 (0.02, 0.16)
The Gambia	280.9 (241.0, 328.6)	99.5 (85.0, 115.7)	31.7 (16.0, 57.3)	13.7 (6.7, 25.1)
Timor-Leste	436.2 (376.9, 498.7)	159.5 (137.8, 183.3)	46.1 (24.6, 77.2)	20.0 (9.3, 34.7)
Togo	315.2 (269.0, 367.2)	156.1 (134.1, 181.6)	37.2 (19.2, 66.1)	16.5 (8.3, 29.2)
Tonga	295.2 (254.1, 343.8)	83.4 (70.5, 97.7)	19.6 (10.0, 34.4)	6.2 (3.0, 11.3)
Trinidad and Tobago	0.10 (0.07, 0.13)	0.08 (0.06, 0.10)	0.75 (0.42, 0.97)	0.01 (<0.01, 0.03)
Tunisia	116.9 (100.6, 135.1)	30.9 (26.8, 35.7)	8.4 (4.1, 14.8)	2.2 (1.0, 4.1)
Turkey	125.6 (108.8, 145.6)	30.4 (26.3, 35.4)	10.2 (5.1, 18.5)	2.5 (1.2, 4.5)
Turkmenistan	0.24 (0.18, 0.32)	0.13 (0.10, 0.17)	0.02 (0.01, 0.03)	0.01 (<0.01, 0.02)
Uganda	203.7 (174.5, 237.3)	112.4 (96.8, 130.6)	15.4 (7.3, 28.6)	12.4 (6.5, 21.7)
Ukraine	0.53 (0.41, 0.67)	0.53 (0.41, 0.67)	<0.01 (<0.01, 0.01)	<0.01 (<0.01, <0.01)
United Arab Emirates	99.6 (86.3, 114.8)	22.9 (19.8, 26.4)	9.0 (3.1, 19.4)	2.1 (0.7, 4.6)
United Kingdom	0.58 (0.46, 0.73)	0.65 (0.49, 0.86)	0.01 (0.01, 0.02)	<0.01 (<0.01, <0.01)
United States	1.7 (1.5, 2.0)	1.0 (0.8, 1.3)	0.06 (0.02, 0.07)	0.01 (<0.01, 0.02)
Uruguay	1.3 (1.0, 1.7)	0.07 (0.05, 0.10)	0.81 (0.57, 1.01)	0.02 (0.01, 0.05)
Uzbekistan	0.12 (0.09, 0.15)	0.09 (0.07, 0.12)	0.01 (0.01, 0.02)	0.01 (<0.01, 0.01)
Vanuatu	403.1 (341.6, 471.6)	132.8 (112.1, 157.6)	40.5 (20.7, 70.4)	14.6 (7.1, 26.5)
Venezuela	0.17 (0.13, 0.22)	0.08 (0.06, 0.11)	1.4 (0.8, 1.8)	0.05 (0.03, 0.08)
Vietnam	424.2 (351.5, 507.5)	154.7 (128.1, 186.0)	38.1 (20.8, 63.6)	14.7 (7.9, 24.8)

Location	Incidence (per 100,000)		Mortality (per million)	
	1990	2017	1990	2017
Virgin Islands, U.S.	1.4 (1.1, 1.8)	1.2 (0.9, 1.6)	0.08 (0.03, 0.19)	0.05 (0.02, 0.13)
Yemen	234.6 (202.1, 271.5)	82.8 (70.5, 96.9)	22.1 (5.5, 41.9)	10.3 (5.3, 18.0)
Zambia	183.1 (158.3, 212.8)	128.2 (109.7, 147.6)	23.1 (9.6, 41.4)	18.6 (9.6, 32.5)
Zimbabwe	4.2 (3.7, 4.9)	3.7 (3.2, 4.3)	0.45 (0.23, 0.79)	0.48 (0.25, 0.86)

Table S10: Years lived with disability (YLDs), years of life lost to premature mortality (YLLs), and disability-adjusted life years (DALYs) due to typhoid and paratyphoid, by country or non-sovereign location, in 2017.

Location	YLDs	YLLs	DALYs
Afghanistan	413<br>(263, 617)	44,744<br>(22,893, 79,404)	45,157<br>(23,302, 79,890)
Albania	0<br>(0, 0)	5<br>(2, 10)	5<br>(2, 10)
Algeria	91<br>(61, 132)	6,525<br>(3,023, 12,095)	6,617<br>(3,115, 12,180)
American Samoa	0<br>(0, 0)	17<br>(8, 32)	17<br>(8, 32)
Andorra	0<br>(0, 0)	0<br>(0, 0)	0<br>(0, 0)
Angola	262<br>(168, 391)	18,742<br>(8,601, 35,730)	19,004<br>(8,882, 35,966)
Antigua and Barbuda	0<br>(0, 0)	0<br>(0, 0)	0<br>(0, 0)
Argentina	0<br>(0, 0)	54<br>(34, 87)	55<br>(34, 87)
Armenia	0<br>(0, 0)	1<br>(1, 2)	1<br>(1, 2)
Australia	0<br>(0, 0)	1<br>(1, 2)	1<br>(1, 2)
Austria	0<br>(0, 0)	1<br>(0, 2)	1<br>(0, 2)
Azerbaijan	0<br>(0, 0)	3<br>(1, 6)	3<br>(2, 6)
Bahrain	3<br>(2, 4)	144<br>(47, 315)	147<br>(50, 318)
Bangladesh	9,788<br>(6,224, 14,781)	924,923<br>(526,219, 1,497,921)	934,711<br>(533,508, 1,508,179)
Barbados	0<br>(0, 0)	1<br>(1, 2)	1<br>(1, 2)
Belarus	0<br>(0, 0)	8<br>(4, 16)	8<br>(4, 16)
Belgium	0<br>(0, 0)	1<br>(1, 2)	1<br>(1, 2)
Belize	0<br>(0, 0)	3<br>(1, 6)	3<br>(1, 6)
Benin	185<br>(119, 278)	17,822<br>(8,732, 32,309)	18,007<br>(8,950, 32,461)
Bermuda	0<br>(0, 0)	0<br>(0, 0)	0<br>(0, 0)
Bhutan	12<br>(8, 18)	1,216<br>(617, 2,208)	1,228<br>(628, 2,220)
Bolivia	2<br>(1, 2)	95<br>(46, 178)	97<br>(48, 181)

Location	YLDs	YLLs	DALYs
Bosnia and Herzegovina	0<br>(0, 0)	4<br>(2, 9)	5<br>(2, 9)
Botswana	1<br>(0, 1)	36<br>(17, 66)	36<br>(18, 66)
Brazil	41<br>(25, 60)	1,352<br>(630, 2,492)	1,393<br>(672, 2,525)
Brunei	0<br>(0, 0)	0<br>(0, 0)	0<br>(0, 0)
Bulgaria	1<br>(0, 1)	1<br>(1, 4)	2<br>(1, 5)
Burkina Faso	984<br>(601, 1,507)	107,723<br>(54,084, 196,953)	108,707<br>(54,840, 197,824)
Burundi	255<br>(159, 393)	24,271<br>(12,436, 43,076)	24,527<br>(12,768, 43,336)
Cambodia	318<br>(203, 473)	22,652<br>(11,654, 37,917)	22,969<br>(11,900, 38,207)
Cameroon	393<br>(250, 584)	43,291<br>(21,522, 80,317)	43,684<br>(21,982, 80,806)
Canada	0<br>(0, 0)	3<br>(2, 9)	3<br>(2, 9)
Cape Verde	3<br>(2, 4)	324<br>(168, 567)	327<br>(171, 569)
Central African Republic	48<br>(31, 71)	3,696<br>(1,750, 6,894)	3,743<br>(1,803, 6,954)
Chad	286<br>(179, 429)	33,728<br>(16,911, 61,096)	34,014<br>(17,117, 61,410)
Chile	2<br>(2, 4)	25<br>(15, 58)	28<br>(17, 60)
China	4,170<br>(2,836, 5,871)	229,742<br>(117,640, 400,088)	233,912<br>(121,902, 404,479)
Colombia	19<br>(12, 27)	108<br>(68, 218)	127<br>(85, 236)
Comoros	8<br>(5, 12)	1,001<br>(518, 1,773)	1,009<br>(527, 1,780)
Congo	30<br>(20, 44)	2,887<br>(1,445, 5,084)	2,918<br>(1,474, 5,121)
Costa Rica	0<br>(0, 0)	2<br>(1, 3)	2<br>(1, 3)
Cote d'Ivoire	262<br>(170, 389)	31,162<br>(15,991, 55,368)	31,424<br>(16,188, 55,615)
Croatia	0<br>(0, 0)	0<br>(0, 1)	0<br>(0, 1)
Cuba	1<br>(0, 1)	2<br>(1, 4)	2<br>(1, 5)
Cyprus	0<br>(0, 0)	0<br>(0, 1)	0<br>(0, 1)

Location	YLDs	YLLs	DALYs
Czech Republic	1<br>(0, 1)	14<br>(2, 30)	15<br>(2, 31)
Democratic Republic of the Congo	831<br>(523, 1,229)	67,812<br>(33,658, 118,414)	68,642<br>(34,378, 119,319)
Denmark	0<br>(0, 0)	17<br>(5, 28)	17<br>(5, 28)
Djibouti	15<br>(10, 22)	1,823<br>(884, 3,392)	1,838<br>(896, 3,408)
Dominica	0<br>(0, 0)	0<br>(0, 1)	0<br>(0, 1)
Dominican Republic	2<br>(1, 3)	84<br>(37, 157)	86<br>(39, 159)
Ecuador	2<br>(1, 4)	86<br>(39, 159)	88<br>(42, 162)
Egypt	234<br>(154, 341)	20,396<br>(10,362, 35,664)	20,630<br>(10,623, 35,881)
El Salvador	1<br>(1, 2)	70<br>(34, 127)	71<br>(35, 128)
Equatorial Guinea	8<br>(5, 12)	372<br>(112, 928)	380<br>(119, 936)
Eritrea	102<br>(64, 152)	12,210<br>(6,362, 22,047)	12,312<br>(6,451, 22,153)
Estonia	0<br>(0, 0)	0<br>(0, 1)	1<br>(0, 1)
Ethiopia	2,029<br>(1,318, 2,991)	208,656<br>(110,644, 360,776)	210,686<br>(112,374, 363,048)
Federated States of Micronesia	1<br>(0, 1)	74<br>(34, 131)	74<br>(34, 131)
Fiji	4<br>(3, 6)	321<br>(154, 588)	325<br>(158, 593)
Finland	0<br>(0, 0)	1<br>(1, 2)	1<br>(1, 2)
France	1<br>(0, 1)	36<br>(24, 52)	37<br>(25, 52)
Gabon	6<br>(4, 8)	372<br>(171, 708)	378<br>(178, 716)
Georgia	0<br>(0, 0)	2<br>(1, 4)	2<br>(1, 4)
Germany	0<br>(0, 0)	27<br>(16, 43)	27<br>(16, 43)
Ghana	705<br>(445, 1,065)	76,989<br>(40,783, 134,376)	77,694<br>(41,430, 135,056)
Greece	0<br>(0, 0)	22<br>(16, 29)	22<br>(16, 29)
Greenland	0<br>(0, 0)	0<br>(0, 0)	0<br>(0, 0)

Location	YLDs	YLLs	DALYs
Grenada	0<br>(0, 0)	0 (0, 1)	0<br>(0, 1)
Guam	1<br>(0, 1)	36<br>(12, 84)	37<br>(13, 85)
Guatemala	40<br>(26, 56)	1,261<br>(780, 1,724)	1,300<br>(818, 1,766)
Guinea	167<br>(106, 245)	18,368<br>(9,195, 33,025)	18,535<br>(9,351, 33,255)
Guinea-Bissau	6<br>(4, 9)	677<br>(323, 1,268)	682<br>(328, 1,276)
Guyana	0<br>(0, 0)	7<br>(4, 14)	7<br>(4, 14)
Haiti	10<br>(6, 15)	1,186<br>(581, 2,190)	1,196<br>(592, 2,202)
Honduras	2<br>(1, 3)	123<br>(59, 235)	126<br>(61, 237)
Hungary	0<br>(0, 0)	0<br>(0, 1)	1<br>(0, 1)
Iceland	0<br>(0, 0)	0<br>(0, 0)	0<br>(0, 0)
India	62,928<br>(42,391, 88,465)	5,113,164<br>(2,934,073, 8,232,805)	5,176,092<br>(2,996,424, 8,307,312)
Indonesia	6,498<br>(4,337, 9,251)	483,263<br>(271,204, 786,701)	489,762<br>(277,552, 794,178)
Iran	164<br>(110, 231)	9,283<br>(4,559, 16,404)	9,446<br>(4,703, 16,569)
Iraq	224<br>(147, 329)	13,900<br>(6,689, 24,890)	14,123<br>(6,890, 25,104)
Ireland	0<br>(0, 0)	0<br>(0, 0)	0<br>(0, 0)
Israel	0<br>(0, 0)	2<br>(1, 6)	2<br>(1, 6)
Italy	0<br>(0, 0)	12<br>(7, 18)	12<br>(8, 18)
Jamaica	0<br>(0, 0)	12<br>(5, 24)	12<br>(6, 24)
Japan	3<br>(2, 4)	9<br>(7, 23)	12<br>(9, 26)
Jordan	25<br>(16, 36)	1,500<br>(731, 2,782)	1,525<br>(750, 2,802)
Kazakhstan	0<br>(0, 0)	49<br>(11, 94)	49<br>(11, 94)
Kenya	1,015<br>(673, 1,475)	91,761<br>(49,800, 153,404)	92,776<br>(50,650, 154,652)
Kiribati	1<br>(1, 2)	169<br>(88, 300)	170<br>(89, 302)

Location	YLDs	YLLs	DALYs
Kuwait	4<br>(3, 6)	0<br>(0, 1)	5<br>(3, 7)
Kyrgyzstan	0<br>(0, 0)	3<br>(2, 6)	3<br>(2, 6)
Laos	148<br>(93, 217)	14,561<br>(7,643, 24,995)	14,709<br>(7,787, 25,113)
Latvia	0<br>(0, 0)	0<br>(0, 0)	0<br>(0, 1)
Lebanon	26<br>(17, 38)	1,712<br>(811, 3,090)	1,738<br>(837, 3,118)
Lesotho	1<br>(0, 1)	70<br>(36, 128)	71<br>(36, 129)
Liberia	76<br>(48, 115)	6,818<br>(3,367, 12,198)	6,894<br>(3,454, 12,250)
Libya	14<br>(9, 20)	890<br>(421, 1,594)	904<br>(436, 1,610)
Lithuania	0<br>(0, 0)	0<br>(0, 1)	1<br>(0, 1)
Luxembourg	0<br>(0, 0)	0<br>(0, 0)	0<br>(0, 0)
Macedonia	0<br>(0, 0)	3<br>(1, 5)	3<br>(1, 5)
Madagascar	392<br>(238, 615)	33,886<br>(16,052, 61,668)	34,278<br>(16,422, 62,361)
Malawi	321<br>(203, 478)	32,588<br>(16,780, 58,200)	32,909<br>(17,118, 58,522)
Malaysia	311<br>(206, 454)	16,845<br>(8,526, 29,579)	17,156<br>(8,789, 29,823)
Maldives	3<br>(2, 5)	147<br>(70, 266)	150<br>(72, 270)
Mali	324<br>(203, 490)	40,974<br>(20,619, 74,154)	41,297<br>(20,940, 74,540)
Malta	0<br>(0, 0)	0<br>(0, 0)	0<br>(0, 0)
Marshall Islands	0<br>(0, 1)	33<br>(16, 61)	34<br>(17, 61)
Mauritania	40<br>(26, 58)	4,983<br>(2,486, 8,676)	5,023<br>(2,521, 8,708)
Mauritius	1<br>(1, 2)	1<br>(0, 3)	2<br>(1, 4)
Mexico	127<br>(82, 183)	2,009<br>(1,113, 5,313)	2,136<br>(1,240, 5,411)
Moldova	0<br>(0, 0)	1<br>(1, 2)	1<br>(1, 2)
Mongolia	0<br>(0, 0)	2<br>(1, 4)	2<br>(1, 4)

Location	YLDs	YLLs	DALYs
Montenegro	0<br>(0, 0)	1<br>(0, 2)	1<br>(0, 2)
Morocco	120<br>(80, 173)	12,143<br>(6,278, 21,037)	12,263<br>(6,393, 21,140)
Mozambique	704<br>(438, 1,073)	90,599<br>(45,876, 161,836)	91,303<br>(46,692, 162,689)
Myanmar	1,239<br>(785, 1,838)	120,518<br>(65,426, 203,217)	121,757<br>(66,796, 204,182)
Namibia	1<br>(0, 1)	42<br>(19, 79)	43<br>(20, 80)
Nepal	1,131<br>(699, 1,670)	89,741<br>(46,551, 150,471)	90,873<br>(47,712, 151,429)
Netherlands	0<br>(0, 0)	1<br>(1, 2)	1<br>(1, 2)
New Zealand	0<br>(0, 0)	0<br>(0, 0)	0<br>(0, 0)
Nicaragua	2<br>(1, 3)	94<br>(46, 182)	96<br>(48, 184)
Niger	333<br>(210, 496)	33,103<br>(16,395, 60,093)	33,436<br>(16,688, 60,467)
Nigeria	2,824<br>(1,803, 4,236)	322,238<br>(162,128, 592,050)	325,062<br>(164,766, 594,478)
North Korea	39<br>(26, 55)	4,137<br>(2,158, 7,434)	4,175<br>(2,202, 7,468)
Northern Mariana Islands	0<br>(0, 0)	7<br>(2, 17)	7<br>(3, 17)
Norway	0<br>(0, 0)	7<br>(2, 10)	8<br>(3, 10)
Oman	11<br>(8, 16)	571<br>(188, 1,281)	582<br>(200, 1,291)
Pakistan	5,615<br>(3,567, 8,239)	608,497<br>(321,247, 1,053,596)	614,112<br>(326,240, 1,058,525)
Palestine	30<br>(20, 45)	2,687<br>(1,414, 4,675)	2,718<br>(1,437, 4,694)
Panama	1<br>(0, 1)	25<br>(11, 47)	26<br>(12, 48)
Papua New Guinea	141<br>(87, 210)	13,379<br>(6,788, 23,708)	13,520<br>(6,955, 23,857)
Paraguay	1<br>(1, 2)	57<br>(28, 107)	58<br>(29, 108)
Peru	5<br>(3, 7)	160<br>(74, 300)	164<br>(79, 305)
Philippines	1,486<br>(954, 2,122)	25,046<br>(13,609, 49,038)	26,532<br>(15,203, 50,738)
Poland	0<br>(0, 0)	7<br>(4, 18)	7<br>(4, 18)

Location	YLDs	YLLs	DALYs
Portugal	0<br>(0, 0)	7<br>(2, 11)	7<br>(2, 11)
Puerto Rico	0<br>(0, 0)	3<br>(2, 6)	3<br>(2, 6)
Qatar	5<br>(3, 7)	222<br>(73, 490)	227<br>(78, 494)
Romania	1<br>(1, 2)	19<br>(13, 27)	20<br>(14, 28)
Russia	5<br>(3, 8)	19<br>(9, 34)	25<br>(14, 39)
Rwanda	163<br>(106, 240)	18,055<br>(9,308, 32,462)	18,218<br>(9,499, 32,627)
Saint Lucia	0<br>(0, 0)	0<br>(0, 0)	0<br>(0, 0)
Saint Vincent and the Grenadines	0<br>(0, 0)	0<br>(0, 0)	0<br>(0, 0)
Samoa	1<br>(1, 2)	126<br>(62, 223)	127<br>(63, 224)
Sao Tome and Principe	2<br>(1, 3)	258<br>(129, 460)	260<br>(131, 462)
Saudi Arabia	85<br>(57, 123)	4,064<br>(1,349, 9,009)	4,150<br>(1,430, 9,095)
Senegal	123<br>(80, 182)	16,447<br>(8,448, 28,661)	16,570<br>(8,575, 28,798)
Serbia	0<br>(0, 0)	12<br>(5, 24)	12<br>(6, 25)
Seychelles	0<br>(0, 0)	11<br>(5, 20)	11<br>(5, 20)
Sierra Leone	131<br>(83, 201)	13,223<br>(6,602, 24,032)	13,355<br>(6,741, 24,170)
Singapore	0<br>(0, 0)	1<br>(0, 1)	1<br>(0, 1)
Slovakia	0<br>(0, 0)	6<br>(2, 16)	6<br>(2, 17)
Slovenia	0<br>(0, 0)	0<br>(0, 1)	0<br>(0, 1)
Solomon Islands	7<br>(5, 11)	698<br>(366, 1,203)	705<br>(373, 1,210)
Somalia	298<br>(185, 450)	26,472<br>(12,967, 49,210)	26,770<br>(13,240, 49,590)
South Africa	9<br>(6, 13)	559<br>(263, 1,023)	568<br>(272, 1,034)
South Korea	0<br>(0, 0)	15<br>(4, 32)	15<br>(4, 32)
South Sudan	243<br>(149, 367)	23,742<br>(11,481, 42,678)	23,985<br>(11,655, 42,891)

Location	YLDs	YLLs	DALYs
Spain	1<br>(0, 1)	15<br>(10, 27)	16<br>(11, 27)
Sri Lanka	293<br>(193, 427)	21,317<br>(11,691, 35,120)	21,610<br>(11,943, 35,530)
Sudan	314<br>(204, 464)	30,142<br>(15,268, 53,597)	30,456<br>(15,593, 53,874)
Suriname	0<br>(0, 0)	3<br>(1, 5)	3<br>(1, 5)
Swaziland	0<br>(0, 1)	39<br>(20, 73)	39<br>(20, 73)
Sweden	0<br>(0, 0)	2<br>(1, 3)	2<br>(1, 3)
Switzerland	0<br>(0, 0)	57<br>(32, 94)	57<br>(32, 94)
Syria	53<br>(34, 77)	5,059<br>(2,626, 9,064)	5,112<br>(2,673, 9,124)
Taiwan	20<br>(14, 28)	6<br>(3, 14)	26<br>(18, 38)
Tajikistan	0<br>(0, 0)	5<br>(2, 8)	5<br>(2, 8)
Tanzania	782<br>(475, 1,213)	101,630<br>(47,396, 185,513)	102,412<br>(48,039, 186,630)
Thailand	1,369<br>(897, 1,964)	70,071<br>(35,532, 121,491)	71,440<br>(36,927, 122,438)
The Bahamas	0<br>(0, 0)	1 (0, 3)	1<br>(0, 3)
The Gambia	23<br>(15, 34)	2,798<br>(1,316, 5,282)	2,821<br>(1,332, 5,303)
Timor-Leste	21<br>(13, 33)	2,307<br>(1,059, 4,067)	2,329<br>(1,086, 4,084)
Togo	123<br>(78, 183)	10,772<br>(5,362, 19,017)	10,894<br>(5,476, 19,107)
Tonga	1<br>(1, 1)	51<br>(24, 94)	51<br>(25, 94)
Trinidad and Tobago	0<br>(0, 0)	0<br>(0, 1)	0<br>(0, 1)
Tunisia	32<br>(21, 45)	1,540<br>(723, 2,824)	1,572<br>(756, 2,862)
Turkey	222<br>(147, 321)	12,293<br>(5,794, 22,268)	12,515<br>(6,012, 22,477)
Turkmenistan	0<br>(0, 0)	2<br>(1, 3)	2<br>(1, 3)
Uganda	553<br>(352, 823)	50,282<br>(25,210, 89,166)	50,835<br>(25,668, 89,650)
Ukraine	1<br>(1, 2)	2<br>(1, 7)	4<br>(2, 8)

Location	YLDs	YLLs	DALYs
United Arab Emirates	16<br>(11, 23)	923<br>(303, 2,043)	940<br>(323, 2,060)
United Kingdom	2<br>(1, 2)	7<br>(3, 11)	8<br>(5, 13)
United States	16<br>(10, 23)	99<br>(64, 282)	115<br>(77, 297)
Uruguay	0<br>(0, 0)	2<br>(1, 6)	2<br>(1, 6)
Uzbekistan	0<br>(0, 0)	11<br>(5, 19)	11<br>(6, 20)
Vanuatu	4<br>(2, 5)	356<br>(174, 654)	360<br>(177, 658)
Venezuela	0<br>(0, 0)	47<br>(29, 78)	47<br>(30, 78)
Vietnam	1,211<br>(771, 1,791)	86,378<br>(46,096, 145,920)	87,589<br>(47,458, 146,947)
Virgin Islands, U.S.	0<br>(0, 0)	0<br>(0, 1)	0<br>(0, 1)
Yemen	290<br>(187, 430)	27,776<br>(14,151, 48,844)	28,066<br>(14,412, 49,024)
Zambia	271<br>(171, 409)	31,859<br>(16,367, 56,427)	32,129<br>(16,594, 56,670)
Zimbabwe	6<br>(4, 9)	656<br>(342, 1,191)	663<br>(348, 1,197)

C. Data sources

Incidence data

Argentina

1. Directorate of Epidemiology, Ministry of Health (Argentina). Argentina Annual Epidemiological Bulletin 2003-2005. Buenos Aires, Argentina: Ministry of Health (Argentina).

Armenia

2. Ministry of Health (Armenia), National Statistical Service of the Republic of Armenia. Armenia Health and Health Care 2009. Yerevan, Armenia: National Health Information Analytic Center, National Institute of Health (Armenia), 2010.

Australia

3. OzFoodNet Working Group. Monitoring the incidence and causes of diseases potentially transmitted by food in Australia: annual report of the OzFoodNet Network, 2009. Commun Dis Intell Q Rep. 2010; 34(4): 396-426.

Bangladesh

4. Brooks WA, Hossain A, Goswami D, Sharmeen AT, Nahar K, Alam K, Ahmed N, Naheed A, Nair GB, Luby S, Breiman RF. Bacteremic Typhoid Fever in Children in an Urban Slum, Bangladesh. Emerg Infect Dis. 2005; 11(2): 326-9.
5. Dewan AM, Corner R, Hashizume M, Ongee ET. Typhoid Fever and its association with environmental factors in the Dhaka Metropolitan Area of Bangladesh: a spatial and time-series approach. PLoS Negl Trop Dis. 2013; 7(1): e1998.
6. Naheed A, Ram PK, Brooks WA, Hossain MA, Parsons MB, Talukder KA, Mintz E, Luby S, Breiman RF. Burden of typhoid and paratyphoid fever in a densely populated urban community, Dhaka, Bangladesh. Int J Infect Dis. 2010; 14(Suppl 3): e93-e99.

Burkina Faso

7. International Vaccine Institute. Typhoid Fever Surveillance in Africa Program (TSAP) Typhoid & Salmonella Tabulations.

Chile

8. Black RE, Levine MM, Ferreccio C, Clements ML, Lanata C, Rooney J, Germanier R, Chilean Typhoid Committee. Efficacy of one or two doses of Ty21a Salmonella typhi vaccine in enteric-coated capsules in a controlled field trial. Vaccine. 1990; 8(1): 81-4.
9. Levine MM, Ferreccio C, Black RE, Germanier R. Large-scale field trial of Ty21a live oral typhoid vaccine in enteric-coated capsule formulation. Lancet. 1987; 1(8541): 1049-52.

10. Levine MM, Ferreccio C, Cryz S, Ortiz E. Comparison of enteric-coated capsules and liquid formulation of Ty21a typhoid vaccine in randomised controlled field trial. Lancet. 1990; 336(8720): 891-4.
11. Ministry of Health (Chile). Chile Quarterly Epidemiological Bulletin, January to March 2015. Santiago, Chile: Ministry of Health (Chile), 2015.

China

12. Chinese Center for Disease Control and Prevention (CCDC). China Notifiable Infectious Diseases 2004-2012.
13. Ochiai RL, Acosta CJ, Danovaro-Holliday MC, Baiqing D, Bhattacharya SK, Agtini MD, Bhutta ZA, Canh DG, Ali M, Shin S, Wain J, Page A-L, Albert MJ, Farrar J, Abu-Elyazeed R, Pang T, Galindo CM, von Seidlein L, Clemens JD, Domi Typhoid Study Group. A study of typhoid fever in five Asian countries: disease burden and implications for controls. Bull World Health Organ. 2008; 86(4): 260-8.
14. Ochiai RL, Wang X, von Seidlein L, Yang J, Bhutta ZA, Bhattacharya SK, Agtini M, Deen JL, Wain J, Kim DR, Ali M, Acosta CJ, Jodar L, Clemens JD. Salmonella Paratyphi A Rates, Asia. Emerg Infect Dis. 2005; 11(11): 1764-6.

Cuba

15. National Office of Statistics (Cuba). Cuba Statistical Yearbook 2002. Havana, Cuba: National Office of Statistics (Cuba), 2003

Egypt

16. Crump JA, Youssef FG, Luby SP, Wasfy MO, Rangel JM, Taalat M, Oun SA, Mahoney FJ. Estimating the Incidence of Typhoid Fever and Other Febrile Illnesses in Developing Countries. Emerg Infect Dis. 2003; 9(5): 539-44.
17. Srikantiah P, Girgis FY, Luby SP, Jennings G, Wasfy MO, Crump JA, Hoekstra RM, Anwer M, Mahoney FJ. Population-based surveillance of typhoid fever in Egypt. Am J Trop Med Hyg. 2006; 74(1): 114Ã¢Â€Â“119.
18. Wahdan MH, SÃƒÂ©riÃƒÂ© C, Cerisier Y, Sallam S, Germanier R. A controlled field trial of live Salmonella typhi strain Ty 21a oral vaccine against typhoid: three-year results. J Infect Dis. 1982; 145(3): 292-5.

Germany

19. Robert Koch Institute. Germany Epidemiological Infection Yearbook for Notifiable Diseases 2001. Berlin, Germany: Robert Koch Institute, 2002.
20. Robert Koch Institute. Germany Epidemiological Infection Yearbook for Notifiable Diseases 2002. Berlin, Germany: Robert Koch Institute, 2003.
21. Robert Koch Institute. Germany Epidemiological Infection Yearbook for Notifiable Diseases 2003. Berlin, Germany: Robert Koch Institute, 2004.
22. Robert Koch Institute. Germany Epidemiological Infection Yearbook for Notifiable Diseases 2004. Berlin, Germany: Robert Koch Institute, 2005.
23. Robert Koch Institute. Germany Epidemiological Infection Yearbook for Notifiable Diseases 2005. Berlin, Germany: Robert Koch Institute, 2006.

24. Robert Koch Institute. Germany Epidemiological Infection Yearbook for Notifiable Diseases 2007. Berlin, Germany: Robert Koch Institute, 2008.
25. Robert Koch Institute. Germany Epidemiological Infection Yearbook for Notifiable Diseases 2008. Berlin, Germany: Robert Koch Institute, 2009.
26. Robert Koch Institute. Germany Epidemiological Infection Yearbook for Notifiable Diseases 2009. Berlin, Germany: Robert Koch Institute, 2010.
27. Robert Koch Institute. Germany Epidemiological Infection Yearbook for Notifiable Diseases 2010. Berlin, Germany: Robert Koch Institute, 2011.
28. Robert Koch Institute. Germany Epidemiological Infection Yearbook for Notifiable Diseases 2011. Berlin, Germany: Robert Koch Institute, 2012.

Ghana

29. International Vaccine Institute. Typhoid Fever Surveillance in Africa Program (TSAP) Typhoid & Salmonella Tabulations.
30. Marks F, Adu-Sarkodie Y, HÃƒÂ¼nger F, Sarpong N, Ekuban S, Agyekum A, Nkrumah B, Schwarz NG, Favorov MO, Meyer CG, May J. Typhoid fever among children, Ghana. Emerg Infect Dis. 2010; 16(11): 1796-7.
31. Nielsen MV, Sarpong N, Krumkamp R, Dekker D, Loag W, Amemasor S, Agyekum A, Marks F, Huenger F, Krefis AC, Hagen RM, Adu-Sarkodie Y, May J, Schwarz NG. Incidence and Characteristics of Bacteremia among Children in Rural Ghana. PLoS One. 2012; 7(9): e44063.

Guinea-Bissau

32. International Vaccine Institute. Typhoid Fever Surveillance in Africa Program (TSAP) Typhoid & Salmonella Tabulations.

India

33. Ochiai RL, Acosta CJ, Danovaro-Holliday MC, Baiqing D, Bhattacharya SK, Agtini MD, Bhutta ZA, Canh DG, Ali M, Shin S, Wain J, Page A-L, Albert MJ, Farrar J, Abu-Elyazeed R, Pang T, Galindo CM, von Seidlein L, Clemens JD, Domi Typhoid Study Group. A study of typhoid fever in five Asian countries: disease burden and implications for controls. Bull World Health Organ. 2008; 86(4): 260-8.
34. Ochiai RL, Wang X, von Seidlein L, Yang J, Bhutta ZA, Bhattacharya SK, Agtini M, Deen JL, Wain J, Kim DR, Ali M, Acosta CJ, Jodar L, Clemens JD. Salmonella Paratyphi A Rates, Asia. Emerg Infect Dis. 2005; 11(11): 1764-6.
35. Sinha A, Sazawal S, Kumar R, Sood S, Reddaiah VP, Singh B, Rao M, Naficy A, Clemens JD, Bhan MK. Typhoid fever in children aged less than 5 years. Lancet. 1999; 354(9180): 734-7.
36. Sur D, Ali M, von Seidlein L, Manna B, Deen JL, Acosta CJ, Clemens JD, Bhattacharya SK. Comparisons of predictors for typhoid and paratyphoid fever in Kolkata, India. BMC Public Health. 2007; 7: 289.
37. Sur D, Ochiai RL, Bhattacharya SK, Ganguly NK, Ali M, Manna B, Dutta S, Donner A, Kanungo S, Park JK, Puri MK, Kim DR, Dutta D, Bhaduri B, Acosta CJ, Clemens JD. A cluster-randomized effectiveness trial of Vi typhoid vaccine in India. N Engl J Med. 2009; 361(4): 335-44.

Indonesia

38. Ochiai RL, Acosta CJ, Danovaro-Holliday MC, Baiqing D, Bhattacharya SK, Agtini MD, Bhutta ZA, Canh DG, Ali M, Shin S, Wain J, Page A-L, Albert MJ, Farrar J, Abu-Elyazeed R, Pang T, Galindo CM, von Seidlein L, Clemens JD, Domi Typhoid Study Group. A study of typhoid fever in five Asian countries: disease burden and implications for controls. Bull World Health Organ. 2008; 86(4): 260-8.
39. Ochiai RL, Wang X, von Seidlein L, Yang J, Bhutta ZA, Bhattacharya SK, Agtini M, Deen JL, Wain J, Kim DR, Ali M, Acosta CJ, Jodar L, Clemens JD. Salmonella Paratyphi A Rates, Asia. Emerg Infect Dis. 2005; 11(11): 1764-6.
40. Punjabi NH, Agtini MD, Ochiai RL, Simanjuntak CH, Lesmana M, Subekti D, Oyofo BA, Seidlein L von, Deen J, Shin S, Acosta C, Wangsasaputra F, Pulungsih SP, Saroso S, Suyeti S, R S, Sudarmono P, Syarurachman A, Suwandono A, Arjoso S, Iii HJB, Corwin AL, Clemens JD. Enteric fever burden in North Jakarta, Indonesia: a prospective, community-based study. J Infect Dev Ctries. 2013; 7(11): 781Ã¢Â€Â“7.
41. Simanjuntak CH, Totosudirjo H, Haryanto P, Suprijanto E, Paleologo FP, Punjabi NH, Witham ND, Darmowigoto R, Soeprawoto, Hoffman SL. Oral immunisation against typhoid fever in Indonesia with Ty21a vaccine. Lancet. 1991; 338(8774): 1055-9.

Kenya

42. Breiman RF, Cosmas L, Njuguna H, Audi A, Olack B, Ochieng JB, Wamola N, Bigogo GM, Awiti G, Tabu CW, Burke H, Williamson J, Oundo JO, Mintz ED, Feikin DR. Population-based incidence of typhoid fever in an urban informal settlement and a rural area in Kenya: implications for typhoid vaccine use in Africa. PLoS One. 2012; 7(1): e29119.
43. International Vaccine Institute. Typhoid Fever Surveillance in Africa Program (TSAP) Typhoid & Salmonella Tabulations.

Madagascar

44. International Vaccine Institute. Typhoid Fever Surveillance in Africa Program (TSAP) Typhoid & Salmonella Tabulations.

Nepal

45. Karkey A, Arjyal A, Anders KL, Boni MF, Dongol S, Koirala S, My PVT, Nga TVT, Clements ACA, Holt KE, Duy PT, Day JN, Campbell JI, Dougan G, Dolecek C, Farrar J, Basnyat B, Baker S. The Burden and Characteristics of Enteric Fever at a Healthcare Facility in a Densely Populated Area of Kathmandu. PLoS One. 2010; 5(11).

New Zealand

46. Institute of Environmental Science & Research Limited (New Zealand). New Zealand Notifiable and Other Diseases Annual Surveillance Report 2009. Wellington, New Zealand: Institute of Environmental Science & Research Limited (New Zealand), 2010.

Pakistan

47. Khan MI, Ochiai RL, Soofi SB, Von-Seidlein L, Khan MJ, Sahito SM, Habib MA, Puri MK, Park JK, You YA, Ali M, Nizami SQ, Acosta CJ, Bradley-Sack R, Clemens JD, Bhutta ZA. Risk factors associated

with typhoid fever in children aged 2-16 years in Karachi, Pakistan. Epidemiol Infect. 2012; 140(4): 665-72.
48. Khan MI, Sahito SM, Khan MJ, Wassan SM, Shaikh AW, Maheshwari AK, Acosta CJ, Galindo CM, Ochiai RL, Rasool S, Peerwani S, Puri MK, Ali M, Zafar A, Hassan R, von Seidlein L, Clemens JD, Nizami SQ, Bhutta ZA. Enhanced disease surveillance through private health care sector cooperation in Karachi, Pakistan: experience from a vaccine trial. Bull World Health Organ. 2006; 84(1): 72-7.
49. Khan MI, Soofi SB, Ochiai RL, Khan MJ, Sahito SM, Habib MA, Puri MK, Von Seidlein L, Park JK, You YA, Ali M, Nizami SQ, Acosta CJ, Sack RB, Clemens JD, Bhutta ZA. Epidemiology, clinical presentation, and patterns of drug resistance of Salmonella Typhi in Karachi, Pakistan. J Infect Dev Ctries. 2012; 6(10): 704-14.
50. Ochiai RL, Acosta CJ, Danovaro-Holliday MC, Baiqing D, Bhattacharya SK, Agtini MD, Bhutta ZA, Canh DG, Ali M, Shin S, Wain J, Page A-L, Albert MJ, Farrar J, Abu-Elyazeed R, Pang T, Galindo CM, von Seidlein L, Clemens JD, Domi Typhoid Study Group. A study of typhoid fever in five Asian countries: disease burden and implications for controls. Bull World Health Organ. 2008; 86(4): 260-8.
51. Ochiai RL, Wang X, von Seidlein L, Yang J, Bhutta ZA, Bhattacharya SK, Agtini M, Deen JL, Wain J, Kim DR, Ali M, Acosta CJ, Jodar L, Clemens JD. Salmonella Paratyphi A Rates, Asia. Emerg Infect Dis. 2005; 11(11): 1764-6.
52. Owais A, Sultana S, Zaman U, Rizvi A, Zaidi AKM. Incidence of typhoid bacteremia in infants and young children in southern coastal Pakistan. Pediatr Infect Dis J. 2010; 29(11): 1035-9.
53. Siddiqui FJ, Rabbani F, Hasan R, Nizami SQ, Bhutta ZA. Typhoid fever in children: some epidemiological considerations from Karachi, Pakistan. Int J Infect Dis. 2006; 10(3): 215-22.

Philippines

54. Department of Health (Philippines). Philippines Health Statistics 2004. Manila, Philippines: Department of Health (Philippines).

South Africa

55. Klugman KP, Koornhof HJ, Robbins JB, Le Cam NN. Immunogenicity, efficacy and serological correlate of protection of Salmonella typhi Vi capsular polysaccharide vaccine three years after immunization. Vaccine. 1996; 14(5): 435Ã¢Â€Â“8.
56. National Institute for Communicable Diseases (South Africa). South Africa GERMS-SA Typhoid and Paratyphoid Tabulations 2003.
57. National Institute for Communicable Diseases (South Africa). South Africa GERMS-SA Typhoid and Paratyphoid Tabulations 2004.
58. National Institute for Communicable Diseases (South Africa). South Africa GERMS-SA Typhoid and Paratyphoid Tabulations 2005.
59. National Institute for Communicable Diseases (South Africa). South Africa GERMS-SA Typhoid and Paratyphoid Tabulations 2006.
60. National Institute for Communicable Diseases (South Africa). South Africa GERMS-SA Typhoid and Paratyphoid Tabulations 2007.
61. National Institute for Communicable Diseases (South Africa). South Africa GERMS-SA Typhoid and Paratyphoid Tabulations 2008.

62. National Institute for Communicable Diseases (South Africa). South Africa GERMS-SA Typhoid and Paratyphoid Tabulations 2009.
63. National Institute for Communicable Diseases (South Africa). South Africa GERMS-SA Typhoid and Paratyphoid Tabulations 2010.
64. National Institute for Communicable Diseases (South Africa). South Africa GERMS-SA Typhoid and Paratyphoid Tabulations 2011.
65. National Institute for Communicable Diseases (South Africa). South Africa GERMS-SA Typhoid and Paratyphoid Tabulations 2012.
66. National Institute for Communicable Diseases (South Africa). South Africa GERMS-SA Typhoid and Paratyphoid Tabulations 2013.
67. National Institute for Communicable Diseases (South Africa). South Africa GERMS-SA Typhoid and Paratyphoid Tabulations 2014.

Tanzania

68. International Vaccine Institute. Typhoid Fever Surveillance in Africa Program (TSAP) Typhoid & Salmonella Tabulations.

Tonga

69. Lutui T, 'Ofanoa M, Finau SA, Veikune MK. Typhoid fever in Tonga. Pac Health Dialog. 1999; 6(2): 240-4.

United States

70. Adamson R, Reddy V, Jones L, Antwi M, Bregman B, Weiss D, Phillips M, Horowitz HW. Epidemiology and burden of hepatitis A, malaria, and typhoid in New York City associated with travel: implications for public health policy. Am J Public Health. 2010; 100(7): 1249-52.

Vietnam

71. Lin FY, Ho VA, Khiem HB, Trach DD, Bay PV, Thanh TC, Kossaczka Z, Bryla DA, Shiloach J, Robbins JB, Schneerson R, Szu SC. The efficacy of a Salmonella typhi Vi conjugate vaccine in two-to-five-year-old children. N Engl J Med. 2001; 344(17): 1263-9.
72. Lin FY, Vo AH, Phan VB, Nguyen TT, Bryla D, Tran CT, Ha BK, Dang DT, Robbins JB. The epidemiology of typhoid fever in the Dong Thap Province, Mekong Delta region of Vietnam. Am J Trop Med Hyg. 2000; 62(5): 644Ã¢Â€Â“648.
73. Ochiai RL, Acosta CJ, Danovaro-Holliday MC, Baiqing D, Bhattacharya SK, Agtini MD, Bhutta ZA, Canh DG, Ali M, Shin S, Wain J, Page A-L, Albert MJ, Farrar J, Abu-Elyazeed R, Pang T, Galindo CM, von Seidlein L, Clemens JD, Domi Typhoid Study Group. A study of typhoid fever in five Asian countries: disease burden and implications for controls. Bull World Health Organ. 2008; 86(4): 260-8.

Vital registration data

Antigua and Barbuda

1. World Health Organization (WHO). WHO Mortality Database Version September 2016. Geneva, Switzerland: World Health Organization (WHO), 2016.
2. World Health Organization (WHO). WHO Mortality Database Version October 2017. Geneva, Switzerland: World Health Organization (WHO), 2017.

Argentina

3. World Health Organization (WHO). WHO Mortality Database Version September 2016. Geneva, Switzerland: World Health Organization (WHO), 2016.
4. World Health Organization (WHO). WHO Mortality Database Version October 2017. Geneva, Switzerland: World Health Organization (WHO), 2017.
5. World Health Organization (WHO). WHO Mortality Database Version April 2018. Geneva, Switzerland: World Health Organization (WHO), 2018.

Australia

6. Australian Bureau of Statistics. Australia Vital Registration - Deaths 2005.
7. World Health Organization (WHO). WHO Mortality Database Version November 2015. Geneva, Switzerland: World Health Organization (WHO), 2015.
8. World Health Organization (WHO). WHO Mortality Database Version October 2017. Geneva, Switzerland: World Health Organization (WHO), 2017.

Austria

9. World Health Organization (WHO). WHO Mortality Database Version November 2015. Geneva, Switzerland: World Health Organization (WHO), 2015.
10. World Health Organization (WHO). WHO Mortality Database Version September 2016. Geneva, Switzerland: World Health Organization (WHO), 2016.

Barbados

11. World Health Organization (WHO). WHO Mortality Database Version September 2016. Geneva, Switzerland: World Health Organization (WHO), 2016.

Belgium

12. World Health Organization (WHO). WHO Mortality Database Version March 2017. Geneva, Switzerland: World Health Organization (WHO), 2017.
13. World Health Organization (WHO). WHO Mortality Database Version November 2015. Geneva, Switzerland: World Health Organization (WHO), 2015.
14. World Health Organization (WHO). WHO Mortality Database Version September 2016. Geneva, Switzerland: World Health Organization (WHO), 2016.

Bermuda

15. World Health Organization (WHO). WHO Mortality Database Version September 2016. Geneva, Switzerland: World Health Organization (WHO), 2016.
16. World Health Organization (WHO). WHO Mortality Database Version October 2017. Geneva, Switzerland: World Health Organization (WHO), 2017.

Belgium

17. World Health Organization (WHO). WHO Mortality Database Version November 2015. Geneva, Switzerland: World Health Organization (WHO), 2015.
18. World Health Organization (WHO). WHO Mortality Database Version September 2016. Geneva, Switzerland: World Health Organization (WHO), 2016.
19. World Health Organization (WHO). WHO Mortality Database Version March 2017. Geneva, Switzerland: World Health Organization (WHO), 2017.
20. World Health Organization (WHO). WHO Mortality Database Version October 2017. Geneva, Switzerland: World Health Organization (WHO), 2017.
21. World Health Organization (WHO). WHO Mortality Database Version April 2018. Geneva, Switzerland: World Health Organization (WHO), 2018.

Bermuda

22. World Health Organization (WHO). WHO Mortality Database Version September 2016. Geneva, Switzerland: World Health Organization (WHO), 2016.
23. World Health Organization (WHO). WHO Mortality Database Version October 2017. Geneva, Switzerland: World Health Organization (WHO), 2017.

Bulgaria

24. World Health Organization (WHO). WHO Mortality Database Version November 2015. Geneva, Switzerland: World Health Organization (WHO), 2015.
25. World Health Organization (WHO). WHO Mortality Database Version September 2016. Geneva, Switzerland: World Health Organization (WHO), 2016.
26. World Health Organization (WHO). WHO Mortality Database Version October 2017. Geneva, Switzerland: World Health Organization (WHO), 2017.

Canada

27. World Health Organization (WHO). WHO Mortality Database Version November 2015. Geneva, Switzerland: World Health Organization (WHO), 2015.
28. World Health Organization (WHO). WHO Mortality Database Version September 2016. Geneva, Switzerland: World Health Organization (WHO), 2016.
29. World Health Organization (WHO). WHO Mortality Database Version March 2017. Geneva, Switzerland: World Health Organization (WHO), 2017.
30. World Health Organization (WHO). WHO Mortality Database Version October 2017. Geneva, Switzerland: World Health Organization (WHO), 2017.

Chile

31. Department of Statistics and Health Information, Ministry of Health (Chile), National Institute of Statistics (Chile). Chile Vital Statistics - Deaths 1990.
32. Department of Statistics and Health Information, Ministry of Health (Chile), National Institute of Statistics (Chile). Chile Vital Statistics - Deaths 1991.
33. Department of Statistics and Health Information, Ministry of Health (Chile), National Institute of Statistics (Chile). Chile Vital Statistics - Deaths 1992.
34. Department of Statistics and Health Information, Ministry of Health (Chile), National Institute of Statistics (Chile). Chile Vital Statistics - Deaths 1993.

35. Department of Statistics and Health Information, Ministry of Health (Chile), National Institute of Statistics (Chile). Chile Vital Statistics - Deaths 1994.
36. Department of Statistics and Health Information, Ministry of Health (Chile), National Institute of Statistics (Chile). Chile Vital Statistics - Deaths 1995.
37. Department of Statistics and Health Information, Ministry of Health (Chile), National Institute of Statistics (Chile). Chile Vital Statistics - Deaths 1996.
38. World Health Organization (WHO). WHO Mortality Database Version September 2016. Geneva, Switzerland: World Health Organization (WHO), 2016.
39. World Health Organization (WHO). WHO Mortality Database Version October 2017. Geneva, Switzerland: World Health Organization (WHO), 2017.

Colombia

40. National Administrative Department of Statistics (Colombia). Colombia Vital Statistics - Deaths 2008. Bogota, Colombia: National Administrative Department of Statistics (Colombia).
41. World Health Organization (WHO). WHO Mortality Database Version October 2017. Geneva, Switzerland: World Health Organization (WHO), 2017.
42. World Health Organization (WHO). WHO Mortality Database Version April 2018. Geneva, Switzerland: World Health Organization (WHO), 2018.

Costa Rica

43. World Health Organization (WHO). WHO Mortality Database Version September 2016. Geneva, Switzerland: World Health Organization (WHO), 2016.
44. World Health Organization (WHO). WHO Mortality Database Version October 2017. Geneva, Switzerland: World Health Organization (WHO), 2017.

Croatia

45. World Health Organization (WHO). WHO Mortality Database Version November 2015. Geneva, Switzerland: World Health Organization (WHO), 2015.
46. World Health Organization (WHO). WHO Mortality Database Version October 2017. Geneva, Switzerland: World Health Organization (WHO), 2017.
47. World Health Organization (WHO). WHO Mortality Database Version April 2018. Geneva, Switzerland: World Health Organization (WHO), 2018.

Cuba

48. World Health Organization (WHO). WHO Mortality Database Version November 2015. Geneva, Switzerland: World Health Organization (WHO), 2015.
49. World Health Organization (WHO). WHO Mortality Database Version September 2016. Geneva, Switzerland: World Health Organization (WHO), 2016.
50. World Health Organization (WHO). WHO Mortality Database Version October 2017. Geneva, Switzerland: World Health Organization (WHO), 2017.

Czech Republic

51. World Health Organization (WHO). WHO Mortality Database Version November 2015. Geneva, Switzerland: World Health Organization (WHO), 2015.

52. World Health Organization (WHO). WHO Mortality Database Version March 2017. Geneva, Switzerland: World Health Organization (WHO), 2017.
53. World Health Organization (WHO). WHO Mortality Database Version October 2017. Geneva, Switzerland: World Health Organization (WHO), 2017.
54. World Health Organization (WHO). WHO Mortality Database Version April 2018. Geneva, Switzerland: World Health Organization (WHO), 2018.

Denmark

55. World Health Organization (WHO). WHO Mortality Database Version March 2017. Geneva, Switzerland: World Health Organization (WHO), 2017.
56. World Health Organization (WHO). WHO Mortality Database Version October 2017. Geneva, Switzerland: World Health Organization (WHO), 2017.

Estonia

57. World Health Organization (WHO). WHO Mortality Database Version November 2015. Geneva, Switzerland: World Health Organization (WHO), 2015.
58. World Health Organization (WHO). WHO Mortality Database Version October 2017. Geneva, Switzerland: World Health Organization (WHO), 2017.

Finland

59. World Health Organization (WHO). WHO Mortality Database Version September 2016. Geneva, Switzerland: World Health Organization (WHO), 2016.
60. World Health Organization (WHO). WHO Mortality Database Version October 2017. Geneva, Switzerland: World Health Organization (WHO), 2017.

France

61. World Health Organization (WHO). WHO Mortality Database Version November 2015. Geneva, Switzerland: World Health Organization (WHO), 2015.
62. World Health Organization (WHO). WHO Mortality Database Version October 2017. Geneva, Switzerland: World Health Organization (WHO), 2017.

Germany

63. World Health Organization (WHO). WHO Mortality Database Version November 2015. Geneva, Switzerland: World Health Organization (WHO), 2015.
64. World Health Organization (WHO). WHO Mortality Database Version September 2016. Geneva, Switzerland: World Health Organization (WHO), 2016.
65. World Health Organization (WHO). WHO Mortality Database Version October 2017. Geneva, Switzerland: World Health Organization (WHO), 2017.

Greece

66. World Health Organization (WHO). WHO Mortality Database Version November 2015. Geneva, Switzerland: World Health Organization (WHO), 2015.
67. World Health Organization (WHO). WHO Mortality Database Version September 2016. Geneva, Switzerland: World Health Organization (WHO), 2016.

68. World Health Organization (WHO). WHO Mortality Database Version March 2017. Geneva, Switzerland: World Health Organization (WHO), 2017.
69. World Health Organization (WHO). WHO Mortality Database Version April 2018. Geneva, Switzerland: World Health Organization (WHO), 2018.

Guatemala

70. World Health Organization (WHO). WHO Mortality Database Version November 2015. Geneva, Switzerland: World Health Organization (WHO), 2015.
71. World Health Organization (WHO). WHO Mortality Database Version September 2016. Geneva, Switzerland: World Health Organization (WHO), 2016.
72. World Health Organization (WHO). WHO Mortality Database Version October 2017. Geneva, Switzerland: World Health Organization (WHO), 2017.

Hungary

73. World Health Organization (WHO). WHO Mortality Database Version November 2015. Geneva, Switzerland: World Health Organization (WHO), 2015.
74. World Health Organization (WHO). WHO Mortality Database Version October 2017. Geneva, Switzerland: World Health Organization (WHO), 2017.
75. World Health Organization (WHO). WHO Mortality Database Version March 2017. Geneva, Switzerland: World Health Organization (WHO), 2017.
76. World Health Organization (WHO). WHO Mortality Database Version April 2018. Geneva, Switzerland: World Health Organization (WHO), 2018.

Iceland

77. Directorate of Health (Iceland). Iceland Causes of Death Register 2017.
78. World Health Organization (WHO). WHO Mortality Database Version November 2015. Geneva, Switzerland: World Health Organization (WHO), 2015.
79. World Health Organization (WHO). WHO Mortality Database Version September 2016. Geneva, Switzerland: World Health Organization (WHO), 2016.
80. World Health Organization (WHO). WHO Mortality Database Version March 2017. Geneva, Switzerland: World Health Organization (WHO), 2017.
81. World Health Organization (WHO). WHO Mortality Database Version October 2017. Geneva, Switzerland: World Health Organization (WHO), 2017.

Ireland

82. World Health Organization (WHO). WHO Mortality Database Version November 2015. Geneva, Switzerland: World Health Organization (WHO), 2015.
83. World Health Organization (WHO). WHO Mortality Database Version September 2016. Geneva, Switzerland: World Health Organization (WHO), 2016.
84. World Health Organization (WHO). WHO Mortality Database Version October 2017. Geneva, Switzerland: World Health Organization (WHO), 2017.

Israel

85. World Health Organization (WHO). WHO Mortality Database Version November 2015. Geneva, Switzerland: World Health Organization (WHO), 2015.

86. World Health Organization (WHO). WHO Mortality Database Version September 2016. Geneva, Switzerland: World Health Organization (WHO), 2016.
87. World Health Organization (WHO). WHO Mortality Database Version March 2017. Geneva, Switzerland: World Health Organization (WHO), 2017.
88. World Health Organization (WHO). WHO Mortality Database Version October 2017. Geneva, Switzerland: World Health Organization (WHO), 2017.
89. World Health Organization (WHO). WHO Mortality Database Version April 2018. Geneva, Switzerland: World Health Organization (WHO), 2018.

Italy

90. National Institute of Statistics (Italy). Italy Vital Registration - Deaths 2015.
91. World Health Organization (WHO). WHO Mortality Database Version November 2015. Geneva, Switzerland: World Health Organization (WHO), 2015.
92. World Health Organization (WHO). WHO Mortality Database Version October 2017. Geneva, Switzerland: World Health Organization (WHO), 2017.

Japan

93. Ministry of Health, Labour and Welfare (Japan). Japan Vital Registration - Deaths 1980.
94. Ministry of Health, Labour and Welfare (Japan). Japan Vital Registration - Deaths 1981.
95. Ministry of Health, Labour and Welfare (Japan). Japan Vital Registration - Deaths 1982.
96. Ministry of Health, Labour and Welfare (Japan). Japan Vital Registration - Deaths 1983.
97. Ministry of Health, Labour and Welfare (Japan). Japan Vital Registration - Deaths 1984.
98. Ministry of Health, Labour and Welfare (Japan). Japan Vital Registration - Deaths 1985.
99. Ministry of Health, Labour and Welfare (Japan). Japan Vital Registration - Deaths 1986.
100. Ministry of Health, Labour and Welfare (Japan). Japan Vital Registration - Deaths 1987.
101. Ministry of Health, Labour and Welfare (Japan). Japan Vital Registration - Deaths 1988.
102. Ministry of Health, Labour and Welfare (Japan). Japan Vital Registration - Deaths 1989.
103. Ministry of Health, Labour and Welfare (Japan). Japan Vital Registration - Deaths 1990.
104. Ministry of Health, Labour and Welfare (Japan). Japan Vital Registration - Deaths 1991.
105. Ministry of Health, Labour and Welfare (Japan). Japan Vital Registration - Deaths 1992.
106. Ministry of Health, Labour and Welfare (Japan). Japan Vital Registration - Deaths 1993.
107. Ministry of Health, Labour and Welfare (Japan). Japan Vital Registration - Deaths 1994.
108. Ministry of Health, Labour and Welfare (Japan). Japan Vital Registration - Deaths 1995.
109. Ministry of Health, Labour and Welfare (Japan). Japan Vital Registration - Deaths 1996.
110. Ministry of Health, Labour and Welfare (Japan). Japan Vital Registration - Deaths 1997.
111. Ministry of Health, Labour and Welfare (Japan). Japan Vital Registration - Deaths 1998.
112. Ministry of Health, Labour and Welfare (Japan). Japan Vital Registration - Deaths 1999.
113. Ministry of Health, Labour and Welfare (Japan). Japan Vital Registration - Deaths 2000.
114. Ministry of Health, Labour and Welfare (Japan). Japan Vital Registration - Deaths 2001.
115. Ministry of Health, Labour and Welfare (Japan). Japan Vital Registration - Deaths 2002.
116. Ministry of Health, Labour and Welfare (Japan). Japan Vital Registration - Deaths 2003.
117. Ministry of Health, Labour and Welfare (Japan). Japan Vital Registration - Deaths 2004.
118. Ministry of Health, Labour and Welfare (Japan). Japan Vital Registration - Deaths 2005.
119. Ministry of Health, Labour and Welfare (Japan). Japan Vital Registration - Deaths 2006.

120. Ministry of Health, Labour and Welfare (Japan). Japan Vital Registration - Deaths 2007.
121. Ministry of Health, Labour and Welfare (Japan). Japan Vital Registration - Deaths 2008.
122. Ministry of Health, Labour and Welfare (Japan). Japan Vital Registration - Deaths 2009.
123. Ministry of Health, Labour and Welfare (Japan). Japan Vital Registration - Deaths 2010.
124. Ministry of Health, Labour and Welfare (Japan). Japan Vital Registration - Deaths 2011.
125. Ministry of Health, Labour and Welfare (Japan). Japan Vital Registration - Deaths 2012.
126. Ministry of Health, Labour and Welfare (Japan). Japan Vital Registration - Deaths 2013.
127. Ministry of Health, Labour and Welfare (Japan). Japan Vital Registration - Deaths 2014.
128. Ministry of Health, Labour and Welfare (Japan). Japan Vital Registration - Deaths 2015.

Kazakhstan

129. World Health Organization (WHO). WHO Mortality Database Version September 2016. Geneva, Switzerland: World Health Organization (WHO), 2016.
130. World Health Organization (WHO). WHO Mortality Database Version September 2016. Geneva, Switzerland: World Health Organization (WHO), 2016.

Kuwait

131. World Health Organization (WHO). WHO Mortality Database Version September 2016. Geneva, Switzerland: World Health Organization (WHO), 2016.
132. World Health Organization (WHO). WHO Mortality Database Version October 2017. Geneva, Switzerland: World Health Organization (WHO), 2017.

Latvia

133. World Health Organization (WHO). WHO Mortality Database Version October 2017. Geneva, Switzerland: World Health Organization (WHO), 2017.

Lithuania

134. World Health Organization (WHO). WHO Mortality Database Version November 2015. Geneva, Switzerland: World Health Organization (WHO), 2015.
135. World Health Organization (WHO). WHO Mortality Database Version September 2016. Geneva, Switzerland: World Health Organization (WHO), 2016.
136. World Health Organization (WHO). WHO Mortality Database Version March 2017. Geneva, Switzerland: World Health Organization (WHO), 2017.
137. World Health Organization (WHO). WHO Mortality Database Version October 2017. Geneva, Switzerland: World Health Organization (WHO), 2017.
138. World Health Organization (WHO). WHO Mortality Database Version April 2018. Geneva, Switzerland: World Health Organization (WHO), 2018.

Luxembourg

139. World Health Organization (WHO). WHO Mortality Database Version November 2015. Geneva, Switzerland: World Health Organization (WHO), 2015.
140. World Health Organization (WHO). WHO Mortality Database Version September 2016. Geneva, Switzerland: World Health Organization (WHO), 2016.

141. World Health Organization (WHO). WHO Mortality Database Version October 2017. Geneva, Switzerland: World Health Organization (WHO), 2017.

Malta

142. World Health Organization (WHO). WHO Mortality Database Version November 2015. Geneva, Switzerland: World Health Organization (WHO), 2015.
143. World Health Organization (WHO). WHO Mortality Database Version October 2017. Geneva, Switzerland: World Health Organization (WHO), 2017.
144. World Health Organization (WHO). WHO Mortality Database Version April 2018. Geneva, Switzerland: World Health Organization (WHO), 2018.

Mauritius

145. World Health Organization (WHO). WHO Mortality Database Version November 2015. Geneva, Switzerland: World Health Organization (WHO), 2015.
146. World Health Organization (WHO). WHO Mortality Database Version September 2016. Geneva, Switzerland: World Health Organization (WHO), 2016.
147. World Health Organization (WHO). WHO Mortality Database Version October 2017. Geneva, Switzerland: World Health Organization (WHO), 2017.

Mexico

148. Ministry of Health (Mexico), National Institute of Statistics and Geography (INEGI) (Mexico). Mexico Vital Registration - Deaths 1980.
149. Ministry of Health (Mexico), National Institute of Statistics and Geography (INEGI) (Mexico). Mexico Vital Registration - Deaths 1981.
150. Ministry of Health (Mexico), National Institute of Statistics and Geography (INEGI) (Mexico). Mexico Vital Registration - Deaths 1982.
151. Ministry of Health (Mexico), National Institute of Statistics and Geography (INEGI) (Mexico). Mexico Vital Registration - Deaths 1983.
152. Ministry of Health (Mexico), National Institute of Statistics and Geography (INEGI) (Mexico). Mexico Vital Registration - Deaths 1984.
153. Ministry of Health (Mexico), National Institute of Statistics and Geography (INEGI) (Mexico). Mexico Vital Registration - Deaths 1985.
154. Ministry of Health (Mexico), National Institute of Statistics and Geography (INEGI) (Mexico). Mexico Vital Registration - Deaths 1986.
155. Ministry of Health (Mexico), National Institute of Statistics and Geography (INEGI) (Mexico). Mexico Vital Registration - Deaths 1987.
156. Ministry of Health (Mexico), National Institute of Statistics and Geography (INEGI) (Mexico). Mexico Vital Registration - Deaths 1988.
157. Ministry of Health (Mexico), National Institute of Statistics and Geography (INEGI) (Mexico). Mexico Vital Registration - Deaths 1989.
158. Ministry of Health (Mexico), National Institute of Statistics and Geography (INEGI) (Mexico). Mexico Vital Registration - Deaths 1990.
159. Ministry of Health (Mexico), National Institute of Statistics and Geography (INEGI) (Mexico). Mexico Vital Registration - Deaths 1991.

160. Ministry of Health (Mexico), National Institute of Statistics and Geography (INEGI) (Mexico). Mexico Vital Registration - Deaths 1992.
161. Ministry of Health (Mexico), National Institute of Statistics and Geography (INEGI) (Mexico). Mexico Vital Registration - Deaths 1993.
162. Ministry of Health (Mexico), National Institute of Statistics and Geography (INEGI) (Mexico). Mexico Vital Registration - Deaths 1994.
163. Ministry of Health (Mexico), National Institute of Statistics and Geography (INEGI) (Mexico). Mexico Vital Registration - Deaths 1995.
164. Ministry of Health (Mexico), National Institute of Statistics and Geography (INEGI) (Mexico). Mexico Vital Registration - Deaths 1996.
165. Ministry of Health (Mexico), National Institute of Statistics and Geography (INEGI) (Mexico). Mexico Vital Registration - Deaths 1997.
166. Ministry of Health (Mexico), National Institute of Statistics and Geography (INEGI) (Mexico). Mexico Vital Registration - Deaths 1998.
167. Ministry of Health (Mexico), National Institute of Statistics and Geography (INEGI) (Mexico). Mexico Vital Registration - Deaths 1999.
168. Ministry of Health (Mexico), National Institute of Statistics and Geography (INEGI) (Mexico). Mexico Vital Registration - Deaths 2000.
169. Ministry of Health (Mexico), National Institute of Statistics and Geography (INEGI) (Mexico). Mexico Vital Registration - Deaths 2001.
170. Ministry of Health (Mexico), National Institute of Statistics and Geography (INEGI) (Mexico). Mexico Vital Registration - Deaths 2002.
171. Ministry of Health (Mexico), National Institute of Statistics and Geography (INEGI) (Mexico). Mexico Vital Registration - Deaths 2003.
172. Ministry of Health (Mexico), National Institute of Statistics and Geography (INEGI) (Mexico). Mexico Vital Registration - Deaths 2004.
173. Ministry of Health (Mexico), National Institute of Statistics and Geography (INEGI) (Mexico). Mexico Vital Registration - Deaths 2005.
174. Ministry of Health (Mexico), National Institute of Statistics and Geography (INEGI) (Mexico). Mexico Vital Registration - Deaths 2006.
175. Ministry of Health (Mexico), National Institute of Statistics and Geography (INEGI) (Mexico). Mexico Vital Registration - Deaths 2007.
176. Ministry of Health (Mexico), National Institute of Statistics and Geography (INEGI) (Mexico). Mexico Vital Registration - Deaths 2008.
177. Ministry of Health (Mexico), National Institute of Statistics and Geography (INEGI) (Mexico). Mexico Vital Registration - Deaths 2010.
178. Ministry of Health (Mexico), National Institute of Statistics and Geography (INEGI) (Mexico). Mexico Vital Registration - Deaths 2011.
179. National Institute of Statistics and Geography (INEGI) (Mexico). Mexico Vital Registration - Deaths 2012.
180. National Institute of Statistics and Geography (INEGI) (Mexico). Mexico Vital Registration - Deaths 2013.
181. National Institute of Statistics and Geography (INEGI) (Mexico). Mexico Vital Registration - Deaths 2014. Mexico City, Mexico: National Institute of Statistics and Geography (INEGI) (Mexico).

182. National Institute of Statistics and Geography (INEGI) (Mexico). Mexico Vital Registration - Deaths 2015. Mexico City, Mexico: National Institute of Statistics and Geography (INEGI) (Mexico).
183. National Institute of Statistics and Geography (INEGI) (Mexico). Mexico Vital Registration - Deaths 2016. Mexico City, Mexico: National Institute of Statistics and Geography (INEGI) (Mexico).
184. National Institute of Statistics and Geography (INEGI) (Mexico). Mexico Vital Statistics - Deaths 2009.

Moldova

185. World Health Organization (WHO). WHO Mortality Database Version September 2016. Geneva, Switzerland: World Health Organization (WHO), 2016.
186. World Health Organization (WHO). WHO Mortality Database Version March 2017. Geneva, Switzerland: World Health Organization (WHO), 2017.
187. World Health Organization (WHO). WHO Mortality Database Version October 2017. Geneva, Switzerland: World Health Organization (WHO), 2017.
188. World Health Organization (WHO). WHO Mortality Database Version April 2018. Geneva, Switzerland: World Health Organization (WHO), 2018.

Netherlands

189. World Health Organization (WHO). WHO Mortality Database Version November 2015. Geneva, Switzerland: World Health Organization (WHO), 2015.
190. World Health Organization (WHO). WHO Mortality Database Version March 2017. Geneva, Switzerland: World Health Organization (WHO), 2017.
191. World Health Organization (WHO). WHO Mortality Database Version October 2017. Geneva, Switzerland: World Health Organization (WHO), 2017.
192. World Health Organization (WHO). WHO Mortality Database Version April 2018. Geneva, Switzerland: World Health Organization (WHO), 2018.

New Zealand

193. Ministry of Health (New Zealand). New Zealand Mortality Data 1980. Wellington, New Zealand: Ministry of Health (New Zealand).
194. Ministry of Health (New Zealand). New Zealand Mortality Data 1981. Wellington, New Zealand: Ministry of Health (New Zealand).
195. Ministry of Health (New Zealand). New Zealand Mortality Data 1982. Wellington, New Zealand: Ministry of Health (New Zealand).
196. Ministry of Health (New Zealand). New Zealand Mortality Data 1983. Wellington, New Zealand: Ministry of Health (New Zealand).
197. Ministry of Health (New Zealand). New Zealand Mortality Data 1984. Wellington, New Zealand: Ministry of Health (New Zealand).
198. Ministry of Health (New Zealand). New Zealand Mortality Data 1985. Wellington, New Zealand: Ministry of Health (New Zealand).
199. Ministry of Health (New Zealand). New Zealand Mortality Data 1986. Wellington, New Zealand: Ministry of Health (New Zealand).
200. Ministry of Health (New Zealand). New Zealand Mortality Data 1987. Wellington, New Zealand: Ministry of Health (New Zealand).

201. Ministry of Health (New Zealand). New Zealand Mortality Collection 1988. Wellington, New Zealand: Ministry of Health (New Zealand).
202. Ministry of Health (New Zealand). New Zealand Mortality Collection 1989. Wellington, New Zealand: Ministry of Health (New Zealand).
203. Ministry of Health (New Zealand). New Zealand Mortality Collection 1990. Wellington, New Zealand: Ministry of Health (New Zealand).
204. Ministry of Health (New Zealand). New Zealand Mortality Collection 1991. Wellington, New Zealand: Ministry of Health (New Zealand).
205. Ministry of Health (New Zealand). New Zealand Mortality Collection 1992. Wellington, New Zealand: Ministry of Health (New Zealand).
206. Ministry of Health (New Zealand). New Zealand Mortality Collection 1993. Wellington, New Zealand: Ministry of Health (New Zealand).
207. Ministry of Health (New Zealand). New Zealand Mortality Collection 1994. Wellington, New Zealand: Ministry of Health (New Zealand).
208. Ministry of Health (New Zealand). New Zealand Mortality Collection 1995. Wellington, New Zealand: Ministry of Health (New Zealand).
209. Ministry of Health (New Zealand). New Zealand Mortality Collection 1996. Wellington, New Zealand: Ministry of Health (New Zealand).
210. Ministry of Health (New Zealand). New Zealand Mortality Collection 1997. Wellington, New Zealand: Ministry of Health (New Zealand).
211. Ministry of Health (New Zealand). New Zealand Mortality Collection 1998. Wellington, New Zealand: Ministry of Health (New Zealand).
212. Ministry of Health (New Zealand). New Zealand Mortality Collection 1999. Wellington, New Zealand: Ministry of Health (New Zealand).
213. Ministry of Health (New Zealand). New Zealand Mortality Collection 2000. Wellington, New Zealand: Ministry of Health (New Zealand).
214. Ministry of Health (New Zealand). New Zealand Mortality Collection 2001. Wellington, New Zealand: Ministry of Health (New Zealand).
215. Ministry of Health (New Zealand). New Zealand Mortality Collection 2002. Wellington, New Zealand: Ministry of Health (New Zealand).
216. Ministry of Health (New Zealand). New Zealand Mortality Collection 2003. Wellington, New Zealand: Ministry of Health (New Zealand).
217. Ministry of Health (New Zealand). New Zealand Mortality Collection 2004. Wellington, New Zealand: Ministry of Health (New Zealand).
218. Ministry of Health (New Zealand). New Zealand Mortality Collection 2005. Wellington, New Zealand: Ministry of Health (New Zealand).
219. Ministry of Health (New Zealand). New Zealand Mortality Collection 2006. Wellington, New Zealand: Ministry of Health (New Zealand).
220. Ministry of Health (New Zealand). New Zealand Mortality Collection 2007. Wellington, New Zealand: Ministry of Health (New Zealand).
221. Ministry of Health (New Zealand). New Zealand Mortality Collection 2008. Wellington, New Zealand: Ministry of Health (New Zealand).
222. Ministry of Health (New Zealand). New Zealand Mortality Collection 2009. Wellington, New Zealand: Ministry of Health (New Zealand).

223. Ministry of Health (New Zealand). New Zealand Mortality Collection 2010. Wellington, New Zealand: Ministry of Health (New Zealand).
224. Ministry of Health (New Zealand). New Zealand Mortality Collection 2011. Wellington, New Zealand: Ministry of Health (New Zealand).
225. Ministry of Health (New Zealand). New Zealand Mortality Collection 2012. Wellington, New Zealand: Ministry of Health (New Zealand).
226. Ministry of Health (New Zealand). New Zealand Mortality Collection 2013. Wellington, New Zealand: Ministry of Health (New Zealand).
227. Ministry of Health (New Zealand). New Zealand Mortality Collection 2014. Wellington, New Zealand: Ministry of Health (New Zealand).
228. Ministry of Health (New Zealand). New Zealand Mortality Collection 2015. Wellington, New Zealand: Ministry of Health (New Zealand).

Norway

229. Norwegian Institute of Public Health. Norway Cause of Death Registry 1980. Oslo, Norway: Norwegian Institute of Public Health.
230. Norwegian Institute of Public Health. Norway Cause of Death Registry 1981. Oslo, Norway: Norwegian Institute of Public Health.
231. Norwegian Institute of Public Health. Norway Cause of Death Registry 1982. Oslo, Norway: Norwegian Institute of Public Health.
232. Norwegian Institute of Public Health. Norway Cause of Death Registry 1983. Oslo, Norway: Norwegian Institute of Public Health.
233. Norwegian Institute of Public Health. Norway Cause of Death Registry 1984. Oslo, Norway: Norwegian Institute of Public Health.
234. Norwegian Institute of Public Health. Norway Cause of Death Registry 1985. Oslo, Norway: Norwegian Institute of Public Health.
235. Norwegian Institute of Public Health. Norway Cause of Death Registry 1986. Oslo, Norway: Norwegian Institute of Public Health.
236. Norwegian Institute of Public Health. Norway Cause of Death Registry 1987. Oslo, Norway: Norwegian Institute of Public Health.
237. Norwegian Institute of Public Health. Norway Cause of Death Registry 1988. Oslo, Norway: Norwegian Institute of Public Health.
238. Norwegian Institute of Public Health. Norway Cause of Death Registry 1989. Oslo, Norway: Norwegian Institute of Public Health.
239. Norwegian Institute of Public Health. Norway Cause of Death Registry 1990. Oslo, Norway: Norwegian Institute of Public Health.
240. Norwegian Institute of Public Health. Norway Cause of Death Registry 1991. Oslo, Norway: Norwegian Institute of Public Health.
241. Norwegian Institute of Public Health. Norway Cause of Death Registry 1992. Oslo, Norway: Norwegian Institute of Public Health.
242. Norwegian Institute of Public Health. Norway Cause of Death Registry 1993. Oslo, Norway: Norwegian Institute of Public Health.
243. Norwegian Institute of Public Health. Norway Cause of Death Registry 1994. Oslo, Norway: Norwegian Institute of Public Health.

244. Norwegian Institute of Public Health. Norway Cause of Death Registry 1995. Oslo, Norway: Norwegian Institute of Public Health.
245. Norwegian Institute of Public Health. Norway Cause of Death Registry 1996. Oslo, Norway: Norwegian Institute of Public Health.
246. Norwegian Institute of Public Health. Norway Cause of Death Registry 1997. Oslo, Norway: Norwegian Institute of Public Health.
247. Norwegian Institute of Public Health. Norway Cause of Death Registry 1998. Oslo, Norway: Norwegian Institute of Public Health.
248. Norwegian Institute of Public Health. Norway Cause of Death Registry 1999. Oslo, Norway: Norwegian Institute of Public Health.
249. Norwegian Institute of Public Health. Norway Cause of Death Registry 2000. Oslo, Norway: Norwegian Institute of Public Health.
250. Norwegian Institute of Public Health. Norway Cause of Death Registry 2001. Oslo, Norway: Norwegian Institute of Public Health.
251. Norwegian Institute of Public Health. Norway Cause of Death Registry 2002. Oslo, Norway: Norwegian Institute of Public Health.
252. Norwegian Institute of Public Health. Norway Cause of Death Registry 2003. Oslo, Norway: Norwegian Institute of Public Health.
253. Norwegian Institute of Public Health. Norway Cause of Death Registry 2004. Oslo, Norway: Norwegian Institute of Public Health.
254. Norwegian Institute of Public Health. Norway Cause of Death Registry 2005. Oslo, Norway: Norwegian Institute of Public Health.
255. Norwegian Institute of Public Health. Norway Cause of Death Registry 2006. Oslo, Norway: Norwegian Institute of Public Health.
256. Norwegian Institute of Public Health. Norway Cause of Death Registry 2007. Oslo, Norway: Norwegian Institute of Public Health.
257. Norwegian Institute of Public Health. Norway Cause of Death Registry 2008. Oslo, Norway: Norwegian Institute of Public Health.
258. Norwegian Institute of Public Health. Norway Cause of Death Registry 2009. Oslo, Norway: Norwegian Institute of Public Health.
259. Norwegian Institute of Public Health. Norway Cause of Death Registry 2010. Oslo, Norway: Norwegian Institute of Public Health.
260. Norwegian Institute of Public Health. Norway Cause of Death Registry 2011. Oslo, Norway: Norwegian Institute of Public Health.
261. Norwegian Institute of Public Health. Norway Cause of Death Registry 2012. Oslo, Norway: Norwegian Institute of Public Health.
262. Norwegian Institute of Public Health. Norway Cause of Death Registry 2013. Oslo, Norway: Norwegian Institute of Public Health.
263. Norwegian Institute of Public Health. Norway Cause of Death Registry 2014. Oslo, Norway: Norwegian Institute of Public Health.
264. Norwegian Institute of Public Health. Norway Cause of Death Registry 2015. Oslo, Norway: Norwegian Institute of Public Health.

Philippines

265. National Statistics Office (Philippines), Philippines Statistics Authority. Philippines Vital Registration - Deaths 2012.
266. Philippines Statistics Authority. Philippines Vital Registration - Deaths 2013.
267. Philippines Statistics Authority. Philippines Vital Registration - Deaths 2014.
268. Philippines Statistics Authority. Philippines Vital Registration - Deaths 2015.
269. World Health Organization (WHO). WHO Mortality Database Version November 2015. Geneva, Switzerland: World Health Organization (WHO), 2015.
270. World Health Organization (WHO). WHO Mortality Database Version September 2016. Geneva, Switzerland: World Health Organization (WHO), 2016.
271. World Health Organization (WHO). WHO Mortality Database Version October 2017. Geneva, Switzerland: World Health Organization (WHO), 2017.

Poland

272. World Health Organization (WHO). WHO Mortality Database Version September 2016. Geneva, Switzerland: World Health Organization (WHO), 2016.
273. World Health Organization (WHO). WHO Mortality Database Version October 2017. Geneva, Switzerland: World Health Organization (WHO), 2017.

Portugal

274. Statistics Portugal. Portugal Vital Registration - Deaths 1980.
275. Statistics Portugal. Portugal Vital Registration - Deaths 1981.
276. Statistics Portugal. Portugal Vital Registration - Deaths 1982.
277. Statistics Portugal. Portugal Vital Registration - Deaths 1983.
278. Statistics Portugal. Portugal Vital Registration - Deaths 2004.
279. World Health Organization (WHO). WHO Mortality Database Version November 2015. Geneva, Switzerland: World Health Organization (WHO), 2015.
280. World Health Organization (WHO). WHO Mortality Database Version October 2017. Geneva, Switzerland: World Health Organization (WHO), 2017.

Puerto Rico

281. National Center for Health Statistics (NCHS), Centers for Disease Control and Prevention (CDC). United States NVSS Custom Mortality Data 2005. Atlanta, United States: Centers for Disease Control and Prevention (CDC).
282. National Center for Health Statistics (NCHS), Centers for Disease Control and Prevention (CDC). United States NVSS Custom Mortality Data 2006. Atlanta, United States: Centers for Disease Control and Prevention (CDC).
283. National Center for Health Statistics (NCHS), Centers for Disease Control and Prevention (CDC). United States NVSS Custom Mortality Data 2007. Atlanta, United States: Centers for Disease Control and Prevention (CDC).
284. National Center for Health Statistics (NCHS), Centers for Disease Control and Prevention (CDC). United States NVSS Custom Mortality Data 2008. Atlanta, United States: Centers for Disease Control and Prevention (CDC).

285. National Center for Health Statistics (NCHS), Centers for Disease Control and Prevention (CDC). United States NVSS Custom Mortality Data 2009. Atlanta, United States: Centers for Disease Control and Prevention (CDC).
286. National Center for Health Statistics (NCHS), Centers for Disease Control and Prevention (CDC). United States NVSS Custom Mortality Data 2010. Atlanta, United States: Centers for Disease Control and Prevention (CDC).
287. National Center for Health Statistics (NCHS), Centers for Disease Control and Prevention (CDC). United States NVSS Custom Mortality Data 2011. Atlanta, United States: Centers for Disease Control and Prevention (CDC).
288. National Center for Health Statistics (NCHS), Centers for Disease Control and Prevention (CDC). United States NVSS Custom Mortality Data 2012. Atlanta, United States: Centers for Disease Control and Prevention (CDC).
289. National Center for Health Statistics (NCHS), Centers for Disease Control and Prevention (CDC). United States NVSS Custom Mortality Data 2013. Atlanta, United States: Centers for Disease Control and Prevention (CDC).
290. National Center for Health Statistics (NCHS), Centers for Disease Control and Prevention (CDC). United States NVSS Custom Mortality Data 2014. Atlanta, United States: Centers for Disease Control and Prevention (CDC).
291. National Center for Health Statistics (NCHS), Centers for Disease Control and Prevention (CDC). United States NVSS Custom Mortality Data 2015. Atlanta, United States: Centers for Disease Control and Prevention (CDC).
292. National Center for Health Statistics (NCHS), Centers for Disease Control and Prevention (CDC). United States NVSS Mortality Data 1994 - NBER. Atlanta, United States: Centers for Disease Control and Prevention (CDC).
293. National Center for Health Statistics (NCHS), Centers for Disease Control and Prevention (CDC). United States NVSS Mortality Data 1995 - NBER. Atlanta, United States: Centers for Disease Control and Prevention (CDC).
294. National Center for Health Statistics (NCHS), Centers for Disease Control and Prevention (CDC). United States NVSS Mortality Data 1996 - NBER. Atlanta, United States: Centers for Disease Control and Prevention (CDC).
295. National Center for Health Statistics (NCHS), Centers for Disease Control and Prevention (CDC). United States NVSS Mortality Data 1997 - NBER. Atlanta, United States: Centers for Disease Control and Prevention (CDC).
296. National Center for Health Statistics (NCHS), Centers for Disease Control and Prevention (CDC). United States NVSS Mortality Data 1998 - NBER. Atlanta, United States: Centers for Disease Control and Prevention (CDC).
297. National Center for Health Statistics (NCHS), Centers for Disease Control and Prevention (CDC). United States NVSS Mortality Data 1999 - NBER. Atlanta, United States: Centers for Disease Control and Prevention (CDC).
298. National Center for Health Statistics (NCHS), Centers for Disease Control and Prevention (CDC). United States NVSS Mortality Data 2000 - NBER. Atlanta, United States: Centers for Disease Control and Prevention (CDC).

299. National Center for Health Statistics (NCHS), Centers for Disease Control and Prevention (CDC). United States NVSS Mortality Data 2001 - NBER. Atlanta, United States: Centers for Disease Control and Prevention (CDC).
300. National Center for Health Statistics (NCHS), Centers for Disease Control and Prevention (CDC). United States NVSS Mortality Data 2002 - NBER. Atlanta, United States: Centers for Disease Control and Prevention (CDC).
301. National Center for Health Statistics (NCHS), Centers for Disease Control and Prevention (CDC). United States NVSS Mortality Data 2003 - NBER. Atlanta, United States: Centers for Disease Control and Prevention (CDC).
302. National Center for Health Statistics (NCHS), Centers for Disease Control and Prevention (CDC). United States NVSS Mortality Data 2004 - NBER. Atlanta, United States: Centers for Disease Control and Prevention (CDC).
303. World Health Organization (WHO). WHO Mortality Database Version September 2016. Geneva, Switzerland: World Health Organization (WHO), 2016.

Romania

304. World Health Organization (WHO). WHO Mortality Database Version September 2016. Geneva, Switzerland: World Health Organization (WHO), 2016.
305. World Health Organization (WHO). WHO Mortality Database Version March 2017. Geneva, Switzerland: World Health Organization (WHO), 2017.
306. World Health Organization (WHO). WHO Mortality Database Version October 2017. Geneva, Switzerland: World Health Organization (WHO), 2017.
307. World Health Organization (WHO). WHO Mortality Database Version April 2018. Geneva, Switzerland: World Health Organization (WHO), 2018.

Russian Federation

308. Center for Demographic Research, New Economic School (Russia). Russia Mortality Rates by Region, Age, Sex, and Cause of Death 1999-2005. Moscow, Russia: Center for Demographic Research, New Economic School (Russia).
309. Center for Demographic Research, New Economic School (Russia). Russia Mortality Rates by Region, Age, Sex, and Cause of Death 2006-2012. Moscow, Russia: Center for Demographic Research, New Economic School (Russia). http://demogr.nes.ru/index.php/ru/demogr_indicat/data
310. Center for Demographic Research, New Economic School (Russia). Russia Mortality Rates by Region, Cause of Death, 5-Year Age Groups, and Sex, 2011-2014. Moscow, Russia: Center for Demographic Research, New Economic School (Russia). http://demogr.nes.ru/index.php/ru/demogr_indicat/data
311. Center for Demographic Research, New Economic School (Russia). Russia Mortality Rates by Region, 1-Year Age Groups, and Sex, 2015-2016. Moscow, Russia: Center for Demographic Research, New Economic School (Russia). http://demogr.nes.ru/index.php/ru/demogr_indicat/data

Saint Lucia

312. World Health Organization (WHO). WHO Mortality Database Version September 2016. Geneva, Switzerland: World Health Organization (WHO), 2016.
313. World Health Organization (WHO). WHO Mortality Database Version October 2017. Geneva, Switzerland: World Health Organization (WHO), 2017.

Saint Vincent and the Grenadines

314. World Health Organization (WHO). WHO Mortality Database Version November 2015. Geneva, Switzerland: World Health Organization (WHO), 2015.
315. World Health Organization (WHO). WHO Mortality Database Version September 2016. Geneva, Switzerland: World Health Organization (WHO), 2016.
316. World Health Organization (WHO). WHO Mortality Database Version October 2017. Geneva, Switzerland: World Health Organization (WHO), 2017.

Singapore

317. Ministry of Health (Singapore). Singapore Causes of Death 1980.
318. Ministry of Health (Singapore). Singapore Causes of Death 1981.
319. Ministry of Health (Singapore). Singapore Causes of Death 1982.
320. Ministry of Health (Singapore). Singapore Causes of Death 1983.
321. Ministry of Health (Singapore). Singapore Causes of Death 1984.
322. Ministry of Health (Singapore). Singapore Causes of Death 1985.
323. Ministry of Health (Singapore). Singapore Causes of Death 1986.
324. Ministry of Health (Singapore). Singapore Causes of Death 1987.
325. Ministry of Health (Singapore). Singapore Causes of Death 1988.
326. Ministry of Health (Singapore). Singapore Causes of Death 1989.
327. Ministry of Health (Singapore). Singapore Causes of Death 1990.
328. Ministry of Health (Singapore). Singapore Causes of Death 1991.
329. Ministry of Health (Singapore). Singapore Causes of Death 1992.
330. Ministry of Health (Singapore). Singapore Causes of Death 1993.
331. Ministry of Health (Singapore). Singapore Causes of Death 1994.
332. Ministry of Health (Singapore). Singapore Causes of Death 1995.
333. Ministry of Health (Singapore). Singapore Causes of Death 1996.
334. Ministry of Health (Singapore). Singapore Causes of Death 1997.
335. Ministry of Health (Singapore). Singapore Causes of Death 1998.
336. Ministry of Health (Singapore). Singapore Causes of Death 1999.
337. Ministry of Health (Singapore). Singapore Causes of Death 2000.
338. Ministry of Health (Singapore). Singapore Causes of Death 2001.
339. Ministry of Health (Singapore). Singapore Causes of Death 2002.
340. Ministry of Health (Singapore). Singapore Causes of Death 2003.
341. Ministry of Health (Singapore). Singapore Causes of Death 2004.
342. Ministry of Health (Singapore). Singapore Causes of Death 2005.
343. Ministry of Health (Singapore). Singapore Causes of Death 2006.
344. Ministry of Health (Singapore). Singapore Causes of Death 2007.
345. Ministry of Health (Singapore). Singapore Causes of Death 2008.
346. Ministry of Health (Singapore). Singapore Causes of Death 2009.

347. Ministry of Health (Singapore). Singapore Causes of Death 2010.
348. Ministry of Health (Singapore). Singapore Causes of Death 2011.
349. Ministry of Health (Singapore). Singapore Causes of Death 2012.
350. Ministry of Health (Singapore). Singapore Causes of Death 2013.
351. Ministry of Health (Singapore). Singapore Causes of Death 2014.
352. Ministry of Health (Singapore). Singapore Causes of Death 2015.
353. Ministry of Health (Singapore). Singapore Causes of Death 2016.

Slovenia

354. World Health Organization (WHO). WHO Mortality Database Version November 2015. Geneva, Switzerland: World Health Organization (WHO), 2015.
355. World Health Organization (WHO). WHO Mortality Database Version March 2017. Geneva, Switzerland: World Health Organization (WHO), 2017.
356. World Health Organization (WHO). WHO Mortality Database Version October 2017. Geneva, Switzerland: World Health Organization (WHO), 2017.

South Korea

357. World Health Organization (WHO). WHO Mortality Database Version September 2016. Geneva, Switzerland: World Health Organization (WHO), 2016.
358. World Health Organization (WHO). WHO Mortality Database Version October 2017. Geneva, Switzerland: World Health Organization (WHO), 2017.

Spain

359. World Health Organization (WHO). WHO Mortality Database Version November 2015. Geneva, Switzerland: World Health Organization (WHO), 2015.
360. World Health Organization (WHO). WHO Mortality Database Version October 2017. Geneva, Switzerland: World Health Organization (WHO), 2017.

Sweden

361. National Board of Health and Welfare (Sweden). Sweden Cause of Death Register 1980.
362. National Board of Health and Welfare (Sweden). Sweden Cause of Death Register 1981.
363. National Board of Health and Welfare (Sweden). Sweden Cause of Death Register 1982.
364. National Board of Health and Welfare (Sweden). Sweden Cause of Death Register 1983.
365. National Board of Health and Welfare (Sweden). Sweden Cause of Death Register 1984.
366. National Board of Health and Welfare (Sweden). Sweden Cause of Death Register 1985.
367. National Board of Health and Welfare (Sweden). Sweden Cause of Death Register 1986.
368. National Board of Health and Welfare (Sweden). Sweden Cause of Death Register 1987.
369. National Board of Health and Welfare (Sweden). Sweden Cause of Death Register 1988.
370. National Board of Health and Welfare (Sweden). Sweden Cause of Death Register 1989.
371. National Board of Health and Welfare (Sweden). Sweden Cause of Death Register 1990.
372. National Board of Health and Welfare (Sweden). Sweden Cause of Death Register 1991.
373. National Board of Health and Welfare (Sweden). Sweden Cause of Death Register 1992.
374. National Board of Health and Welfare (Sweden). Sweden Cause of Death Register 1993.
375. National Board of Health and Welfare (Sweden). Sweden Cause of Death Register 1994.

376. National Board of Health and Welfare (Sweden). Sweden Cause of Death Register 1995.
377. National Board of Health and Welfare (Sweden). Sweden Cause of Death Register 1996.
378. National Board of Health and Welfare (Sweden). Sweden Cause of Death Register 1997.
379. National Board of Health and Welfare (Sweden). Sweden Cause of Death Register 1998.
380. National Board of Health and Welfare (Sweden). Sweden Cause of Death Register 1999.
381. National Board of Health and Welfare (Sweden). Sweden Cause of Death Register 2000.
382. National Board of Health and Welfare (Sweden). Sweden Cause of Death Register 2001.
383. National Board of Health and Welfare (Sweden). Sweden Cause of Death Register 2002.
384. National Board of Health and Welfare (Sweden). Sweden Cause of Death Register 2003.
385. National Board of Health and Welfare (Sweden). Sweden Cause of Death Register 2004.
386. National Board of Health and Welfare (Sweden). Sweden Cause of Death Register 2005.
387. National Board of Health and Welfare (Sweden). Sweden Cause of Death Register 2006.
388. National Board of Health and Welfare (Sweden). Sweden Cause of Death Register 2007.
389. National Board of Health and Welfare (Sweden). Sweden Cause of Death Register 2008.
390. National Board of Health and Welfare (Sweden). Sweden Cause of Death Register 2009.
391. National Board of Health and Welfare (Sweden). Sweden Cause of Death Register 2010.
392. National Board of Health and Welfare (Sweden). Sweden Cause of Death Register 2011.
393. National Board of Health and Welfare (Sweden). Sweden Cause of Death Register 2012. Stockholm, Sweden: National Board of Health and Welfare (Sweden).
394. National Board of Health and Welfare (Sweden). Sweden Cause of Death Register 2013. Stockholm, Sweden: National Board of Health and Welfare (Sweden).
395. National Board of Health and Welfare (Sweden). Sweden Cause of Death Register 2014. Stockholm, Sweden: National Board of Health and Welfare (Sweden).
396. National Board of Health and Welfare (Sweden). Sweden Cause of Death Register 2015. Stockholm, Sweden: National Board of Health and Welfare (Sweden).
397. National Board of Health and Welfare (Sweden). Sweden Cause of Death Register 2016. Stockholm, Sweden: National Board of Health and Welfare (Sweden).
398. National Board of Health and Welfare (Sweden). Sweden Vital Registration - Deaths 1980.
399. National Board of Health and Welfare (Sweden). Sweden Vital Registration - Deaths 1981.
400. National Board of Health and Welfare (Sweden). Sweden Vital Registration - Deaths 1982.
401. National Board of Health and Welfare (Sweden). Sweden Vital Registration - Deaths 1983.
402. National Board of Health and Welfare (Sweden). Sweden Vital Registration - Deaths 1984.
403. National Board of Health and Welfare (Sweden). Sweden Vital Registration - Deaths 1985.
404. National Board of Health and Welfare (Sweden). Sweden Vital Registration - Deaths 1986.

Switzerland

405. World Health Organization (WHO). WHO Mortality Database Version October 2017. Geneva, Switzerland: World Health Organization (WHO), 2017.
406. World Health Organization (WHO). WHO Mortality Database Version April 2018. Geneva, Switzerland: World Health Organization (WHO), 2018.

Taiwan

407. Department of Health (Taiwan). Taiwan Vital Registration - Deaths 1980.
408. Department of Health (Taiwan). Taiwan Vital Registration - Deaths 1981.

409. Department of Health (Taiwan). Taiwan Vital Registration - Deaths 1982.
410. Department of Health (Taiwan). Taiwan Vital Registration - Deaths 1983.
411. Department of Health (Taiwan). Taiwan Vital Registration - Deaths 1984.
412. Department of Health (Taiwan). Taiwan Vital Registration - Deaths 1985.
413. Department of Health (Taiwan). Taiwan Vital Registration - Deaths 1986.
414. Department of Health (Taiwan). Taiwan Vital Registration - Deaths 1987.
415. Department of Health (Taiwan). Taiwan Vital Registration - Deaths 1988.
416. Department of Health (Taiwan). Taiwan Vital Registration - Deaths 1989.
417. Department of Health (Taiwan). Taiwan Vital Registration - Deaths 1990.
418. Department of Health (Taiwan). Taiwan Vital Registration - Deaths 1991.
419. Department of Health (Taiwan). Taiwan Vital Registration - Deaths 1992. Taipei City, Taiwan: Ministry of Health and Welfare (Taiwan).
420. Department of Health (Taiwan). Taiwan Vital Registration - Deaths 1993. Taipei City, Taiwan: Ministry of Health and Welfare (Taiwan).
421. Department of Health (Taiwan). Taiwan Vital Registration - Deaths 1994. Taipei City, Taiwan: Ministry of Health and Welfare (Taiwan).
422. Department of Health (Taiwan). Taiwan Vital Registration - Deaths 1995. Taipei City, Taiwan: Ministry of Health and Welfare (Taiwan).
423. Department of Health (Taiwan). Taiwan Vital Registration - Deaths 1996. Taipei City, Taiwan: Ministry of Health and Welfare (Taiwan).
424. Department of Health (Taiwan). Taiwan Vital Registration - Deaths 1997. Taipei City, Taiwan: Ministry of Health and Welfare (Taiwan).
425. Department of Health (Taiwan). Taiwan Vital Registration - Deaths 1998. Taipei City, Taiwan: Ministry of Health and Welfare (Taiwan).
426. Department of Health (Taiwan). Taiwan Vital Registration - Deaths 1999. Taipei City, Taiwan: Ministry of Health and Welfare (Taiwan).
427. Department of Health (Taiwan). Taiwan Vital Registration - Deaths 2000. Taipei City, Taiwan: Ministry of Health and Welfare (Taiwan).
428. Department of Health (Taiwan). Taiwan Vital Registration - Deaths 2001. Taipei City, Taiwan: Ministry of Health and Welfare (Taiwan).
429. Department of Health (Taiwan). Taiwan Vital Registration - Deaths 2002. Taipei City, Taiwan: Ministry of Health and Welfare (Taiwan).
430. Department of Health (Taiwan). Taiwan Vital Registration - Deaths 2003. Taipei City, Taiwan: Ministry of Health and Welfare (Taiwan).
431. Department of Health (Taiwan). Taiwan Vital Registration - Deaths 2004. Taipei City, Taiwan: Ministry of Health and Welfare (Taiwan).
432. Department of Health (Taiwan). Taiwan Vital Registration - Deaths 2005. Taipei City, Taiwan: Ministry of Health and Welfare (Taiwan).
433. Department of Health (Taiwan). Taiwan Vital Registration - Deaths 2006. Taipei City, Taiwan: Ministry of Health and Welfare (Taiwan).
434. Department of Health (Taiwan). Taiwan Vital Registration - Deaths 2007. Taipei City, Taiwan: Ministry of Health and Welfare (Taiwan).
435. Department of Health (Taiwan). Taiwan Vital Registration - Deaths 2008. Taipei City, Taiwan: Ministry of Health and Welfare (Taiwan).

436. Department of Health (Taiwan). Taiwan Vital Registration - Deaths 2009. Taipei City, Taiwan: Ministry of Health and Welfare (Taiwan).
437. Department of Health (Taiwan). Taiwan Vital Registration - Deaths 2010. Taipei City, Taiwan: Ministry of Health and Welfare (Taiwan).
438. Department of Health (Taiwan). Taiwan Vital Registration - Deaths 2011. Taipei City, Taiwan: Ministry of Health and Welfare (Taiwan).
439. Department of Health (Taiwan). Taiwan Vital Registration - Deaths 2012. Taipei City, Taiwan: Ministry of Health and Welfare (Taiwan).
440. Ministry of Health and Welfare (Taiwan). Taiwan Vital Registration - Deaths 2013. Taipei City, Taiwan: Ministry of Health and Welfare (Taiwan).
441. Ministry of Health and Welfare (Taiwan). Taiwan Vital Registration - Deaths 2014. Taipei City, Taiwan: Ministry of Health and Welfare (Taiwan).
442. Ministry of Health and Welfare (Taiwan). Taiwan Vital Registration - Deaths 2015. Taipei City, Taiwan: Ministry of Health and Welfare (Taiwan).
443. Ministry of Health and Welfare (Taiwan). Taiwan Vital Registration - Deaths 2016. Taipei City, Taiwan: Ministry of Health and Welfare (Taiwan).

Trinidad and Tobago

444. World Health Organization (WHO). WHO Mortality Database Version September 2016. Geneva, Switzerland: World Health Organization (WHO), 2016.
445. World Health Organization (WHO). WHO Mortality Database Version October 2017. Geneva, Switzerland: World Health Organization (WHO), 2017.

Ukraine

446. Center for Demographic Research, New Economic School (Russia). Russia Mortality Rates by Region, 1-Year Age Groups, and Sex, 2015-2016. Moscow, Russia: Center for Demographic Research, New Economic School (Russia). http://demogr.nes.ru/index.php/ru/demogr_indicat/data
447. State Statistics Service (Ukraine). Ukraine Vital Registration - Deaths 2013.
448. State Statistics Service (Ukraine). Ukraine Vital Registration - Deaths 2015.
449. State Statistics Service (Ukraine). Ukraine Vital Registration - Deaths 2016.

United Kingdom

450. National Records of Scotland. United Kingdom - Scotland Vital Events Reference Tables 2015. Edinburgh, Scotland: National Records of Scotland, 2016.
451. National Records of Scotland. United Kingdom - Scotland Vital Events Reference Tables 2016. Edinburgh, Scotland: National Records of Scotland, 2017.
452. Office for National Statistics (United Kingdom). United Kingdom - England and Wales Mortality Data 1981-1994.
453. Office for National Statistics (United Kingdom). United Kingdom - England and Wales Mortality Data 1995-2000.
454. Office for National Statistics (United Kingdom). United Kingdom - England and Wales Mortality Data 2001-2012.

455. Office for National Statistics (United Kingdom). United Kingdom - England and Wales Mortality Statistics 2013.
456. Office for National Statistics (United Kingdom). United Kingdom - England and Wales Mortality Statistics 2014.
457. Office for National Statistics (United Kingdom). United Kingdom - England and Wales Mortality Statistics 2015.
458. Office for National Statistics (United Kingdom). United Kingdom - England and Wales Mortality Statistics 2016.
459. Office for National Statistics (United Kingdom). United Kingdom - England Mortality Statistics 1981.
460. Office for National Statistics (United Kingdom). United Kingdom - England Mortality Statistics 1982.
461. Office for National Statistics (United Kingdom). United Kingdom - England Mortality Statistics 1983.
462. Office for National Statistics (United Kingdom). United Kingdom - England Mortality Statistics 1984.
463. Office for National Statistics (United Kingdom). United Kingdom - England Mortality Statistics 1985.
464. Office for National Statistics (United Kingdom). United Kingdom - England Mortality Statistics 1986.
465. Office for National Statistics (United Kingdom). United Kingdom - England Mortality Statistics 1987.
466. Office for National Statistics (United Kingdom). United Kingdom - England Mortality Statistics 1988.
467. Office for National Statistics (United Kingdom). United Kingdom - England Mortality Statistics 1989.
468. Office for National Statistics (United Kingdom). United Kingdom - England Mortality Statistics 1990.
469. Office for National Statistics (United Kingdom). United Kingdom - England Mortality Statistics 1991.
470. Office for National Statistics (United Kingdom). United Kingdom - England Mortality Statistics 1992.
471. Office for National Statistics (United Kingdom). United Kingdom - England Mortality Statistics 1993.
472. Office for National Statistics (United Kingdom). United Kingdom - England Mortality Statistics 1994.
473. Office for National Statistics (United Kingdom). United Kingdom - England Mortality Statistics 1995.
474. Office for National Statistics (United Kingdom). United Kingdom - England Mortality Statistics 1996.
475. Office for National Statistics (United Kingdom). United Kingdom - England Mortality Statistics 1997.
476. Office for National Statistics (United Kingdom). United Kingdom - England Mortality Statistics 1998.

477. Office for National Statistics (United Kingdom). United Kingdom - England Mortality Statistics 1999.
478. Office for National Statistics (United Kingdom). United Kingdom - England Mortality Statistics 2000.
479. Office for National Statistics (United Kingdom). United Kingdom - England Mortality Statistics 2001.
480. Office for National Statistics (United Kingdom). United Kingdom - England Mortality Statistics 2002.
481. Office for National Statistics (United Kingdom). United Kingdom - England Mortality Statistics 2003.
482. Office for National Statistics (United Kingdom). United Kingdom - England Mortality Statistics 2004.
483. Office for National Statistics (United Kingdom). United Kingdom - England Mortality Statistics 2005.
484. Office for National Statistics (United Kingdom). United Kingdom - England Mortality Statistics 2006.
485. Office for National Statistics (United Kingdom). United Kingdom - England Mortality Statistics 2007.
486. Office for National Statistics (United Kingdom). United Kingdom - England Mortality Statistics 2008.
487. Office for National Statistics (United Kingdom). United Kingdom - England Mortality Statistics 2009.
488. Office for National Statistics (United Kingdom). United Kingdom - England Mortality Statistics 2010.
489. Office for National Statistics (United Kingdom). United Kingdom - England Mortality Statistics 2011.
490. Office for National Statistics (United Kingdom). United Kingdom - England Mortality Statistics 2012.
491. World Health Organization (WHO). WHO Mortality Database Version November 2015. Geneva, Switzerland: World Health Organization (WHO), 2015.
492. World Health Organization (WHO). WHO Mortality Database Version October 2017. Geneva, Switzerland: World Health Organization (WHO), 2017.
493. World Health Organization (WHO). WHO Mortality Database Version April 2018. Geneva, Switzerland: World Health Organization (WHO), 2018.

United States

494. National Center for Health Statistics (NCHS), Centers for Disease Control and Prevention (CDC), U.S. Department of Defense. United States NVSS Custom Mortality Data 2005 and United States Military Deaths 1980-2014.
495. National Center for Health Statistics (NCHS), Centers for Disease Control and Prevention (CDC), U.S. Department of Defense. United States NVSS Custom Mortality Data 2006 and United States Military Deaths 1980-2014.

496. National Center for Health Statistics (NCHS), Centers for Disease Control and Prevention (CDC), U.S. Department of Defense. United States NVSS Custom Mortality Data 2007 and United States Military Deaths 1980-2014.
497. National Center for Health Statistics (NCHS), Centers for Disease Control and Prevention (CDC), U.S. Department of Defense. United States NVSS Custom Mortality Data 2008 and United States Military Deaths 1980-2014.
498. National Center for Health Statistics (NCHS), Centers for Disease Control and Prevention (CDC), U.S. Department of Defense. United States NVSS Custom Mortality Data 2009 and United States Military Deaths 1980-2014.
499. National Center for Health Statistics (NCHS), Centers for Disease Control and Prevention (CDC), U.S. Department of Defense. United States NVSS Custom Mortality Data 2010 and United States Military Deaths 1980-2014.
500. National Center for Health Statistics (NCHS), Centers for Disease Control and Prevention (CDC), U.S. Department of Defense. United States NVSS Custom Mortality Data 2011 and United States Military Deaths 1980-2014.
501. National Center for Health Statistics (NCHS), Centers for Disease Control and Prevention (CDC), U.S. Department of Defense. United States NVSS Custom Mortality Data 2012 and United States Military Deaths 1980-2014.
502. National Center for Health Statistics (NCHS), Centers for Disease Control and Prevention (CDC), U.S. Department of Defense. United States NVSS Custom Mortality Data 2013 and United States Military Deaths 1980-2014.
503. National Center for Health Statistics (NCHS), Centers for Disease Control and Prevention (CDC), U.S. Department of Defense. United States NVSS Custom Mortality Data 2014 and United States Military Deaths 1980-2014.
504. National Center for Health Statistics (NCHS), Centers for Disease Control and Prevention (CDC), U.S. Department of Defense. United States NVSS Mortality Data 1980 - NBER and United States Military Deaths 1980-2014.
505. National Center for Health Statistics (NCHS), Centers for Disease Control and Prevention (CDC), U.S. Department of Defense. United States NVSS Mortality Data 1981 - NBER and United States Military Deaths 1980-2014.
506. National Center for Health Statistics (NCHS), Centers for Disease Control and Prevention (CDC), U.S. Department of Defense. United States NVSS Mortality Data 1982 - NBER and United States Military Deaths 1980-2014.
507. National Center for Health Statistics (NCHS), Centers for Disease Control and Prevention (CDC), U.S. Department of Defense. United States NVSS Mortality Data 1983 - NBER and United States Military Deaths 1980-2014.
508. National Center for Health Statistics (NCHS), Centers for Disease Control and Prevention (CDC), U.S. Department of Defense. United States NVSS Mortality Data 1984 - NBER and United States Military Deaths 1980-2014.
509. National Center for Health Statistics (NCHS), Centers for Disease Control and Prevention (CDC), U.S. Department of Defense. United States NVSS Mortality Data 1985 - NBER and United States Military Deaths 1980-2014.

510. National Center for Health Statistics (NCHS), Centers for Disease Control and Prevention (CDC), U.S. Department of Defense. United States NVSS Mortality Data 1986 - NBER and United States Military Deaths 1980-2014.
511. National Center for Health Statistics (NCHS), Centers for Disease Control and Prevention (CDC), U.S. Department of Defense. United States NVSS Mortality Data 1987 - NBER and United States Military Deaths 1980-2014.
512. National Center for Health Statistics (NCHS), Centers for Disease Control and Prevention (CDC), U.S. Department of Defense. United States NVSS Mortality Data 1988 - NBER and United States Military Deaths 1980-2014.
513. National Center for Health Statistics (NCHS), Centers for Disease Control and Prevention (CDC), U.S. Department of Defense. United States NVSS Mortality Data 1989 - NBER and United States Military Deaths 1980-2014.
514. National Center for Health Statistics (NCHS), Centers for Disease Control and Prevention (CDC), U.S. Department of Defense. United States NVSS Mortality Data 1990 - NBER and United States Military Deaths 1980-2014.
515. National Center for Health Statistics (NCHS), Centers for Disease Control and Prevention (CDC), U.S. Department of Defense. United States NVSS Mortality Data 1991 - NBER and United States Military Deaths 1980-2014.
516. National Center for Health Statistics (NCHS), Centers for Disease Control and Prevention (CDC), U.S. Department of Defense. United States NVSS Mortality Data 1992 - NBER and United States Military Deaths 1980-2014.
517. National Center for Health Statistics (NCHS), Centers for Disease Control and Prevention (CDC), U.S. Department of Defense. United States NVSS Mortality Data 1993 - NBER and United States Military Deaths 1980-2014.
518. National Center for Health Statistics (NCHS), Centers for Disease Control and Prevention (CDC), U.S. Department of Defense. United States NVSS Mortality Data 1994 - NBER and United States Military Deaths 1980-2014.
519. National Center for Health Statistics (NCHS), Centers for Disease Control and Prevention (CDC), U.S. Department of Defense. United States NVSS Mortality Data 1995 - NBER and United States Military Deaths 1980-2014.
520. National Center for Health Statistics (NCHS), Centers for Disease Control and Prevention (CDC), U.S. Department of Defense. United States NVSS Mortality Data 1996 - NBER and United States Military Deaths 1980-2014.
521. National Center for Health Statistics (NCHS), Centers for Disease Control and Prevention (CDC), U.S. Department of Defense. United States NVSS Mortality Data 1997 - NBER and United States Military Deaths 1980-2014.
522. National Center for Health Statistics (NCHS), Centers for Disease Control and Prevention (CDC), U.S. Department of Defense. United States NVSS Mortality Data 1998 - NBER and United States Military Deaths 1980-2014.
523. National Center for Health Statistics (NCHS), Centers for Disease Control and Prevention (CDC), U.S. Department of Defense. United States NVSS Mortality Data 1999 - NBER and United States Military Deaths 1980-2014.

524. National Center for Health Statistics (NCHS), Centers for Disease Control and Prevention (CDC), U.S. Department of Defense. United States NVSS Mortality Data 2000 - NBER and United States Military Deaths 1980-2014.
525. National Center for Health Statistics (NCHS), Centers for Disease Control and Prevention (CDC), U.S. Department of Defense. United States NVSS Mortality Data 2001 - NBER and United States Military Deaths 1980-2014.
526. National Center for Health Statistics (NCHS), Centers for Disease Control and Prevention (CDC), U.S. Department of Defense. United States NVSS Mortality Data 2002 - NBER and United States Military Deaths 1980-2014.
527. National Center for Health Statistics (NCHS), Centers for Disease Control and Prevention (CDC), U.S. Department of Defense. United States NVSS Mortality Data 2003 - NBER and United States Military Deaths 1980-2014.
528. National Center for Health Statistics (NCHS), Centers for Disease Control and Prevention (CDC), U.S. Department of Defense. United States NVSS Mortality Data 2004 - NBER and United States Military Deaths 1980-2014.
529. National Center for Health Statistics, Centers for Disease Control and Prevention. United States NVSS Custom Mortality Data 2015. Hyattsville, United States: National Center for Health Statistics, Centers for Disease Control and Prevention.

Uruguay

530. Ministry of Public Health (Uruguay). Uruguay Vital Registration - Deaths 1991.
531. World Health Organization (WHO). WHO Mortality Database Version September 2016. Geneva, Switzerland: World Health Organization (WHO), 2016.
532. World Health Organization (WHO). WHO Mortality Database Version October 2017. Geneva, Switzerland: World Health Organization (WHO), 2017.

Venezuela

533. World Health Organization (WHO). WHO Mortality Database Version September 2016. Geneva, Switzerland: World Health Organization (WHO), 2016.
534. World Health Organization (WHO). WHO Mortality Database Version October 2017. Geneva, Switzerland: World Health Organization (WHO), 2017.

Etiology proportion data

Armenia

1. Ministry of Health (Armenia), National Statistical Service of the Republic of Armenia. Armenia Health and Health Care 2009. Yerevan, Armenia: National Health Information Analytic Center, National Institute of Health (Armenia), 2010.

Bangladesh

2. Brooks WA, Hossain A, Goswami D, Sharmeen AT, Nahar K, Alam K, Ahmed N, Naheed A, Nair GB, Luby S, Breiman RF. Bacteremic Typhoid Fever in Children in an Urban Slum, Bangladesh. Emerg Infect Dis. 2005; 11(2): 326-9.
3. Naheed A, Ram PK, Brooks WA, Hossain MA, Parsons MB, Talukder KA, Mintz E, Luby S, Breiman RF. Burden of typhoid and paratyphoid fever in a densely populated urban community, Dhaka, Bangladesh. Int J Infect Dis. 2010; 14(Suppl 3): e93-e99.

Benin

4. Dovonou A, Adoukonou T, Sanni A, Gandaho P. [Major salmonellosis in Benin]. Med Trop (Mars). 2011; 71(6): 634-5.

Burkina Faso

5. International Vaccine Institute. Typhoid Fever Surveillance in Africa Program (TSAP) Typhoid & Salmonella Tabulations.

Chile

6. Black RE, Levine MM, Ferreccio C, Clements ML, Lanata C, Rooney J, Germanier R, Chilean Typhoid Committee. Efficacy of one or two doses of Ty21a Salmonella typhi vaccine in enteric-coated capsules in a controlled field trial. Vaccine. 1990; 8(1): 81-4.

China

7. Chinese Center for Disease Control and Prevention (CCDC). China Notifiable Infectious Diseases 2004-2012.
8. Ochiai RL, Wang X, von Seidlein L, Yang J, Bhutta ZA, Bhattacharya SK, Agtini M, Deen JL, Wain J, Kim DR, Ali M, Acosta CJ, Jodar L, Clemens JD. Salmonella Paratyphi A Rates, Asia. Emerg Infect Dis. 2005; 11(11): 1764-6.

Germany

9. Robert Koch Institute. Germany Epidemiological Infection Yearbook for Notifiable Diseases 2001. Berlin, Germany: Robert Koch Institute, 2002.
10. Robert Koch Institute. Germany Epidemiological Infection Yearbook for Notifiable Diseases 2002. Berlin, Germany: Robert Koch Institute, 2003.
11. Robert Koch Institute. Germany Epidemiological Infection Yearbook for Notifiable Diseases 2003. Berlin, Germany: Robert Koch Institute, 2004.
12. Robert Koch Institute. Germany Epidemiological Infection Yearbook for Notifiable Diseases 2004. Berlin, Germany: Robert Koch Institute, 2005.
13. Robert Koch Institute. Germany Epidemiological Infection Yearbook for Notifiable Diseases 2005. Berlin, Germany: Robert Koch Institute, 2006.
14. Robert Koch Institute. Germany Epidemiological Infection Yearbook for Notifiable Diseases 2007. Berlin, Germany: Robert Koch Institute, 2008.
15. Robert Koch Institute. Germany Epidemiological Infection Yearbook for Notifiable Diseases 2008. Berlin, Germany: Robert Koch Institute, 2009.
16. Robert Koch Institute. Germany Epidemiological Infection Yearbook for Notifiable Diseases 2009. Berlin, Germany: Robert Koch Institute, 2010.

17. Robert Koch Institute. Germany Epidemiological Infection Yearbook for Notifiable Diseases 2010. Berlin, Germany: Robert Koch Institute, 2011.
18. Robert Koch Institute. Germany Epidemiological Infection Yearbook for Notifiable Diseases 2011. Berlin, Germany: Robert Koch Institute, 2012.

Ghana

19. International Vaccine Institute. Typhoid Fever Surveillance in Africa Program (TSAP) Typhoid & Salmonella Tabulations.

Guinea-Bissau

20. International Vaccine Institute. Typhoid Fever Surveillance in Africa Program (TSAP) Typhoid & Salmonella Tabulations.

India

21. Bhattacharya SS, Das U, Choudhury BK. Occurrence and antibiogram of Salmonella Typhi and S. Paratyphi A isolated from Rourkela, Orissa. Indian J Med Res. 2011; 133(4): 431-3.
22. Gupta V, Kaur J, Chander J. An increase in enteric fever cases due to Salmonella Paratyphi A in and around Chandigarh. Indian J Med Res. 2009; 129(1): 95-8.
23. Jog S, Soman R, Singhal T, Rodrigues C, Mehta A, Dastur FD. Enteric fever in Mumbai Ã¢Â€Â“ clinical profile, sensitivity patterns and response to antimicrobials. J Assoc Physicians India. 2008; 56: 237-40.
24. Ochiai RL, Wang X, von Seidlein L, Yang J, Bhutta ZA, Bhattacharya SK, Agtini M, Deen JL, Wain J, Kim DR, Ali M, Acosta CJ, Jodar L, Clemens JD. Salmonella Paratyphi A Rates, Asia. Emerg Infect Dis. 2005; 11(11): 1764-6.
25. Vidyalakshmi K, Yashavanth R, Chakrapani M, Shrikala B, Bharathi B, Suchitra U, Dhanashree B, Dominic RMS. Epidemiological shift, seasonal variation and antimicrobial susceptibility patterns among enteric fever pathogens in South India. Trop Doct. 2008; 38(2): 89-91.

Indonesia

26. Ochiai RL, Wang X, von Seidlein L, Yang J, Bhutta ZA, Bhattacharya SK, Agtini M, Deen JL, Wain J, Kim DR, Ali M, Acosta CJ, Jodar L, Clemens JD. Salmonella Paratyphi A Rates, Asia. Emerg Infect Dis. 2005; 11(11): 1764-6.
27. Simanjuntak CH, Totosudirjo H, Haryanto P, Suprijanto E, Paleologo FP, Punjabi NH, Witham ND, Darmowigoto R, Soeprawoto, Hoffman SL. Oral immunisation against typhoid fever in Indonesia with Ty21a vaccine. Lancet. 1991; 338(8774): 1055-9.
28. Vollaard AM, Ali S, Widjaja S, Asten HAGH van, Visser LG, Surjadi C, Dissel JT van. Identification of typhoid fever and paratyphoid fever cases at presentation in outpatient clinics in Jakarta, Indonesia. Trans R Soc Trop Med Hyg. 2005; 99(6): 440-50.

Iran

29. Yousefi-Mashouf R, Moshtaghi AA. Frequency of Typhoidal and Non-Typhoidal Salmonella Species and Detection of Their Drugs Resistance Patterns. J Res Health Sci. 2007; 7(1): 49-56.

Israel

30. Meltzer E, Yossepowitch O, Sadik C, Dan M, Schwartz E. Epidemiology and Clinical Aspects of Enteric Fever in Israel. Am J Trop Med Hyg. 2006; 74(4): 540-5.

Kenya

31. Breiman RF, Cosmas L, Njuguna H, Audi A, Olack B, Ochieng JB, Wamola N, Bigogo GM, Awiti G, Tabu CW, Burke H, Williamson J, Oundo JO, Mintz ED, Feikin DR. Population-based incidence of typhoid fever in an urban informal settlement and a rural area in Kenya: implications for typhoid vaccine use in Africa. PLoS One. 2012; 7(1): e29119.
32. International Vaccine Institute. Typhoid Fever Surveillance in Africa Program (TSAP) Typhoid & Salmonella Tabulations.

Madagascar

33. International Vaccine Institute. Typhoid Fever Surveillance in Africa Program (TSAP) Typhoid & Salmonella Tabulations.

Nepal

34. Baker S, Holt KE, Clements ACA, Karkey A, Arjyal A, Boni MF, Dongol S, Hammond N, Koirala S, Duy PT, Nga TVT, Campbell JI, Dolecek C, Basnyat B, Dougan G, Farrar JJ. Combined high-resolution genotyping and geospatial analysis reveals modes of endemic urban typhoid fever transmission. Open Biol. 2011; 1(2): 110008.
35. Blacksell SD, Sharma NP, Phumratanaprapin W, Jenjaroen K, Peacock SJ, White NJ, Pukrittayakamee S, Day NPJ. Serological and blood culture investigations of Nepalese fever patients. Trans R Soc Trop Med Hyg. 2007; 101(7): 686-90.
36. Karkey A, Arjyal A, Anders KL, Boni MF, Dongol S, Koirala S, My PVT, Nga TVT, Clements ACA, Holt KE, Duy PT, Day JN, Campbell JI, Dougan G, Dolecek C, Farrar J, Basnyat B, Baker S. The Burden and Characteristics of Enteric Fever at a Healthcare Facility in a Densely Populated Area of Kathmandu. PLoS One. 2010; 5(11).
37. Malla T, Malla KK, Thapalial A, Shaw C. Enteric fever: a retrospective 6-year analysis of 82 paediatric cases in a teaching hospital. Kathmandu Univ Med J (KUMJ). 2007; 5(2): 181-7.
38. Maskey AP, Basnyat B, Thwaites GE, Campbell JI, Farrar JJ, Zimmerman MD. Emerging trends in enteric fever in Nepal: 9124 cases confirmed by blood culture 1993-2003. Trans R Soc Trop Med Hyg. 2008; 102(1): 91-5.
39. Pokharel P, Rai SK, Karki G, Katuwal A, Vitrakoti R, Shrestha SK. Study of enteric fever and antibiogram of Salmonella isolates at a teaching hospital in Kathmandu Valley. Nepal Med Coll J. 2009; 11(3): 176-8.
40. Prajapati B, Rai GK, Rai SK, Upreti HC, Thapa M, Singh G, Shrestha RM. Prevalence of Salmonella typhi and paratyphi infection in children: a hospital based study. Nepal Med Coll J. 2008; 10(4): 238-41.

Nigeria

41. Akinyemi KO, Bamiro BS, Coker AO. Salmonellosis in Lagos, Nigeria: Incidence of Plasmodium falciparum-associated Co-infection, Patterns of Antimicrobial Resistance, and Emergence of Reduced Susceptibility to Fluoroquinolones. J Health Popul Nutr. 2007; 25(3): 351-8.

Pakistan

42. Butt T, Ahmad RN, Salman M, Kazmi SY. Changing trends in drug resistance among typhoid salmonellae in Rawalpindi, Pakistan. East Mediterr Health J. 2005; 11(5-6): 1038-44.
43. Hasan R, Zafar A, Abbas Z, Mahraj V, Malik F, Zaidi A. Antibiotic resistance among Salmonella enterica serovars Typhi and Paratyphi A in Pakistan (2001-2006). J Infect Dev Ctries. 2008; 2(04): 289-94.
44. Khan MI, Sahito SM, Khan MJ, Wassan SM, Shaikh AW, Maheshwari AK, Acosta CJ, Galindo CM, Ochiai RL, Rasool S, Peerwani S, Puri MK, Ali M, Zafar A, Hassan R, von Seidlein L, Clemens JD, Nizami SQ, Bhutta ZA. Enhanced disease surveillance through private health care sector cooperation in Karachi, Pakistan: experience from a vaccine trial. Bull World Health Organ. 2006; 84(1): 72-7.
45. Khan MI, Soofi SB, Ochiai RL, Khan MJ, Sahito SM, Habib MA, Puri MK, Von Seidlein L, Park JK, You YA, Ali M, Nizami SQ, Acosta CJ, Sack RB, Clemens JD, Bhutta ZA. Epidemiology, clinical presentation, and patterns of drug resistance of Salmonella Typhi in Karachi, Pakistan. J Infect Dev Ctries. 2012; 6(10): 704-14.
46. Ochiai RL, Wang X, von Seidlein L, Yang J, Bhutta ZA, Bhattacharya SK, Agtini M, Deen JL, Wain J, Kim DR, Ali M, Acosta CJ, Jodar L, Clemens JD. Salmonella Paratyphi A Rates, Asia. Emerg Infect Dis. 2005; 11(11): 1764-6.
47. Owais A, Sultana S, Zaman U, Rizvi A, Zaidi AKM. Incidence of typhoid bacteremia in infants and young children in southern coastal Pakistan. Pediatr Infect Dis J. 2010; 29(11): 1035-9.
48. Siddiqui FJ, Rabbani F, Hasan R, Nizami SQ, Bhutta ZA. Typhoid fever in children: some epidemiological considerations from Karachi, Pakistan. Int J Infect Dis. 2006; 10(3): 215-22.

Senegal

49. Seydi M, Soumare M, Sow AI, Diop BM, Sow PS. Aspects actuels des bactÃƒÂ©riÃƒÂ©mies ÃƒÂ Salmonella ÃƒÂ la clinique des maladies infectieuses Ibrahima Diop Mar du centre hospitalier national de Fann (SÃƒÂ©nÃƒÂ©gal). Med Mal Infect. 2005; 35(1): 23-7.

Singapore

50. Ty AU, Ang GY, Ang LW, James L, Goh KT. Changing epidemiology of enteric fevers in Singapore. Ann Acad Med Singapore. 2010; 39(12): 889.

South Africa

51. National Institute for Communicable Diseases (South Africa). South Africa GERMS-SA Typhoid and Paratyphoid Tabulations 2003.
52. National Institute for Communicable Diseases (South Africa). South Africa GERMS-SA Typhoid and Paratyphoid Tabulations 2004.
53. National Institute for Communicable Diseases (South Africa). South Africa GERMS-SA Typhoid and Paratyphoid Tabulations 2005.
54. National Institute for Communicable Diseases (South Africa). South Africa GERMS-SA Typhoid and Paratyphoid Tabulations 2006.
55. National Institute for Communicable Diseases (South Africa). South Africa GERMS-SA Typhoid and Paratyphoid Tabulations 2007.

56. National Institute for Communicable Diseases (South Africa). South Africa GERMS-SA Typhoid and Paratyphoid Tabulations 2008.
57. National Institute for Communicable Diseases (South Africa). South Africa GERMS-SA Typhoid and Paratyphoid Tabulations 2009.
58. National Institute for Communicable Diseases (South Africa). South Africa GERMS-SA Typhoid and Paratyphoid Tabulations 2010.
59. National Institute for Communicable Diseases (South Africa). South Africa GERMS-SA Typhoid and Paratyphoid Tabulations 2011.
60. National Institute for Communicable Diseases (South Africa). South Africa GERMS-SA Typhoid and Paratyphoid Tabulations 2012.
61. National Institute for Communicable Diseases (South Africa). South Africa GERMS-SA Typhoid and Paratyphoid Tabulations 2013.
62. National Institute for Communicable Diseases (South Africa). South Africa GERMS-SA Typhoid and Paratyphoid Tabulations 2014.

Spain

63. Gil R, ÃƒÂlvarez JL, GÃƒÂ³mez C, ÃƒÂlvaro A, Gil ÃƒÂ. Epidemiology of typhoid and paratyphoid fever hospitalizations in Spain (1997-2005). Hum Vaccin. 2013; 5(6): 420-4.

Tanzania

64. International Vaccine Institute. Typhoid Fever Surveillance in Africa Program (TSAP) Typhoid & Salmonella Tabulations.

Case fatality data

Afghanistan

1. Afghanistan National Public Health Institute (ANPHI), World Health Organization (WHO). Afghanistan Disease Early Warning System Annual Report 2010. Kabul, Afghanistan: Ministry of Public Health (Afghanistan).

Argentina

2. Directorate of Epidemiology, Ministry of Health (Argentina). Argentina Annual Epidemiological Bulletin 2003-2005. Buenos Aires, Argentina: Ministry of Health (Argentina).

Bangladesh

3. Brooks WA, Hossain A, Goswami D, Sharmeen AT, Nahar K, Alam K, Ahmed N, Naheed A, Nair GB, Luby S, Breiman RF. Bacteremic Typhoid Fever in Children in an Urban Slum, Bangladesh. Emerg Infect Dis. 2005; 11(2): 326-9.

Benin

4. Dovonou A, Adoukonou T, Sanni A, Gandaho P. [Major salmonellosis in Benin]. Med Trop (Mars). 2011; 71(6): 634-5.

Brazil

5. Ministry of Health (Brazil). Brazil Information System for Notifiable Diseases - Typhoid Fever.

Burkina Faso

6. Ministry of Health (Burkina Faso). Burkina Faso Health Statistical Yearbook 2004. Ouagadougou, Burkina Faso: Ministry of Health (Burkina Faso), 2005.

Cambodia

7. Ministry of Health (Cambodia). Cambodia National Health Statistics 2006. Phnom Penh, Cambodia: Ministry of Health (Cambodia), 2007.
8. Ministry of Health (Cambodia). Cambodia National Health Statistics 2007. Phnom Penh, Cambodia: Ministry of Health (Cambodia), 2008.
9. Ministry of Health (Cambodia). Cambodia National Health Statistics 2008. Phnom Penh, Cambodia: Ministry of Health (Cambodia).
10. Ministry of Health (Cambodia). Cambodia National Health Statistics 2009. Phnom Penh, Cambodia: Ministry of Health (Cambodia).
11. Ministry of Health (Cambodia). Cambodia National Health Statistics 2010. Phnom Penh, Cambodia: Ministry of Health (Cambodia).
12. Ministry of Health (Cambodia). Cambodia National Health Statistics 2011. Phnom Penh, Cambodia: Ministry of Health (Cambodia).

China

13. Chinese Center for Disease Control and Prevention (CCDC). China Notifiable Infectious Diseases 2004-2012.
14. Dong B-Q, Yang J, Wang X-Y, Gong J, von Seidlein L, Wang M-L, Lin M, Liao H-Z, Ochiai RL, Xu Z-Y, Jodar L, Clemens JD. Trends and disease burden of enteric fever in Guangxi province, China, 1994-2004. Bull World Health Organ. 2010; 88(9): 689-96.

Cuba

15. National Office of Statistics (Cuba). Cuba Statistical Yearbook 2002. Havana, Cuba: National Office of Statistics (Cuba), 2003

India

16. Advent Healthcare Group, Central Bureau of Health Intelligence (India), World Health Organization (WHO). India National Health Profile 2005. New Delhi, India: Central Bureau of Health Intelligence (India), 2006.
17. Central Bureau of Health Intelligence (India), World Health Organization (WHO). India National Health Profile 2006. New Delhi, India: Central Bureau of Health Intelligence (India), 2007.
18. Central Bureau of Health Intelligence (India). India National Health Profile 2007. New Delhi, India: Central Bureau of Health Intelligence (India), 2008.

19. Central Bureau of Health Intelligence (India). India National Health Profile 2008. New Delhi, India: Central Bureau of Health Intelligence (India), 2009.
20. Central Bureau of Health Intelligence (India). India National Health Profile 2009. New Delhi, India: Central Bureau of Health Intelligence (India), 2010.
21. Central Bureau of Health Intelligence (India). India National Health Profile 2010. New Delhi, India: Central Bureau of Health Intelligence (India), 2011.
22. Central Bureau of Health Intelligence (India). India National Health Profile 2011. New Delhi, India: Central Bureau of Health Intelligence (India), 2012.

Kenya

23. Breiman RF, Cosmas L, Njuguna H, Audi A, Olack B, Ochieng JB, Wamola N, Bigogo GM, Awiti G, Tabu CW, Burke H, Williamson J, Oundo JO, Mintz ED, Feikin DR. Population-based incidence of typhoid fever in an urban informal settlement and a rural area in Kenya: implications for typhoid vaccine use in Africa. PLoS One. 2012; 7(1): e29119.

Nigeria

24. Federal Ministry of Health (Nigeria), National Bureau of Statistics (Nigeria). Nigeria Facts and Figures 2008. Abuja, Nigeria: National Bureau of Statistics (Nigeria).

Pakistan

25. Owais A, Sultana S, Zaman U, Rizvi A, Zaidi AKM. Incidence of typhoid bacteremia in infants and young children in southern coastal Pakistan. Pediatr Infect Dis J. 2010; 29(11): 1035-9.

Philippines

26. Department of Health (Philippines). Philippines Health Statistics 2004. Manila, Philippines: Department of Health (Philippines).

Qatar

27. Hamad Medical Corporation (Qatar), Ministry of Public Health (Qatar). Qatar Annual Health Report 2004. Doha, Qatar: Hamad Medical Corporation (Qatar), 2005.
28. Hamad Medical Corporation (Qatar), National Health Authority (Qatar). Qatar Annual Health Report 2005. Doha, Qatar: Hamad Medical Corporation (Qatar), 2006.
29. Hamad Medical Corporation (Qatar), National Health Authority (Qatar). Qatar Annual Health Report 2006. Doha, Qatar: Hamad Medical Corporation (Qatar), 2007.
30. Hamad Medical Corporation (Qatar), Supreme Council of Health (Qatar). Qatar Annual Health Report 2011. Doha, Qatar: Hamad Medical Corporation (Qatar), 2012.

Spain

31. Gil R, ÃƒÂ�lvarez JL, GÃƒÂ³mez C, ÃƒÂ�lvaro A, Gil ÃƒÂ�. Epidemiology of typhoid and paratyphoid fever hospitalizations in Spain (1997-2005). Hum Vaccin. 2013; 5(6): 420-4.

Sri Lanka

32. Ministry of Health (Sri Lanka). Sri Lanka Annual Health Bulletin 2000.
33. Ministry of Health (Sri Lanka). Sri Lanka Annual Health Bulletin 2001.

34. Ministry of Health (Sri Lanka). Sri Lanka Annual Health Statistics 2007. Colombo, Sri Lanka: Ministry of Health (Sri Lanka).

Sudan

35. Central Bureau of Statistics (Sudan). Sudan Statistical Yearbook 2009. Khartoum, Sudan: Central Bureau of Statistics (Sudan).

D. References

1 Antillón M, Warren JL, Crawford FW, *et al.* The burden of typhoid fever in low- and middle-income countries: A meta-regression approach. *PLoS Negl Trop Dis* 2017; **11**: e0005376.

2 Crump JA, Luby SP, Mintz ED. The global burden of typhoid fever. *Bull World Health Organ* 2004; **82**: 346–53.

3 Buckle GC, Walker CLF, Black RE. Typhoid fever and paratyphoid fever: Systematic review to estimate global morbidity and mortality for 2010. *J Glob Health* 2012; **2**. DOI:10.7189/jogh.02.010401.

4 Mogasale V, Ramani E, Mogasale VV, Park J. What proportion of Salmonella Typhi cases are detected by blood culture? A systematic literature review. *Ann Clin Microbiol Antimicrob* 2016; **15**: 32.

5 Flaxman A, Vos T, Murray C, editors. An Integrative Metaregression Framework for Descriptive Epidemiology. Seattle: University of Washington Press, 2015.

6 GBD 2017 Disease and Injury Incidence and Prevalence Collaborators. Global, regional, and national incidence, prevalence, and years lived with disability for diseases and injuries for 195 countries, 1990–2017: a systematic analysis for the Global Burden of Disease Study 2017. *Lancet* In Press.

7 Nyaga VN, Arbyn M, Aerts M. Metaprop: a Stata command to perform meta-analysis of binomial data. *Arch Public Health* 2014; **72**. DOI:10.1186/2049-3258-72-39.

8 Chinese Center for Disease Control and Prevention (CCDC). China Notifiable Infectious Diseases 2004-2012. 2012.

9 GBD 2016 Causes of Death Collaborators. Global, regional, and national age-sex specific mortality for 264 causes of death, 1980-2016: a systematic analysis for the Global Burden of Disease Study 2016. *Lancet Lond Engl* 2017; **390**: 1151–210.

10 Vos T, Flaxman AD, Naghavi M, *et al.* Years lived with disability (YLDs) for 1160 sequelae of 289 diseases and injuries 1990-2010: a systematic analysis for the Global Burden of Disease Study 2010. *Lancet Lond Engl* 2012; **380**: 2163–96.

11 Global Burden of Disease Study 2013 Collaborators. Global, regional, and national incidence, prevalence, and years lived with disability for 301 acute and chronic diseases and injuries in 188 countries, 1990-2013: a systematic analysis for the Global Burden of Disease Study 2013. *Lancet Lond Engl* 2015; **386**: 743–800.

12 Vos T, Allen C, Arora M, *et al.* Global, regional, and national incidence, prevalence, and years lived with disability for 310 diseases and injuries, 1990–2015: a systematic analysis for the Global Burden of Disease Study 2015. *The Lancet* 2016; **388**: 1545–602.

13 GBD 2016 Disease and Injury Incidence and Prevalence Collaborators. Global, regional, and national incidence, prevalence, and years lived with disability for 328 diseases and injuries for 195 countries, 1990-2016: a systematic analysis for the Global Burden of Disease Study 2016. *Lancet Lond Engl* 2017; **390**: 1211–59.

14 Mogasale V, Maskery B, Ochiai RL, *et al.* Burden of typhoid fever in low-income and middle-income countries: a systematic, literature-based update with risk-factor adjustment. *Lancet Glob Health* 2014; **2**: e570-580.

15 Kim J-H, Mogasale V, Im J, Ramani E, Marks F. Updated estimates of typhoid fever burden in sub-Saharan Africa. *Lancet Glob Health* 2017; **5**: e969.
